# FAM50A promotes esophageal squamous cell carcinoma progression via splicing-dependent oncogenic circSDK1- and DUSP22-mediated MAPK/FAK activation

**DOI:** 10.1038/s41421-026-00927-z

**Published:** 2026-10-20

**Authors:** Yan Bian, Ye Gao, Xin Chang, Bo Tian, Qianqian Meng, Lei Xin, Han Lin, Wei Wang, Zhaoshen Li, Luowei Wang

**Affiliations:** 1 Department of Gastroenterology, Changhai Hospital, Naval Medical University, Shanghai, China; 2 Changhai Clinical Research Unit, Changhai hospital, Naval Medical University, Shanghai, China; 3 National Key Laboratory of Immunity and Inflammation, Naval Medical University, Shanghai, China; 4 Digestive Endoscopic Center, Shanghai Sixth People’s Hospital Affiliated to Shanghai Jiao Tong University School of Medicine, Shanghai, China

**Keywords:** Oesophageal cancer, Oncogenes, Drug development

## Abstract

Dysregulated splicing factors play critical roles in tumor progression. Here, we identify family with sequence similarity 50 member A (FAM50A) as a pivotal oncogenic splicing factor involved in esophageal squamous cell carcinoma (ESCC). Multi-omics and multi-cohort analyses revealed that FAM50A is upregulated in ESCC and correlates with a poor prognosis. Functional studies demonstrated that FAM50A promotes ESCC proliferation and migration both in vitro and in vivo, primarily through the activation of mitogen-activated protein kinases (MAPKs) and focal adhesion kinase (FAK). We further characterized the FAM50A-binding RNA motif and established its transcriptome-wide splicing landscape. Notably, we identified a novel FAM50A-dependent backsplicing event that generates circSDK1, which originates from exons 2–4 of the sidekick cell adhesion molecule 1 (SDK1) pre-mRNA. Mechanistically, circSDK1 binds to dual-specificity phosphatase 22 (DUSP22) and occupies its phosphatase domain, inhibiting the DUSP22-mediated dephosphorylation of MAPKs and FAK and thereby sustaining their activation. Furthermore, the transcription factor TFAP2C increased FAM50A expression. Finally, we identified rifabutin as a small-molecule inhibitor of FAM50A and confirmed its anti-tumor efficacy in cell-derived xenograft, patient-derived organoid, and patient-derived xenograft models. In summary, FAM50A promotes ESCC progression via the FAM50A/circSDK1/DUSP22 axis, leading to MAPKs and FAK activation. Genetic or pharmacological targeting of FAM50A with rifabutin represents a promising therapeutic strategy for ESCC.

## Introduction

Esophageal cancer (EC) is the 11th most prevalent malignant tumor and the 7th most lethal malignant tumor globally, with an overall 5-year survival rate of less than 30%^1–3^. The two main pathological types of EC are esophageal squamous cell carcinoma (ESCC) and esophageal adenocarcinoma (EAC), with ESCC accounting for more than 85% of cases^4,5^. Currently, the majority of patients with ESCC are diagnosed at an advanced stage^6^. Despite comprehensive treatment strategies, including surgery, chemotherapy, radiotherapy, and immunotherapy, a proportion of patients still have a high recurrence risk^7^. The investigation of the cellular and molecular mechanisms that underlie ESCC progression has the potential to identify novel targets and treatment strategies for ESCC therapy, with benefits that include reducing the recurrence risk and improving the prognosis for patients^8^.

Alternative splicing (AS), including canonical AS (linear splicing) and backsplicing (circular splicing), is a critical step in the post-transcriptional processing of pre-mRNA^9,10^. Canonical AS can produce diverse transcripts by including or excluding one or more exons or other sequences, thereby greatly expanding the transcriptome and proteome and ensuring normal cell differentiation and organ development^11^. Backsplicing is a process that can yield circular RNA (circRNA)^12^. CircRNAs can perform biological functions that differ from those of their parent mRNAs, including acting as miRNA sponges, interacting with proteins, and encoding polypeptides^13^. Dysregulated AS is closely related to the hallmarks of cancer, including cancer initiation, promotion, progression, metastasis, and therapeutic resistance^14–17^. AS regulation relies on the interaction between *trans*-acting splicing factors and *cis*-acting RNA elements^18^. Splicing factors typically drive malignant behavior in tumors through either recurrent mutations or alterations in expression^9,19,20^. Recurrent splicing factor mutations are common in hematological malignancies^21^. Conversely, altered splicing factor levels and copy number changes are particularly prominent in solid tumors^22^. Dysregulated splicing factors can drive widespread, dynamic, and aberrant AS events, thereby regulating malignant tumor behavior^11^. The development of therapeutic strategies targeting dysregulated splicing factors is a promising approach for achieving precision cancer treatment.

Family with sequence similarity 50 member A (FAM50A) is a component of the spliceosome that is crucial for its assembly and catalytic activity. Lee et al. reported rare FAM50A missense variants in patients with Armfield X-chromosome-linked intellectual disability (XLID) syndrome^23^. Using a *fam50a* knockout zebrafish model, they confirmed that Armfield XLID syndrome is a spliceosomopathy caused by FAM50A mutations and is associated with aberrant pre-mRNA splicing during development. Sun et al. found that Kaposi’s sarcoma-associated herpesvirus can reprogram host AS via FAM50A and drive oncogenic cellular transformation^24^. Moreover, studies have demonstrated that FAM50A plays a significant role in tumor metastasis^25–27^. Chen et al. discovered that FAM50A can drive breast cancer brain metastasis through an interaction with C9ORF78 to increase asparagine synthetase transcription^28^. However, research into the mechanisms by which FAM50A regulates AS during tumor progression is currently lacking. In addition, the transcriptome-wide landscape of AS and RNA-binding sites for FAM50A remains unclear. Furthermore, targeting splicing factors has emerged as a promising therapeutic strategy for cancer treatment. For instance, H3B-8800 is an SF3B1 inhibitor that is currently being tested in phase I trials for the treatment of various cancers^29^. Consequently, the development of small-molecule inhibitors targeting FAM50A is conducive to the clinical translation of oncogenic targets.

In this study, we identified a role for the pivotal oncogenic splicing factor FAM50A in ESCC progression using multi-cohort and multi-omics data. Furthermore, we employed RNA sequencing (RNA-seq) and RNA immunoprecipitation sequencing (RIP-seq) analyses to elucidate the landscape of FAM50A-dependent AS and to identify the RNA-binding motifs and transcriptome-wide binding sites of FAM50A. Crucially, we elucidated the molecular mechanism by which FAM50A promotes ESCC progression through mitogen-activated protein kinases (MAPKs) and focal adhesion kinase (FAK) activation. Finally, we screened and identified rifabutin, a small-molecule inhibitor targeting FAM50A, which exerts a synergistic effect with cisplatin. Our study revealed that FAM50A is a significant oncogenic splicing factor and a potential therapeutic target in ESCC.

## Results

### Elevated FAM50A expression is associated with progression and a poor prognosis in patients with ESCC

**Fig. 1:**
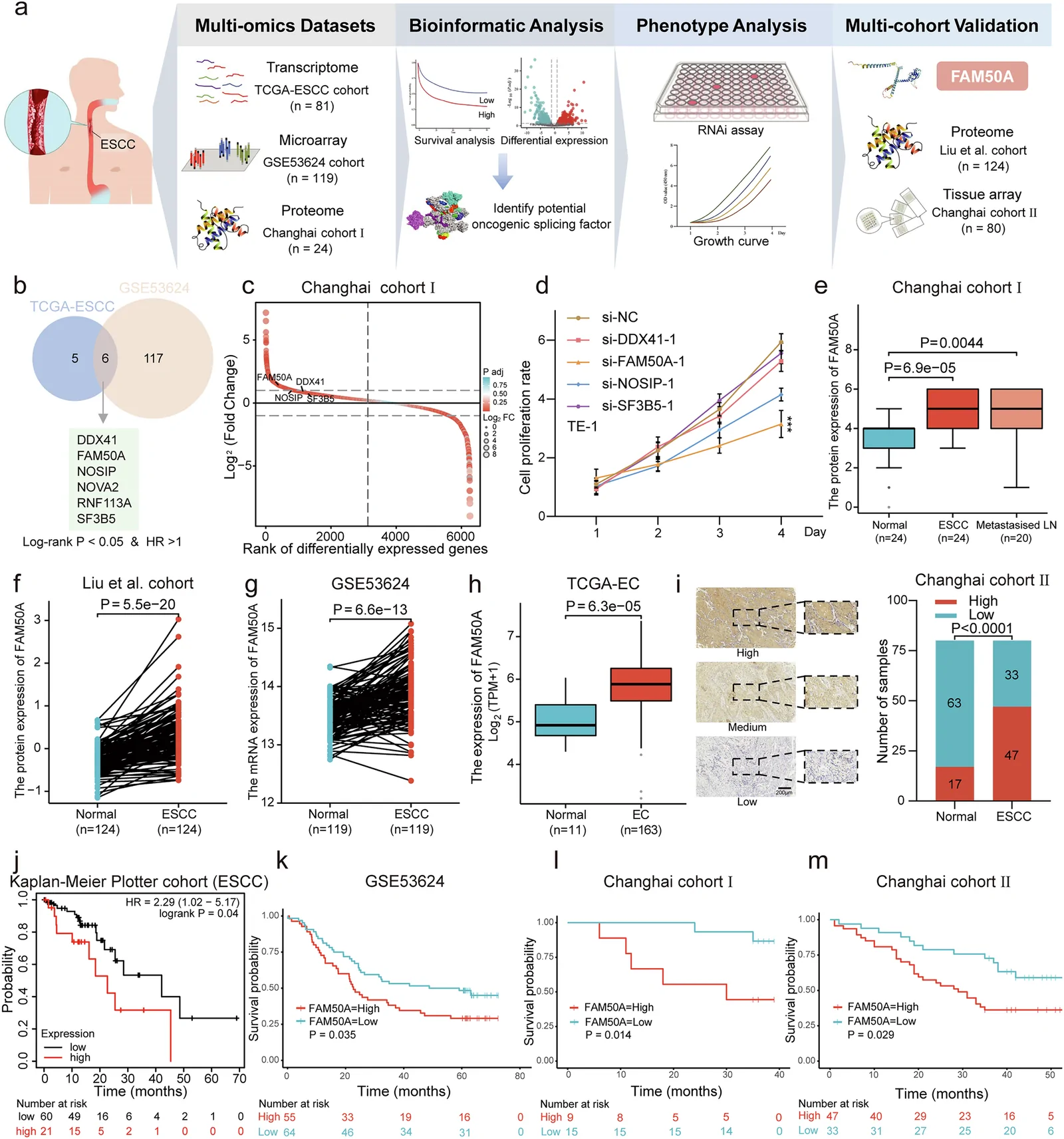
Elevated FAM50A expression is associated with progression and a poor prognosis for patients with ESCC. **a** Flowchart for identifying pivotal oncogenic splicing factors involved in ESCC progression. **b** The intersection of splicing factors related to an unfavorable prognosis in the GSE53624 and TCGA-ESCC cohorts. **c** The protein expression ranking of splicing factors in Changhai cohort I. **d** RNAi assay of the four candidate splicing factors. **e** FAM50A protein expression was analyzed in Changhai cohort I. **f** FAM50A protein expression was analyzed in the Liu et al. cohort. **g** FAM50A mRNA expression was analyzed in the GSE53624 cohort. **h** FAM50A mRNA expression was analyzed in TCGA-EC cohort. **i** Statistical analysis of FAM50A expression detected using immunohistochemical staining in Changhai cohort II. **j** Kaplan‒Meier analysis of the correlation between FAM50A expression and the overall survival of patients in TCGA-ESCC cohort. **k** Kaplan‒Meier analysis of the correlation between FAM50A expression and the overall survival of patients in the GSE53624 cohort. **l** Kaplan‒Meier analysis of the correlation between FAM50A expression and the overall survival of patients in the Changhai cohort I. **m** Kaplan‒Meier analysis of the correlation between FAM50A expression and the overall survival of patients in Changhai cohort II. *P* values were obtained using two-tailed unpaired Student’s *t* tests (**d**, **e**, **h**), two-tailed paired Student’s *t* test (**f**, **g**), chi-square test (**i**), and log-rank tests (**j**–**m**). The optimal cut-off value in the Kaplan‒Meier analysis was determined using the minimum *P* value method. The data are presented as the means ± SDs, unless stated otherwise. *P < 0.05, ** *P* < 0.01, and ***P < 0.001.

We first identified survival-related splicing factors in ESCC by analyzing the prognostic values of 274 known splicing factors in the GSE53624 and The Cancer Genome Atlas-ESCC (TCGA-ESCC) cohorts (Fig. 1a, b). We identified six splicing factors related to an unfavorable prognosis in both cohorts. Furthermore, we performed a proteomic analysis of matched cancer tissues, paracancerous tissues, and metastatic lymph nodes from 24 patients with metastatic ESCC (Changhai cohort I; Fig. 1c). The results showed that the expression of four splicing factors was aberrantly upregulated in ESCC tissues. We then conducted an RNA interference screen of four splicing factors and found that FAM50A had the most significant inhibitory effect on ESCC cell proliferation (Fig. 1d; Supplementary Fig. S1a, b). FAM50A expression was commonly upregulated at both the RNA and protein levels in ESCC tissues compared with normal tissues from several cohorts (Fig. 1e–g). Notably, FAM50A expression was also upregulated in the metastatic lymph node tissues of patients with ESCC (Fig. 1e; Supplementary Fig. S1c, d). In addition, the expression of FAM50A was generally upregulated in TCGA-EC cohort, whether the tumor tissue was ESCC or EAC, and in paired samples (Fig. 1; Supplementary Fig. S1e–i). We performed immunohistochemistry on 80 paired ESCC and normal tissues from an independent ESCC cohort (Changhai cohort II) to evaluate the clinical significance of FAM50A in ESCC. Immunohistochemical staining revealed the significant upregulation of FAM50A expression in ESCC (Fig. 1i; Supplementary Fig. S1j). In addition, FAM50A expression was generally upregulated in a variety of ESCC cell lines and one EAC cell line (BE-3) compared with the normal esophageal epithelial cell line (Supplementary Fig. S1k). Moreover, we further validated the prognostic value of FAM50A in several independent cohorts and observed a significantly poor prognosis (Fig. 1j–m; Supplementary Fig. S1l). Collectively, these results demonstrate that upregulated FAM50A expression is associated with poor prognosis for patients with ESCC.

### FAM50A contributes to the malignant phenotypes of ESCC through MAPKs and FAK activation

**Fig. 2:**
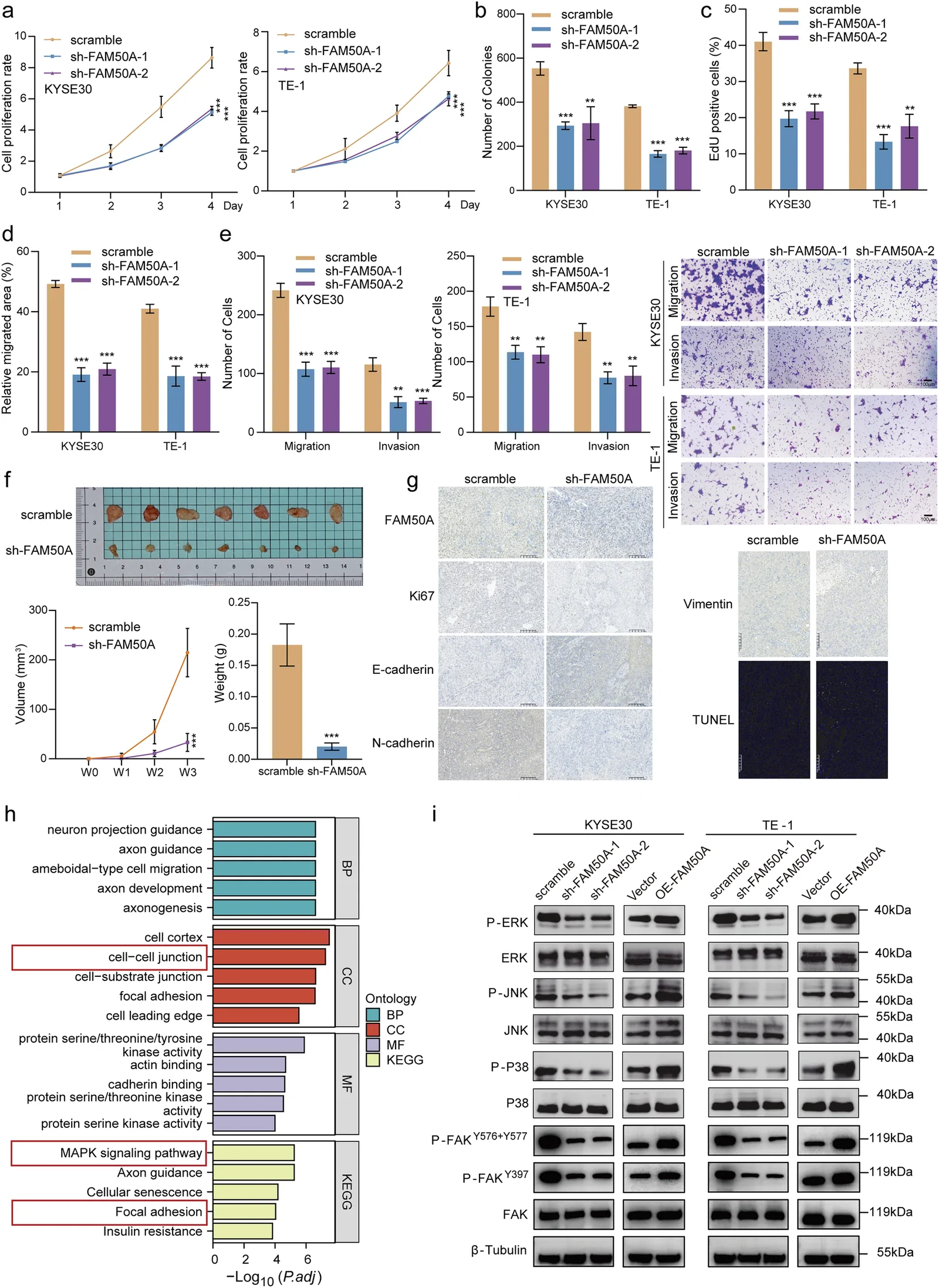
FAM50A contributes to the malignant phenotypes of ESCC through MAPKs and FAK activation. **a**–**e** Cell proliferation assays (**a**), clonogenic assays (**b**), EdU assays (**c**), wound healing assays (**d**), and Transwell assays (**e**) were performed in ESCC cell lines with FAM50A knockdown and the results were compared with the corresponding control cells (*n* = 3 for the clonogenic assay, EdU assay, wound healing assay, and Transwell assay, and *n* = 6 biologically independent experiments for the cell proliferation assay). **f** KYSE30 CDXs were dissected from nude mice (*n* = 7). The growth curves of the tumor volumes were calculated for the CDX models (*n* = 7). The weights of CDXs dissected from nude mice are shown (*n* = 7). **g** Immunohistochemical staining was conducted to assess the expression levels of Ki67, E-cadherin, N-cadherin, and Vimentin, and a TUNEL assay was conducted to quantify the number of apoptotic cells in the tumors. Scale bars, 200 μm. **h** Gene set enrichment analysis was conducted on differentially expressed genes in the RNA-seq data from KYSE30 cells after FAM50A knockdown. **i** The levels of phosphorylated MAPKs and FAK were measured by western blotting. FAM50A was knocked down or overexpressed in KYSE30 or TE-1 cells. *P* values were obtained using a two-tailed unpaired Student’s *t* test (**a**–**e**, **f**), and the results are presented as the means ± SDs. *P < 0.05, **P < 0.01, and ***P < 0.001.

We established ESCC cell lines with either stable overexpression or knockdown of FAM50A relative to its basal expression level to explore the role of FAM50A in ESCC (Supplementary Fig. S2a). We performed growth curve and clonogenic assays and found that silencing FAM50A expression significantly reduced the proliferation of ESCC cells, whereas overexpressing FAM50A had the opposite effect (Fig. 2a, b; Supplementary Figs. S2b, S3a, b). The results of the 5-ethynyl-2′-deoxyuridine (EdU) assay showed that silencing FAM50A decreased the number of EdU-positive cells, whereas overexpressing FAM50A increased the number of EdU-positive cells (Fig. 2c, Supplementary Figs. S2c and S3c). Furthermore, wound healing and Transwell assays revealed that ESCC cells with stable FAM50A knockdown exhibited limited migration and invasion abilities (Fig. 2d, e; Supplementary Fig. S2d). Conversely, FAM50A overexpression led to a significant increase in the motility of ESCC cells (Supplementary Fig. S3d, e). Subsequently, we investigated the role of FAM50A in tumorigenesis using cell line-derived xenograft (CDX) models. KYSE30 cells with FAM50A knockdown and the corresponding control cells were separately injected subcutaneously into nude mice. FAM50A knockdown in KYSE30 cells resulted in a significant reduction in the tumor growth rate and tumor weight (Fig. 2f). We also found that FAM50A knockdown reduced the Ki67 index (Fig. 2g; Supplementary Fig. S4a, b). In addition, immunohistochemistry of the tumor demonstrated that FAM50A knockdown diminished the epithelial‒mesenchymal transition (EMT) in the tumor (Fig. 2g; Supplementary Fig. S4c–e). Furthermore, FAM50A knockdown contributed to apoptosis in CDX models, as detected using a TUNEL assay (Fig. 2g; Supplementary Fig. S4f). These results suggest that FAM50A has oncogenic potential in ESCC.

We performed RNA-seq on FAM50A knockdown and control KYSE30 cells to further explore the function of FAM50A in ESCC. Gene set enrichment analysis revealed that FAM50A target genes were enriched in specific biological processes, including the MAPK signaling pathway, focal adhesion, and cell‒cell junctions (Fig. 2h). As shown in Fig. 2i, the phosphorylation levels of proteins in the three subgroups in the MAPK pathway were all decreased in the FAM50A knockdown groups of KYSE30 and TE-1 cells but were all increased in the FAM50A-overexpressing groups. Moreover, our findings indicated that FAM50A could increase FAK phosphorylation. The western blot results showed that FAM50A overexpression or knockdown resulted in an increase or decrease, respectively, in the level of FAK phosphorylation at the Y576/Y577 and Y397 sites (Fig. 2i). Furthermore, the adhesion assay revealed that FAM50A knockdown cells exhibited diminished attachment to the extracellular matrix (ECM), whereas the FAM50A overexpression cells displayed increased adhesion (Supplementary Fig. S5a). We subsequently investigated the effect of FAM50A on anoikis following the detachment of ESCC cells from the ECM during metastasis. We found that FAM50A knockdown significantly increased the anoikis rate of ESCC cells in suspension culture, as shown by the results of a caspase-3 activity assay (Supplementary Fig. S5b) and a trypan blue exclusion test of cell viability (Supplementary Fig. S5c). As demonstrated in Supplementary Fig. S5d, FAM50A knockdown resulted in significant increases in the levels of cleaved PARP and cleaved caspase-3 in ESCC cells in suspension culture. Conversely, FAM50A overexpression enhanced the anoikis resistance of ESCC cells in suspension culture (Supplementary Fig. S5b–d). The enriched cell‒cell junctions suggested by the gene set enrichment analysis are usually closely related to the EMT. As shown in Supplementary Fig. S5e, FAM50A overexpression resulted in the loss of an epithelial cell marker (E-cadherin) and the concomitant upregulation of mesenchymal cell markers (N-cadherin and Vimentin) in ESCC cells. Conversely, FAM50A knockdown had the opposite effect. Furthermore, we found that FAM50A knockdown had no effect on the phosphorylation of AKT (T308/S473) or NF-κB p65 (S536), verifying the specificity of FAM50A-mediated MAPK and FAK pathway activation (Supplementary Fig. S5f). These results suggested that FAM50A may enhance the entire process of metastasis, including metastasis initiation, dissemination, and colonization in ESCC.

### Identifying the transcriptome-wide landscape of FAM50A binding sites and alternative splicing events

**Fig. 3:**
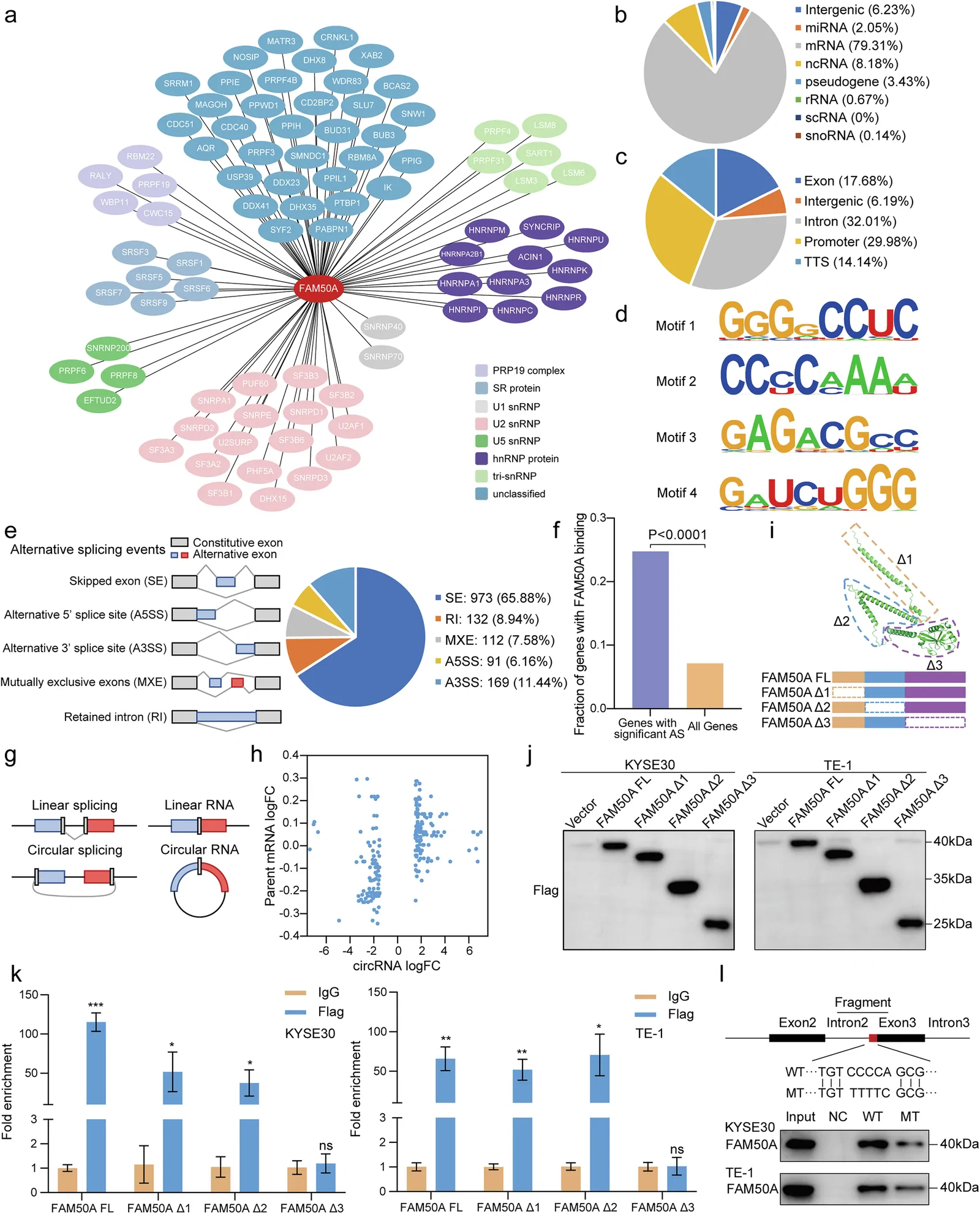
Identifying the transcriptome-wide landscape of FAM50A binding sites and alternative splicing events. **a** The correlation network between FAM50A and other splicing factors was constructed in Cytoscape. Proteins belonging to the spliceosome were classified into the PRP19 complex, SR protein, U1 snRNP, U2 snRNP, U5 snRNP, hnRNP protein, tri-snRNP, and others. **b** The percentages of different RNA molecules that bound to FAM50A. **c** The percentage of the FAM50A–RNA crosslinked regions. **d** Enriched sequence elements of the top-four FAM50A-binding motifs. **e** Schematic diagram of different classes of canonical alternative splicing events. The pie chart shows different classes of FAM50A-dependent canonical alternative splicing events. **f** FAM50A binding is significantly associated with FAM50A-dependent canonical alternative splicing. The fraction of differentially spliced genes with FAM50A binding was compared with that of all genes. **g** Schematic depiction of circRNA formation by the backsplicing of linearly aligned exons. **h** Correlations of the fold changes in the abundance of FAM50A-regulated circRNA (*x*-axis) and its cognate parent mRNA (*y*-axis). **i** Schematic showing full-length (FL) and truncated FAM50A. **j** FL and truncated FAM50A levels were confirmed by western blotting. **k** RIP–qPCR was performed with an anti-Flag antibody to validate the interaction between the FL and truncated FAM50A proteins and CRTC1 RNA. **l** RNA pull-down assays revealed the interaction between the CRTC1 pre-mRNA and the FAM50A protein. *P* values were obtained using the chi-square test (**f**) and two-tailed unpaired Student’s *t* test (**k**), and the results are presented as the means ± SDs. *P < 0.05, **P < 0.01, and ***P < 0.001.

The UniProt database indicated that FAM50A has RNA-binding and RNA-splicing capabilities (Supplementary Fig. S6a). We first used IP–MS to identify proteins that bind to FAM50A in KYSE30 cells and confirmed that FAM50A is a component of the spliceosome. Notably, 85 annotated spliceosome proteins interacted with FAM50A (Supplementary Fig. S6b). Among these proteins, FAM50A predominantly co-immunoprecipitated with U2 snRNP, hnRNP, tri-snRNP, and SR proteins (Fig. 3a). The Gene Ontology (GO) enrichment analysis demonstrated that RNA splicing and pre-mRNA binding were significantly enriched among proteins interacting with FAM50A (Supplementary Fig. S6c). We further verified these interactions by performing endogenous co-immunoprecipitation (Co-IP) and confirmed that FAM50A bound to four core spliceosome proteins (HNRNPL, EFTUD2, HNRNPC, and DDX41) in ESCC cells (Supplementary Fig. S6d). Subsequent immunofluorescence staining revealed that FAM50A colocalized with SC35, a marker of nuclear speckles (a nuclear structure involved in the storage of splicing factors; Supplementary Fig. S6e). The interaction of FAM50A with multiple spliceosome components indicates that FAM50A plays a critical role in the regulation of AS.

We performed a RIP-seq assay of FAM50A in KYSE30 cells to generate a transcriptome-wide map of the interactions between the FAM50A protein and RNA. The results demonstrated that 79.31% of the RNA molecules that bound to FAM50A were mRNAs and that more than 49.69% of the FAM50A–RNA crosslinked regions mapped to exons and introns (Fig. 3b, c). The HOMER algorithm was used to identify the FAM50A-recognizing RNA motif and further characterize the sites of FAM50A that crosslinked with RNA; the most abundant element was the GGGGCCUC 8-mer (Fig. 3d; Supplementary Fig. S6f, g).

An analysis of AS events was conducted on RNA-seq data from FAM50A knockdown and control KYSE30 cells to investigate FAM50A-dependent AS events. Canonical AS events were identified using the rMATS algorithm. Canonical AS event categories, including retained introns, skipped exons, alternative 5’ splice sites, alternative 3’ splice sites, and mutually exclusive exons, were identified. Through the integration of the RNA-binding sites of FAM50A, 1477 FAM50A-dependent canonical AS events were identified (Fig. 3e). The predominant canonical AS event category observed upon FAM50A knockdown was skipped exons (65.88%). Furthermore, FAM50A binding was significantly associated with these changes in canonical AS events, thereby supporting the direct effect of the FAM50A–RNA interaction on AS (Fig. 3f). Given that splicing factors are well documented to mediate both canonical AS and backsplicing, we further explored the regulatory role of FAM50A in circRNA biogenesis in ESCC cells. We investigated the potential involvement of FAM50A in the regulation of backsplicing by profiling the circRNA transcriptome in KYSE30 cells under control and FAM50A knockdown conditions to determine the differential expression of circRNAs. Combined with FAM50A RIP-seq, we identified 137 significantly upregulated and 154 significantly downregulated circRNAs upon FAM50A knockdown, whose parent mRNA expression remained unchanged (Fig. 3g, h).

Furthermore, we selected a FAM50A-dependent canonical AS event for experimental verification: the inclusion of CRTC1 exon 3 upon FAM50A knockdown. When isoform-specific primers were used to discriminate the two transcript isoforms, we found that the proportion of the CRTC1-L transcript containing exon 3 increased, whereas the proportion of the CRTC1-S transcript lacking exon 3 decreased upon FAM50A knockdown (Supplementary Fig. S6h, i). Conversely, the overexpression of FAM50A resulted in the skipping of exon 3, leading to an increase in the proportion of the CRTC1-S transcript (Supplementary Fig. S6i). The AlphaFold 3 algorithm was utilized to conduct a molecular docking analysis of the FAM50A protein and four RNA motifs. The results suggested that the RNA residues predominantly bound to the region near the C-terminus of the FAM50A protein (Supplementary Fig. S6j). Based on this information, we constructed three truncated forms of the FAM50A protein (Fig. 3i, j). The RIP‒qPCR results indicated that when domain 3 (residues 169–339) was deleted, the ability of the FAM50A protein to bind to RNA was lost (Fig. 3k). In addition, an RNA pull-down assay revealed that the FAM50A protein was enriched more abundantly by the wild-type probe than by the mutant probe targeting exon 3 of CRTC1 (Fig. 3l; Supplementary Fig. S6k). These results strongly suggest that FAM50A promotes CRTC1 AS by binding directly to the pre-mRNA. Collectively, these data indicate that FAM50A functions as a regulator of canonical AS by directly binding to pre-mRNAs and is likely involved in the modulation of backsplicing.

### FAM50A is associated with circSDK1 backsplicing by directly binding to the pre-mRNA

**Fig. 4:**
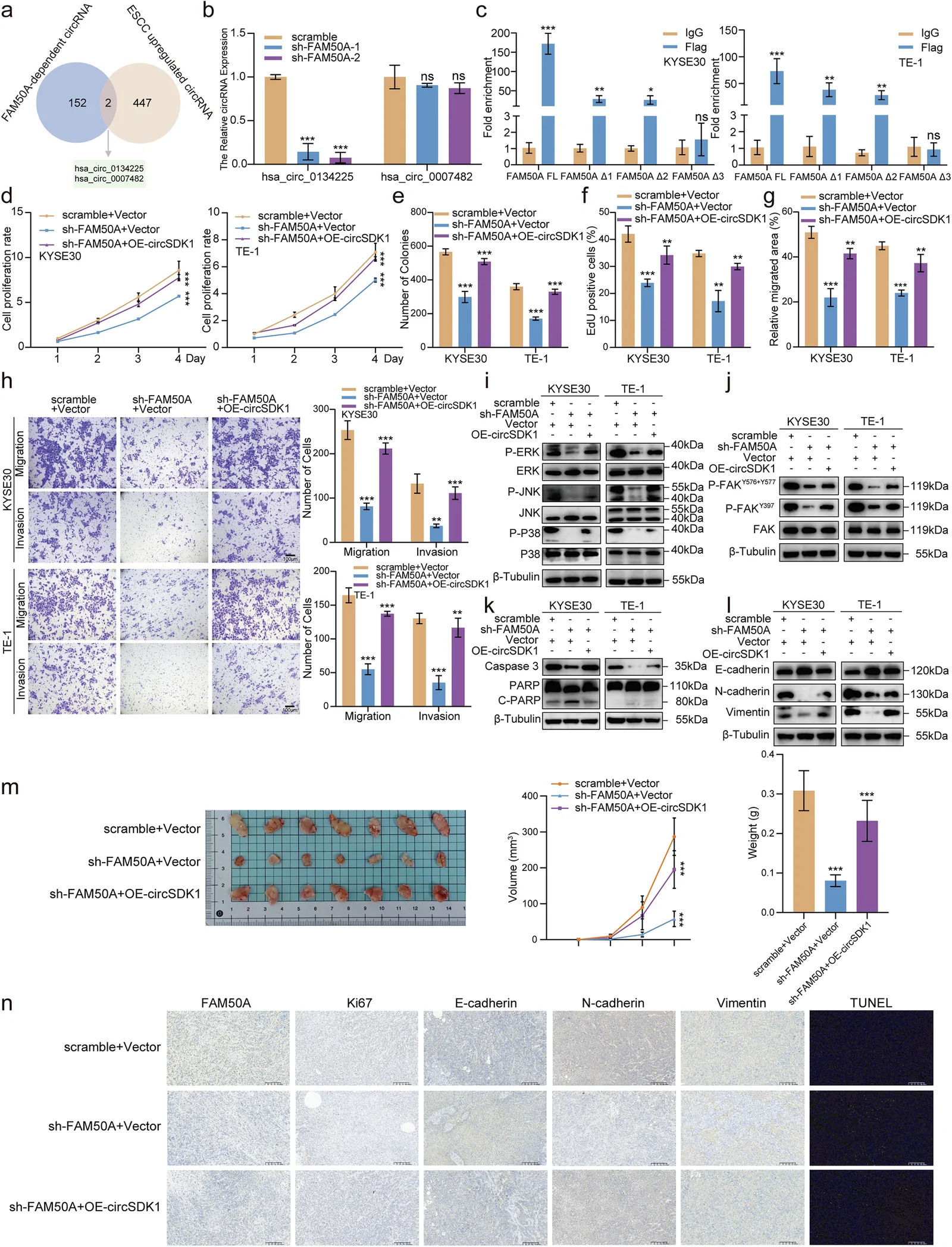
The oncogenic role of FAM50A partially relies on the role of splicing-derived circSDK1 in ESCC. **a** The intersection of FAM50A-dependent circRNAs determined using the analysis described above and upregulated circRNAs detected in four paired ESCC tissues. **b** qPCR confirmation of the two candidate circRNAs in TE-1 cells upon FAM50A knockdown (*n* = 3). **c** RIP‒qPCR was performed with an anti-Flag antibody to validate the interaction between FL and truncated FAM50A protein and the SDK1 pre-mRNA. **d**–**h** Cell proliferation assays (**d**), clonogenic assays (**e**), EdU assays (**f**), wound healing assays (**g**), and Transwell assays (**h**) were performed in ESCC cells to investigate the role of circSDK1 in rescuing the effect of FAM50A knockdown (*n* = 3 for clonogenic assays, EdU assays, wound healing assays, and Transwell assays; *n* = 8 for cell proliferation assays). **i**, **j** The levels of phosphorylated MAPKs (**i**) and FAK (**j**) were detected using western blotting to investigate the role of circSDK1 in rescuing the effects of FAM50A knockdown. **k** Apoptotic markers were detected by western blotting. FAM50A knockdown or circSDK1-overexpressing cells were cultured in suspension. **l** The levels of EMT markers were detected using western blotting. **m** The results from a KYSE30 CDX model showed that circSDK1 could partially rescue the inhibitory effect of FAM50A on tumor growth (*n* = 7). **n** Immunohistochemical staining was conducted to assess the expression levels of Ki67, E-cadherin, N-cadherin, and Vimentin, and a TUNEL assay was conducted to quantify the number of apoptotic cells in the tumors. Scale bars, 200μm. The *P* values were obtained using two-tailed unpaired Student’s *t* test (**b**–**h**, **m**), and the results are presented as the means ± SDs. *P < 0.05, **P < 0.01, ***P < 0.001; ns, not significant.

To date, no reports have described FAM50A-mediated backsplicing events and their role in cancer progression. We therefore performed circRNA-seq on four paired ESCC tissues and adjacent normal tissues. A total of 449 circRNAs were screened and found to be significantly upregulated in ESCC tissues. Following the intersection of the 154 significantly downregulated circRNAs upon FAM50A knockdown, two candidate circRNAs were obtained (Fig. 4a). Using divergent primers, we employed qPCR to detect changes in the expression levels of the two candidate circRNAs following FAM50A knockdown in TE-1 cells. The results demonstrated that the expression level of hsa_circ_0134225 was substantially decreased following FAM50A knockdown in TE-1 cells (Fig. 4b). hsa_circ_0134225 expression decreased expression in FAM50A knockdown cells and increased in cells with FAM50A overexpression (Supplementary Fig. S7a, b). hsa_circ_0134225 is derived from exons 2 to 4 of the sidekick cell adhesion molecule 1 (SDK1) gene and has a length of 415 nt. The qPCR product of circSDK1 was used to validate the backsplicing junction site by performing Sanger sequencing (Supplementary Fig. S7c). As a method to verify the circular form of circSDK1, divergent and convergent primers were used to amplify the circular and linear forms of SDK1, respectively. Agarose gel electrophoresis of the qPCR products revealed that circSDK1 was exclusively amplified from cDNA (Supplementary Fig. S7d). Furthermore, following treatment with actinomycin D and RNase R, circSDK1 was more stable than the linear SDK1 mRNA (Supplementary Fig. S7e, f). Compared with expression obtained using random primers, the relative expression of circSDK1 obtained using the oligo dT primers in the reverse-transcription experiments was significantly lower, while the expression of the linear SDK1 mRNA was not altered (Supplementary Fig. S7g). Moreover, the results of nuclear‒cytoplasmic fractionation indicated that circSDK1 was predominantly located in the cytoplasm (Supplementary Fig. S7h). Taken together, these results indicated that circSDK1 is an upregulated and highly stable circRNA that is localized in the cytoplasm of ESCC cells.

We obtained additional evidence for the direct binding of FAM50A to the SDK1 pre-mRNA by performing RIP‒qPCR assays in ESCC cell lines with an anti-Flag antibody and control IgG. The results of the RIP‒qPCR analysis revealed that FAM50A protein domain 3 exhibited binding affinity for intron 1 of the SDK1 pre-mRNA (Fig. 4c). An RNA pull-down assay revealed that FAM50A was more abundantly enriched by the wild-type probe but not by the mutant probe targeting intron 1 (Supplementary Fig. S7i, j). These findings suggest that FAM50A promotes the formation of circSDK1 by directly binding to the SDK1 pre-mRNA and facilitating its backsplicing.

### The oncogenic function of FAM50A in ESCC partially relies on circSDK1 expression

We investigated whether the regulation of SDK1 backsplicing by FAM50A affects the oncogenic function of FAM50A in ESCC by conducting experiments in cells with a circSDK1 knockdown phenotype to clarify the oncogenic potential of circSDK1 (Supplementary Fig. S7k). We performed growth curve, clonogenic, and EdU assays and found that circSDK1 knockdown significantly reduced the proliferation of ESCC cells (Supplementary Fig. S8a–c). In addition, wound healing and Transwell assays revealed that ESCC cells exhibited limited migration and invasion abilities upon FAM50A knockdown (Supplementary Fig. S8d, e). Furthermore, adhesion assays revealed that circSDK1 knockdown cells exhibited diminished attachment to the ECM (Supplementary Fig. S9a). In addition, we found that circSDK1 knockdown also significantly increased the anoikis rate of ESCC cells in suspension culture using trypan blue exclusion tests of cell viability and caspase-3 activity assays (Supplementary Fig. S9b, c).

We then performed rescue experiments to ascertain whether circSDK1 overexpression could reverse the phenotype induced by FAM50A knockdown. Indeed, circSDK1 overexpression significantly reversed the inhibitory effects of FAM50A knockdown on the proliferation and clonogenic ability of ESCC cells (Fig. 4d–f; Supplementary Fig. S10a, b). In addition, circSDK1 overexpression reversed the reductions in the migration and invasion capacities caused by FAM50A knockdown (Fig. 4g, h; Supplementary Fig. S10c). Furthermore, FAM50A knockdown decreased the adhesion ability, which could be reversed by circSDK1 overexpression (Supplementary Fig. S11a). Additionally, FAM50A knockdown-induced anoikis was effectively attenuated by circSDK1 overexpression (Supplementary Fig. S11b, c). We verified that circSDK1 overexpression could compensate for the reduced MAPKs phosphorylation levels caused by FAM50A knockdown (Fig. 4i). Concurrently, circSDK1 overexpression increased the level of FAK phosphorylation (Fig. 4j). In addition, we demonstrated that circSDK1 increased the ability of ESCC cells in suspension culture to resist anoikis through the detection of apoptosis markers using western blotting (Fig. 4k). CircSDK1 overexpression promoted the EMT in ESCC cells (Fig. 4l). Moreover, circSDK1 overexpression reversed the inhibition of CDX growth induced by FAM50A knockdown (Fig. 4m). Immunohistochemistry and TUNEL assays revealed that circSDK1 overexpression could increase the Ki67 index, promote the EMT, and prevent apoptosis, thus rescuing the function of FMA50A knockdown cells in vivo (Fig. 4n; Supplementary Fig. S12a–f). Furthermore, circSDK1 knockdown markedly reversed the increased proliferation, migration, and invasion of ESCC cells induced by FAM50A overexpression, confirming that circSDK1 is essential for the tumor-promoting activity of FAM50A (Supplementary Fig. S13a–e). Additionally, isoform-specific rescue assays were conducted to distinguish the roles of the circular and linear SDK1 transcripts. Only the ectopic expression of circSDK1, but not linear SDK1, rescued MAPKs and FAK phosphorylation in SDK1/circSDK1 co-knockdown cells, confirming that circSDK1 is the functional isoform responsible for FAM50A-mediated activation of the MAPK and FAK pathways (Supplementary Fig. S14a–c).

### CircSDK1 increases MAPKs and FAK phosphorylation by occupying the phosphatase domain of DUSP22

**Fig. 5:**
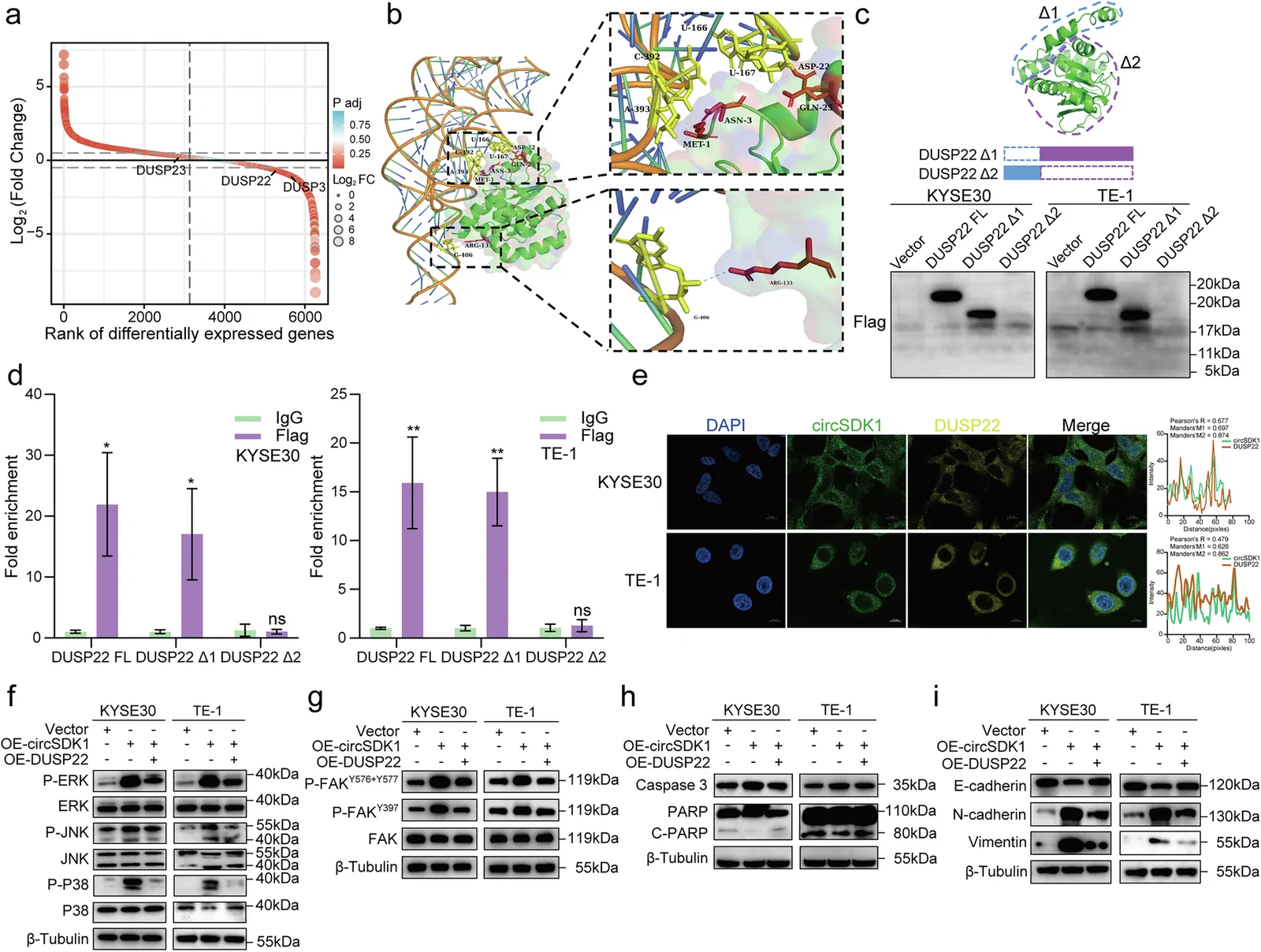
CircSDK1 increases MAPKs and FAK phosphorylation by occupying the phosphatase domain of DUSP22. **a** The protein expression ranking of dual-specificity phosphatases in Changhai cohort I. **b** Molecular docking of circSDK1 and DUSP22 was performed using AlphaFold 3. **c** Schematic showing the FL and truncated DUSP22 proteins, as confirmed by western blotting. **d** RIP‒qPCR was performed with an anti-Flag antibody to validate the interaction between the FL and truncated DUSP22 proteins. **e** Immunofluorescence staining showed the co-localization of DUSP22 (yellow) with circSDK1 (green) in KYSE30 and TE-1 cells. Scale bar, 10 μm. The quantification and analysis of co-localization were performed with Coloc 2 and Plot Profile (KYSE30, Pearson’s *R* = 0.677, Manders’ M1 = 0.697, Manders’ M2 = 0.874; TE-1, Pearson’s *R* = 0.479, Manders’ M1 = 0.626, Manders’ M2 = 0.862). **f,** **g** The levels of phosphorylated MAPKs (**f**) and FAK (**g**) were detected using western blotting to investigate whether DUSP22 overexpression can reverse the effect of circSDK1. **h** The levels of apoptotic markers were detected using western blotting. DUSP22-overexpressing or circSDK1-overexpressing cells were cultured in suspension. **i** The levels of EMT markers were detected using western blotting to investigate whether DUSP22 overexpression can reverse the effect of circSDK1. The *P* value was obtained using a two-tailed unpaired Student’s *t* test (**d**), and the results are presented as the means ± SDs. *P < 0.05, **P < 0.01, ***P < 0.001; ns, not significant.

We first examined the protein-coding potential of circSDK1to explore how it leads to the phosphorylation of MAPKs and FAK and to tumor progression. We found that circSDK1 exhibited minimal protein-coding potential, which was attributable to the absence of an open reading frame or internal ribosome entry site in the TRANSCIRC and circBank databases (Supplementary Fig. S15a, b). Next, we conducted RIP–qPCR on KYSE30 and TE-1 cells. The results revealed no significant difference in the fold enrichment of circSDK1 between the IgG group and the AGO2 group, suggesting that circSDK1 was unlikely to act as an miRNA sponge (Supplementary Fig. S15c). Additionally, circSDK1 was primarily detected in the cytoplasm (Supplementary Fig. S7h). Therefore, we hypothesized that circSDK1 might interact with proteins. Certain dual-specificity phosphatases (DUSPs) can specifically dephosphorylate MAPKs and FAK. We found that DUSP22 and DUSP3 may act as tumor suppressors, given that their expression levels were significantly lower in ESCC tissues (Fig. 5a). Studies have demonstrated that DUSP22 is capable of dephosphorylating both MAPKs and FAK. Therefore, we selected DUSP22 for further investigation. We first used AlphaFold 3 to predict the molecular docking of circSDK1 and DUSP22 and observed that circSDK1 binds to the phosphatase domain of DUSP22 (Fig. 5b). The RIP‒qPCR analysis using the truncated DUSP22 protein revealed that domain 2 (the phosphatase domain) was the main fragment responsible for binding to circSDK1 (Fig. 5c, d). Guided by the AlphaFold 3 predictions, we constructed a DUSP22 point mutant with alanine substitutions at five putative hydrogen bond-forming residues (M1A/N3A/D22A/Q25A/R133A). The RIP‒qPCR analysis demonstrated that this mutant could not effectively enrich circSDK1, confirming that these interface residues mediate the direct interaction between DUSP22 and circSDK1 (Supplementary Fig. S15d). Electrophoretic mobility shift assays (EMSAs) with the purified recombinant DUSP22 protein confirmed the direct and specific binding of circSDK1 to DUSP22 in vitro. A protein‒probe complex was formed with the wild-type circSDK1 probe but not with the mutant probe; this binding was partially attenuated by 10× excess cold competitor and fully abolished at 50× and 100× excess (Supplementary Fig. S15e). In addition, immunofluorescence staining revealed the significant colocalization of circSDK1 and DUSP22, with both molecules being located primarily in the cytoplasm (Fig. 5e). The results of the western blot analysis demonstrated that DUSP22 overexpression could reduce the increase in the levels of phosphorylated MAPKs and FAK caused by circSDK1 overexpression (Fig. 5f, g). In vitro phosphatase activity assays using purified recombinant proteins further confirmed that circSDK1 directly inhibited DUSP22 catalytic activity in a dose-dependent manner, with increasing circSDK1 probe concentrations leading to progressively reduced phosphatase activity (Supplementary Fig. S15f, g). Furthermore, DUSP22 overexpression reversed circSDK1 overexpression-induced anoikis resistance in ESCC cell lines (Fig. 5h). Additionally, the induction of the EMT by circSDK1 overexpression was inhibited by DUSP22 overexpression (Fig. 5i). Moreover, the pharmacological inhibition of MAPKs with (E)-Osmundacetone or FAK with Y15 markedly attenuated the malignant phenotypes of ESCC cells, and treatment with both inhibitors resulted in an even stronger suppressive effect, providing evidence that MAPKs/FAK activation drives ESCC progression (Supplementary Fig. S16a–e). In summary, these data indicate that circSDK1 abnormally increases MAPKs and FAK phosphorylation by binding to DUSP22 and inhibiting its dephosphorylation function.

### The transcription factor TFAP2C contributes to FAM50A transcription, resulting in its aberrant upregulation

**Fig. 6:**
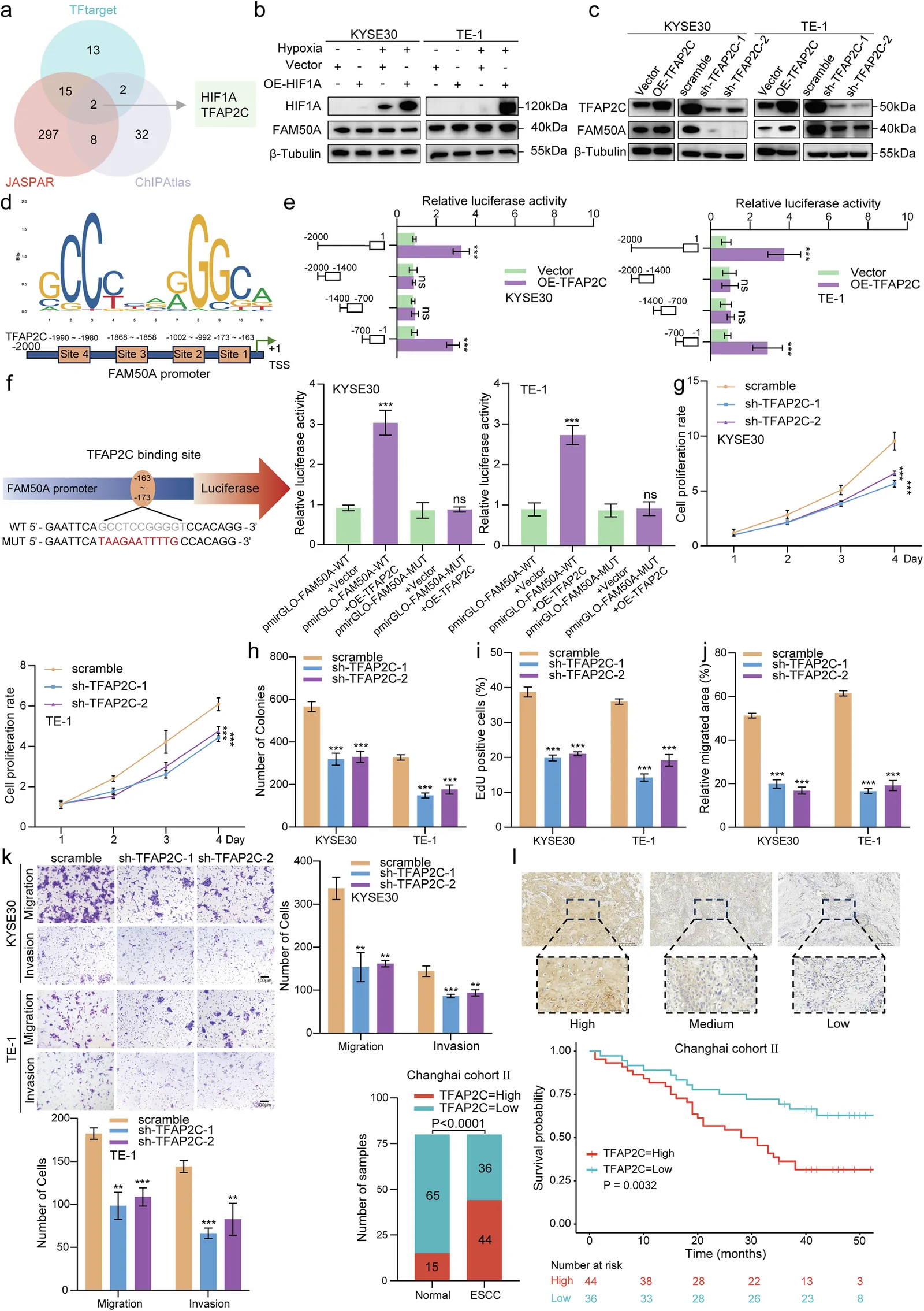
The transcription factor TFAP2C contributes to FAM50A transcription, resulting in its aberrant upregulation. **a** Venn diagram showing the transcription factors that bind to the promoter region of FAM50A identified in the TFtarget, JASPAR, and ChIPAtlas databases. **b** HIF1A-overexpressing or control cells were cultured under normoxic (20% O_2_) or hypoxic (1% O_2_) conditions, and HIF1A and FAM50A expression were detected using western blotting. **c** TFAP2C and FAM50A expression were detected in TFAP2C-overexpressing or knockdown cells using western blotting. **d** Schematic of the putative TFAP2C binding motif and the predicted TFAP2C binding sites in the FAM50A promoter determined using JASPAR. **e** Cells were transfected with the full-length FAM50A promoter or one of three truncation mutants, and luciferase activity was analyzed after transfection. **f** The cells were transfected with the FAM50A-WT or FAM50A-MUT promoter vector, and luciferase activity was analyzed after transfection. **g**–**k** Cell proliferation assays (**g**), clonogenic assays (**h**), EdU assays (**i**), wound healing assays (**j**), and Transwell assays (**k**) were performed using ESCC cells with TFAP2C knockdown and the results were compared with the corresponding control cells (*n* = 3 for the clonogenic assay, EdU assay, wound healing assay and Transwell assay, and *n* = 6 for the cell proliferation assay). **l** Representative images of immunohistochemical staining showing high, medium, and low TFAP2C expression in Changhai cohort II. Scale bars, 200 μm. Statistical analysis of TFAP2C expression based on immunohistochemical staining of sections from patients in Changhai cohort II. Kaplan–Meier analysis of the correlation between FAM50A expression and the overall survival of patients in Changhai cohort II. The *P* values were obtained using two-tailed unpaired Student’s *t* tests (**e**–**k**), the chi-square test (**l**), and log-rank test (**l**). The optimal cut-off value in the Kaplan‒Meier analysis was determined using the minimum *P* value method. The data are presented as the means ± SDs, unless stated otherwise. *P < 0.05, **P < 0.01, ***P < 0.001; ns, not significant.

Using the TFtarget, JASPAR, and ChIPAtlas databases, we identified potential upstream transcription factors associated with FAM50A upregulation in ESCC (Fig. 6a). Hypoxia-inducible factor 1 subunit alpha (HIF1A) overexpression under normoxic or hypoxic conditions did not alter FAM50A expression levels (Fig. 6b). In contrast, the expression level of FAM50A was altered by TFAP2C overexpression or knockdown (Fig. 6c). Therefore, TFAP2C was selected for further study. The DNA-binding motif of TFAP2C and the potential binding sites in the FAM50A promoter region were obtained from the JASPAR database (Fig. 6d). We constructed a series of luciferase vectors containing different truncations of the FAM50A promoter to elucidate TFAP2C-mediated FAM50A transcription. The luciferase reporter assay revealed that the promoter region from -1 to -700 bp in the FAM50A gene contained a TFAP2C-responsive site (Fig. 6e). Consequently, we constructed wild-type (FAM50A-WT) and mutant (FAM50A-MUT) promoter luciferase reporter vectors by mutating the FAM50A promoter sequence (-163 to -173 bp) that was predicted to be bound by TFAP2C (Fig. 6f). TFAP2C overexpression increased the relative luciferase activity of the FAM50A-WT vector but only minimally affected the luciferase activity of the FAM50A-MUT vector (Fig. 6f). Furthermore, the chromatin immunoprecipitation–qPCR (ChIP‒qPCR) analysis demonstrated that TFAP2C exhibited a significant binding affinity for the FAM50A promoter region sequence near site 1 (Supplementary Fig. S17a). These results indicated that TFAP2C contributes to FAM50A expression in ESCC by binding to the FAM50A promoter region.

Furthermore, we elucidated the oncogenic potential of TFAP2C in ESCC by performing a series of experiments to assess the phenotype. TFAP2C knockdown significantly reduced the proliferation and clonogenic ability of ESCC cell lines, as determined by the results of the growth curve analysis, clonogenic assays, and EdU assays (Fig. 6g–l; Supplementary Fig. S17b, c). Additionally, the results of the wound healing and Transwell assays indicated that TFAP2C knockdown significantly weakened the migration and invasion abilities of ESCC cells (Fig. 6j, k; Supplementary Fig. S17d). Conversely, TFAP2C overexpression promoted the proliferation, clonogenic activity, migration, and invasion of ESCC cells (Supplementary Fig. S18a–e). Moreover, we discovered that FAM50A overexpression reversed the decreases in proliferation, clonogenic activity, migration, and invasion induced by TFAP2C knockdown (Supplementary Fig. S19a–e). Consequently, TFAP2C could act as an upstream transcription factor for FAM50A that promoted its oncogenic activity in ESCC.

An analysis of TCGA-EC and GSE53624 cohorts revealed the significant upregulation of TFAP2C transcript levels in ESCC (Supplementary Fig. S20a, b). In addition, we found that TFAP2C protein levels were upregulated in ESCC in the Changhai cohort II and Liu et al. cohort (Fig. 6l; Supplementary Fig. S20c). According to the Kaplan‒Meier survival curves, a significant difference in the prognosis was not observed between patients with high or low TFAP2C expression in TCGA-ESCC cohort (Supplementary Fig. S20d). However, a significant difference in survival was observed in TCGA-EAC cohort (Supplementary Fig. S20e). Additionally, the prognosis of ESCC patients with high TFAP2C expression in the Changhai cohort II, GSE53624 cohort, and Liu et al. cohort was poor (Fig. 6l; Supplementary Fig. S20f, g). Furthermore, a correlation analysis revealed a positive correlation between FAM50A expression and TFAP2C expression in the GSE53624 cohort, Liu et al. cohort, and Changhai cohort II (Supplementary Fig. S20h–j).

### The inhibition of FAM50A with rifabutin increases the efficacy of cisplatin in ESCC

**Fig. 7:**
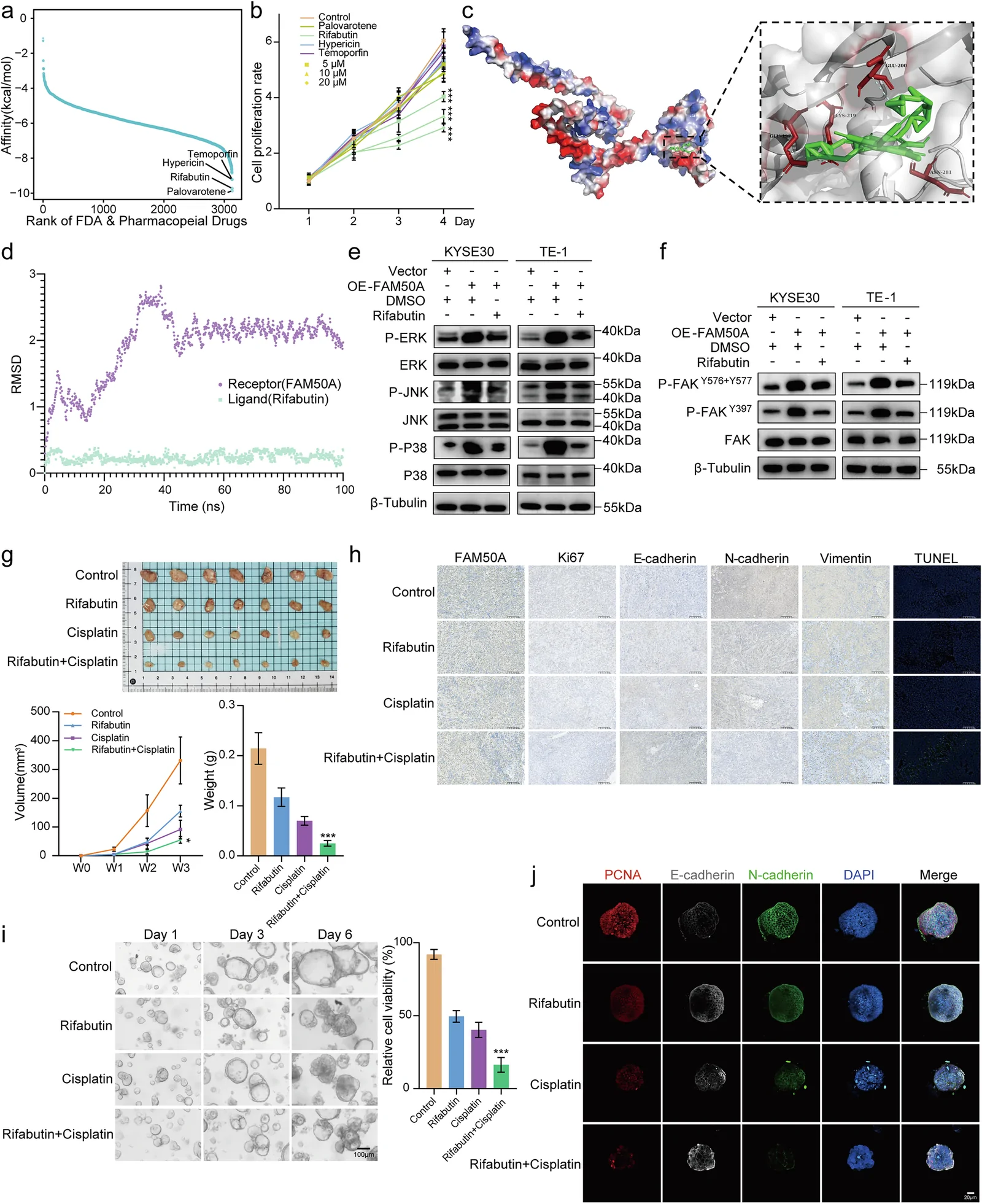
The inhibition of FAM50A with rifabutin increases the efficacy of cisplatin in ESCC. **a** Ranking of the binding affinity of small molecules for FAM50A according to the virtual screen. **b** A cell proliferation assay was performed in TE-1 cells using the four candidate inhibitors (*n* = 6). **c** Molecular docking of rifabutin and FAM50A was performed using AutoDock Vina. **d** The backbone RMSD of the interaction between the receptor (FAM50A) and ligand (rifabutin) during the molecular dynamics simulation. **e**, **f** The levels of phosphorylated MAPKs (**e**) and FAK (**f**) were detected using western blotting to investigate the effect of rifabutin. **g** KYSE30 CDXs were dissected from nude mice (*n* = 7). The growth curves of the tumor volumes were calculated for the CDX models (*n* = 7). The weights of CDXs dissected from nude mice are shown (*n* = 7). **h** Immunohistochemical staining was conducted to assess the expression levels of Ki67, E-cadherin, N-cadherin, and Vimentin, and a TUNEL assay was conducted to quantify the number of apoptotic cells in the tumors. **i** Bright-field images of ESCC PDOs treated with rifabutin, cisplatin, or their combination. **j** Immunofluorescence staining was conducted to assess the expression levels of PCNA (red), E-cadherin (gray), and N-cadherin (green) in ESCC PDOs. The *P* values were obtained using two-tailed unpaired Student’s *t* test (**b**, **g**, **i**), and the results are presented as the means ± SDs. *P < 0.05, **P < 0.01, and ***P < 0.001.

As FAM50A plays an important role in tumor progression, we sought to identify inhibitors of FAM50A to use as potential therapies for ESCC. By comparing the crystal structure and AlphaFold-predicted structure of FAM50A, we identified a potential binding pocket for FAM50A and constructed a docking box (Supplementary Fig. S21a–c). By employing a virtual screen based on the AutoDock Vina algorithm, the putative FAM50A inhibitors palovarotene, rifabutin, hypericin, and temoporfin were identified as having the highest binding affinity for FAM50A from a library of 3158 FDA-approved and pharmacopeial drugs (Fig. 7a). We evaluated the effects of these four candidate inhibitors on the proliferation of ESCC cells by constructing growth curves and found that rifabutin had the most significant inhibitory effect (Fig. 7b; Supplementary Fig. S21d). The results of molecular docking showed that rifabutin interacts with the FAM50A residues Glu197, Glu200, Lys219, and Asn281 (Fig. 7c). The best conformation of the FAM50A–rifabutin complex, as determined by the docking analysis, was designated as the initial conformation for the molecular dynamics simulation. The results indicated that the backbone root-mean-square deviation (RMSD) of the receptor (FAM50A) and ligand (rifabutin) tended to stabilize throughout the simulation, indicating the high stability of the FAM50A–rifabutin complex (Fig. 7d). The number of hydrogen bonds (H-bonds) formed between FAM50A and rifabutin remained stable throughout the simulation, thereby ensuring the stability of the complex (Supplementary Fig. S21e). The analysis of the root-mean-square fluctuation (RMSF) of each residue of FAM50A demonstrated that the RMSF of the residues bound to rifabutin was low (Supplementary Fig. S21f). These findings thus indicated that the rifabutin-binding region was highly stable throughout the simulation. We observed a small and stable radius of gyration (RoG) for the FAM50A–rifabutin complex, indicating conformational compaction (Supplementary Fig. S21g). The qPCR analysis revealed that rifabutin inhibited FAM50A-dependent AS events, including canonical AS and backsplicing events (Supplementary Fig. S21h, i). Western blot analysis revealed that rifabutin could reduce the increase in the levels of phosphorylated MAPKs and FAK in FAM50A-overexpressing cells (Fig. 7e, f). Rifabutin reversed the FAM50A overexpression-induced anoikis resistance in ESCC cells (Supplementary Fig. S21j). Furthermore, the induction of the EMT by FAM50A overexpression could be inhibited by rifabutin (Supplementary Fig. S21k). In conclusion, rifabutin can bind to FAM50A, thereby impeding its oncogenic activity.

CDX models demonstrated that rifabutin can inhibit tumor growth. Furthermore, when rifabutin was combined with cisplatin, even greater inhibition of tumor growth was observed (Fig. 7g). The results of the immunohistochemical staining and TUNEL assays also showed that the combination of rifabutin and cisplatin further decreased the Ki67 index, inhibited the EMT, and promoted apoptosis (Fig. 7h; Supplementary Fig. S21l–q). The combination of rifabutin and cisplatin significantly inhibited the growth of ESCC patient-derived organoids (PDOs) (Fig. 7i). Immunofluorescence staining of PDOs revealed that the combination of rifabutin and cisplatin further inhibited proliferating cell nuclear antigen (PCNA) activity and the EMT (Fig. 7j). Additionally, an ESCC patient-derived xenograft (PDX) model showed that the combination of rifabutin and cisplatin resulted in more significant growth inhibition (Supplementary Fig. S21r). Consequently, the combination of rifabutin with cisplatin may result in a synergistic effect, thereby increasing the sensitivity of ESCC to chemotherapeutic drugs.

## Discussion

Dysregulated expression of splicing factors and AS reprogramming are pivotal factors in the process of oncogenesis. We screened survival-related splicing factors in ESCC using multi-omics and multi-cohort data. We found that FAM50A expression was frequently upregulated in ESCC and that high levels of FAM50A were associated with a poor prognosis. We then investigated the functional role of FAM50A and found that FAM50A promoted the proliferation and migration of ESCC cells. The gene set enrichment analysis revealed that FAM50A was closely related to the MAPK signaling pathway, focal adhesion, and cell‒cell junctions. We confirmed that FAM50A can activate MAPKs and FAK. Furthermore, FAM50A promoted the EMT, enhanced ECM adhesion, and led to the acquisition of anoikis resistance in ESCC cells. These results indicated that FAM50A might potentiate the entire process of metastasis in ESCC cells. FAM50A can disrupt cell–cell junctions by promoting EMT in ESCC cells, thereby initiating metastasis. Subsequently, FAM50A can increase anoikis resistance in ESCC cells, thus resulting in increased cell viability during dissemination. Ultimately, FAM50A-induced ECM adhesion has the potential to increase the ability of ESCC cells to colonize metastatic foci. In conclusion, these findings indicate that FAM50A has oncogenic properties and is associated with an unfavorable prognosis for patients with ESCC.

FAM50A is essential for the assembly and catalytic activity of the spliceosome^23^. Nonetheless, FAM50A-regulated cancer-specific AS events and its binding motifs remain to be extensively elucidated. We performed RIP-seq to map transcriptome-wide FAM50A–RNA interactions and found that FAM50A binding sites were highly enriched in exons and introns. We also identified four FAM50A-binding RNA motifs using the HOMER algorithm. We further revealed that FAM50A knockdown results in extensive canonical AS and backsplicing events. A comprehensive analysis of the RIP-seq and RNA-seq data revealed that a downstream backsplicing event (circSDK1) may be involved in FAM50A-mediated ESCC progression. These findings revealed that FAM50A extensively regulates AS by binding directly to its RNA targets.

Accumulating evidence has indicated that dysregulated circRNAs may play pivotal roles in tumor progression^10^. In this study, we identified that circSDK1, which is derived from the backsplicing of SDK1 and is regulated by FAM50A, plays a crucial role in ESCC progression. CircRNAs have complex and diverse functions. The most extensively debated mechanism is the competing endogenous RNA hypothesis, yet this hypothesis remains controversial^12,30–32^. A thorough investigation of a substantial number of annotated circRNAs revealed that the number of miRNA binding sites in the majority of circRNAs was not as substantial as anticipated. In certain models, the number of target sites required for a circRNA to compete with an miRNA may have been overestimated. As described in previous mechanistic studies, circRNAs regulate protein function through interactions with RNA-binding proteins^30^. In addition, recent studies have shown that specific circRNAs can encode polypeptides^33^. We found that circSDK1 is unlikely to act as an miRNA sponge and has translational potential. We showed that circSDK1 can bind to the phosphatase domain of DUSP22, thereby inhibiting its dephosphorylation function. Studies have shown that DUSP22 is capable of dephosphorylating both MAPKs and FAK^34–36^. Consequently, circSDK1 can activate MAPKs and FAK by inhibiting the dephosphorylation function of DUSP22. DUSP22 is expressed in a variety of tissues and cells and thus contributes to the regulation of numerous pathophysiological processes, including cell motility, tumor progression, and inflammation^37,38^. Ge et al. demonstrated that DUSP22 is a promising suppressor of non-alcoholic steatohepatitis and hepatocellular carcinoma and has the capacity to inhibit tumor development^34^. In this study, the expression of DUSP22 was downregulated in ESCC, and it exerted a potential suppressive effect. Concurrently, circSDK1 impeded the suppressive function of DUSP22 by occupying its phosphatase domain. DUSP22 overexpression can restore its dephosphorylation and suppressive functions, suggesting the presence of a competitive binding interaction between circSDK1 and the substrates of DUSP22. Consequently, in the present study, we elucidated the detailed mechanism of the novel oncogenic splicing factor FAM50A, identified a splicing factor-dependent circRNA, circSDK1, and further elucidated the mechanism underlying the function of circSDK1.

The upregulation of FAM50A expression may be due to altered transcriptional regulation^39^. We aimed to elucidate the mechanism underlying FAM50A upregulation in ESCC by identifying transcription factors that positively regulate FAM50A expression. Our results showed that TFAP2C activates FAM50A transcription and that TFAP2C is a vital transcription factor involved in tumor progression. Zhang et al. found that the upregulation of TFAP2C is responsible for the malignancy of gastric squamous cell carcinoma and resistance to chemotherapy^40^. The results of this study revealed that genes whose expression was downregulated by TFAP2C knockout were enriched in several signaling pathways regulating kinase activity, such as the MAPK signaling pathway, the cyclin-dependent kinase (CDK) signaling pathway, and the Janus tyrosine kinase (JAK)–signal transducer and activator of transcription (STAT) signaling pathway. Another study showed that TFAP2C drives cisplatin resistance in bladder cancer by upregulating the expression of yes-associated protein (YAP) and activating the β-catenin signaling pathway^41^. Thus, we identified the upstream transcription factor of FAM50A, which could partially explain its oncogenic mechanism.

AS clearly plays a critical role in tumorigenesis; therefore, cancer therapies targeting AS have broad application prospects^42–45^. A variety of methods are currently in preclinical and clinical development stages, ranging from the inhibition of key splicing factors to the regulation of specific AS events. Studies have shown that several aryl sulfonamides can regulate the degradation of RNA-binding motif protein 39 (RBM39), thereby exhibiting anti-cancer properties^46–48^. RNA-based therapeutic approaches permit the regulation of pre-mRNA AS with specificity for virtually any sequence. The BCL-x gene can produce the pro-apoptotic BCL-xS isoform or the anti-apoptotic BCL-xL isoform through AS. Antisense oligonucleotides that promote BCL-xS formation can induce apoptosis in glioblastoma cell lines^49,50^. FAM50A has emerged as a promising target for cancer therapy. FAM50A causes widespread changes in AS patterns, which thereby confer its oncogenic potential. Therefore, we decided to screen for inhibitors that target FAM50A. We screened a library of FDA-approved and pharmacopeial drugs and discovered that rifabutin inhibits FAM50A activity. Lee et al. verified that rifabutin can increase the cytotoxicity of antimitotic drugs in resistant cancer cells through strong P-glycoprotein inhibitory activity^51^. Patil et al. found that rifabutin can inhibit the interaction between the programmed death receptor 1 (PD-1) and programmed cell death ligand (PD-L1) proteins, thereby exerting anti-cancer activity^52^. In addition, studies have shown that rifabutin exhibits anti-*Helicobacter pylori* activity, thereby helping to prevent the onset of gastric cancer^53,54^. In this study, we ascertained the anti-cancer activity of rifabutin, an inhibitor of FAM50A, in CDX, PDO, and PDX models.

This study has certain limitations. First, FAM50A, an essential component of the spliceosome, can induce a wide range of canonical AS and backsplicing events. In this study, we only validated one oncogenic target. Whether other targets mediate the oncogenic activity of FAM50A requires further research. However, the present work focused on FAM50A as the core target for inhibitor development and screening, which, to some extent, addresses this limitation. Second, the intricate regulatory relationships among MAPKs, FAK, anoikis resistance, and the EMT require further clarification. FAK can act as an upstream signal of extracellular regulated protein kinase (ERK1/2), activating ERK1/2 activity in pancreatic ductal adenocarcinoma^55^. Another study revealed that ERK1/2 can increase anoikis resistance in hepatocellular carcinoma^56^. In this study, we found that MAPKs and FAK can act as common substrates of DUSP22 and can be dephosphorylated and inactivated by DUSP22. Therefore, further studies are needed to clarify the complex regulatory mechanisms involved.

**Fig. 8:**
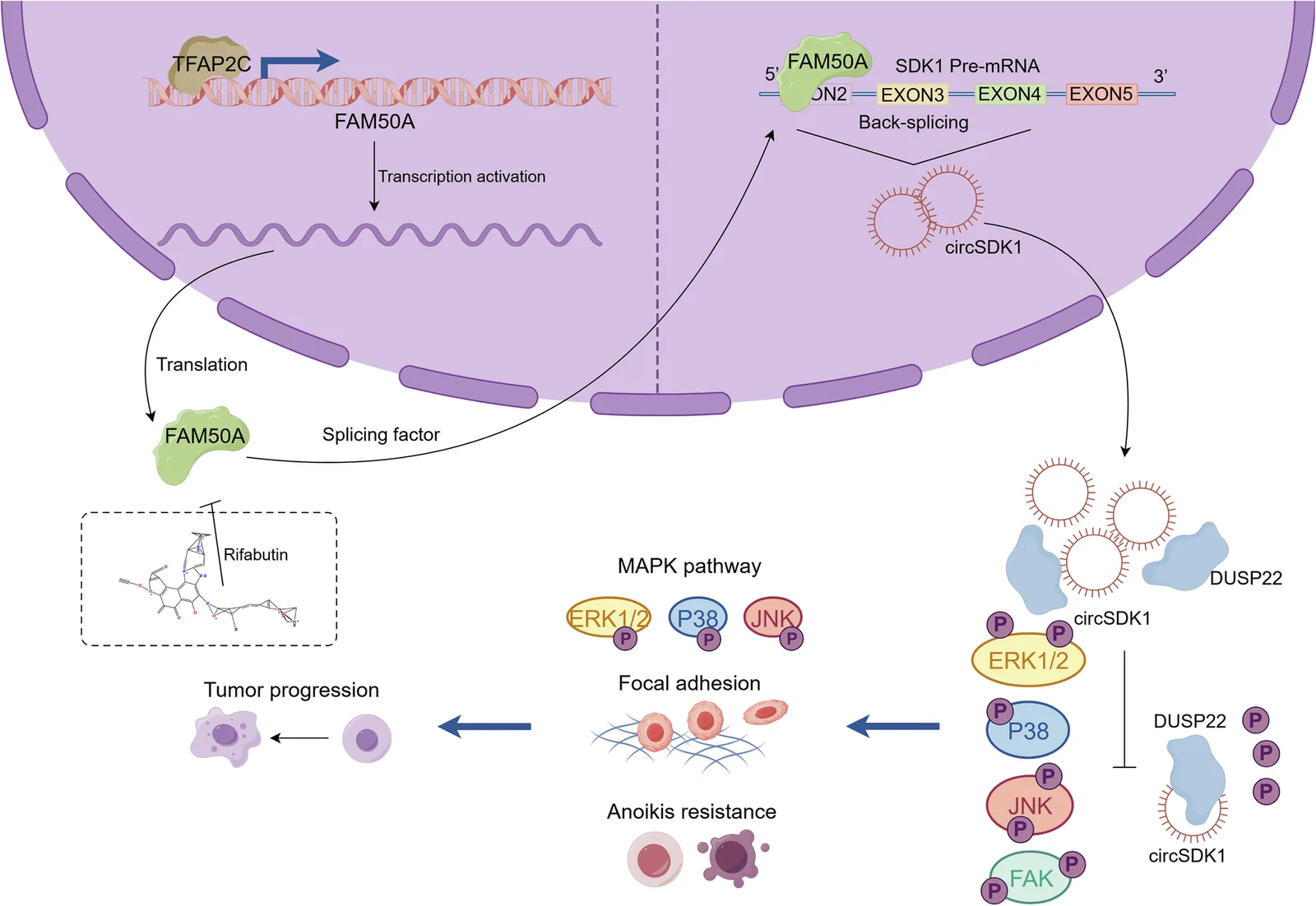
Schematic diagram showing that the splicing factor FAM50A promotes backsplicing of the SDK1 pre-mRNA to form circSDK1, leading to the activation of MAPKs and FAK. The transcription factor TFAP2C can activate FAM50A transcription. Upregulated FAM50A expression promotes increased circSDK1 formation. CircSDK1 can bind to DUSP22, occupying the phosphatase domain of DUSP22 and thereby inhibiting its dephosphorylation function. Consequently, the levels of phosphorylated MAPKs and FAK increase, and they remain aberrantly activated. Rifabutin can act as a small-molecule inhibitor of FAM50A that inhibits its biological function.

In summary, this study demonstrated that FAM50A functions as a novel oncogenic splicing factor and a prognostic marker in ESCC. We further revealed the binding motifs of FAM50A and its transcriptome-wide binding patterns. We identified FAM50A-dependent canonical AS and backsplicing events. Additionally, as shown in Fig. 8, we discovered that FAM50A exerts oncogenic effects via the FAM50A/circSDK1/DUSP22 axis. We found that TFAP2C can activate the transcription of FAM50A, resulting in its aberrant upregulation. Finally, the inhibition of FAM50A by rifabutin may represent a viable therapeutic approach for ESCC.

## Materials and methods

### Human tissue samples

Patient samples, including 108 pairs of ESCC and adjacent tissues, were obtained from Changhai Hospital between 2021 and 2023. Of these tissues, metastatic lymph node tissues that matched 24 paired samples were obtained for mass spectrometry-based protein identification. Eighty paired samples were used to construct a tissue microarray. Four paired samples were selected for circRNA-seq.

### Cells and cell culture

Eight human ESCC cell lines (KYSE510, KYSE450, KYSE410, KYSE180, KYSE150, KYSE30, Eca109, and TE-1), a human EAC cell line (BE-3), and a human immortalized normal esophageal epithelial cell line (HET-1A) were used in this study. KYSE510, KYSE410, KYSE180, KYSE30, Eca109, and TE-1 cells were purchased from Procell Life Science & Technology (Wuhan, China); KYSE450 cells were purchased from FuHeng Biology (Shanghai, China); KYSE150 cells were purchased from the Chinese Academy of Sciences (Shanghai, China); BE-3 cells were purchased from the American Type Culture Collection; and HET-1A cells were purchased from JsCall Biology (Shanghai, China). All cells were cultured in RPMI 1640 (Gibco) supplemented with 10% fetal bovine serum (FBS; Gibco) and 1% penicillin‒streptomycin (Gibco) at 37 °C in a humidified incubator with 5% CO_2_. All the cell lines underwent short tandem repeat identification and were confirmed to be free of mycoplasma contamination.

### Immunohistochemical staining

Immunohistochemistry was performed on tissue microarrays (TMA) and xenograft tumor sections with distinct quantification protocols. For the TMA samples, staining was independently scored by two blinded pathologists. The final score (range of 0–12 points) was calculated as the product of the staining intensity (0 = negative, 1 = weak, 2 = moderate, 3 = strong) and proportion of positive cells (0 = < 5%, 1 = 5%–25%, 2 = 25%–50%, 3 = 50%–75%, 4 = > 75%). Samples with scores ≥ 7 were defined as having high expression, and those with scores < 7 were defined as having low expression. Staining in xenograft sections was quantified using ImageJ (version 1.54 f). After color deconvolution to separate the hematoxylin and DAB channels, the total cell number was counted using watershed-assisted nucleus segmentation, and the total integrated optical density (IOD) was measured using a uniform threshold. The IOD per cell was calculated to normalize for variations in the cell density.

### siRNA, plasmid, and lentivirus

Cells (6 × 10⁵/well) were seeded into 6-well plates. Following a 24-hour culture period, during which the cell density reached approximately 80%, RNA interference and plasmid transfection were performed. In the RNA interference experiment, 25 pmol of siRNA was mixed with 7.5 μL of Lipofectamine RNAiMAX in Opti-MEM (Gibco), and the mixture was then incubated at room temperature for 5 minutes before being added to the cells. For plasmid transfection, 2 μg of plasmid was mixed with 7.5 μL of Lipofectamine 3000 and 5 μL of P3000 in Opti-MEM, incubated at room temperature for 20 min, and then added to the cells. Following a 48-h culture period, the infected cells were collected. ESCC cell lines (KYSE30 and TE-1) were infected with a lentivirus for a period of 24 h. The selection of stably transfected cell lines was achieved through the incorporation of puromycin (2.5 μg/mL; Beyotime) into the culture medium, followed by a seven-day culture period.

### RNA isolation and real-time quantitative PCR (qPCR)

Total RNA was extracted using TRIzol reagent (Invitrogen). Subsequently, 1 μg of RNA was reverse transcribed into cDNA using an Evo M-MLV RT Kit (Accurate Biotechnology). qPCR was performed using a SYBR Green Premix Pro Taq HS qPCR Kit (Accurate Biotechnology) on a LightCycler 480 II system (Roche). The primer sequences are summarized in Supplementary Table S1.

### Western blot

Proteins were extracted using RIPA lysis buffer (TargetMol), and separated using SDS‒PAGE. Following the transfer of the SDS‒PAGE gel to a PVDF membrane (Millipore), the blocking process was initiated using 5% skim milk (Beyotime) at 37 °C for 60 min. The PVDF membrane was subjected to an overnight incubation with the primary antibody at 4 °C, followed by an incubation with the corresponding HRP-labeled secondary antibody.

### Cell proliferation assay

The cell proliferation assay was conducted using 96-well plates. A 100 μL cell suspension containing 3000 cells was added to each well, and the cells were cultured at 37 °C with 5% CO₂ for 24 h, 48 h, 72 h, and 96 h, respectively. At each designated time point, 10 μL of Cell Counting Kit-8 (TargetMol) solution was added, and the cells were then incubated in the incubator for 2 h. Subsequently, the absorbance at 450 nm was measured using a SpectraMax i3 (Molecular Devices).

### EdU assay

Cells were seeded at a density of 1 × 10^4^ cells/well in a 96-well plate, followed by an overnight incubation at 37 °C in a culture incubator. EdU assays were performed using the BeyoClick^TM^ EdU Cell Proliferation Kit with AF555 (Beyotime) according to the manufacturer’s instructions. EdU-positive cells were subsequently subjected to staining with Alexa Fluor 555 and Hoechst 33342. Three random fields of view were selected for analysis, and images were captured using a fluorescence microscope (Leica).

### Clonogenic assay

The clonogenic assay was conducted using cells seeded in a six-well plate. A cell suspension containing 1000 cells was added to each well and cultured in an incubator for 10 days. Thereafter, the cells were fixed with 4% paraformaldehyde. The cells were then subjected to staining with 0.1% crystal violet.

### Wound healing assay

The cells were seeded into 12-well plates. After the cells reached 100% confluence, a sterile 200 μL pipette tip was used to create wounds in the cell monolayer, after which the cells were cultivated in FBS-free medium. Images were captured at 0 h and 48 h of culture, and the width of the wounds was measured with ImageJ (version 1.54 f).

### Transwell Assay

The migration and invasion of cells were assessed using a Transwell assay. Transwell chambers (Falcon) were placed in 24-well plates. In the invasion assay, the upper chamber was coated with Matrigel matrix (Corning). In the migration assay, the upper chamber was not coated with matrix. Subsequently, 200 μL of FBS-free medium containing 2 × 10^4^ cells was added to the upper chamber, and 700 μL of medium containing 20% FBS was added to the lower chamber. The Transwell chambers were then incubated for 48 h or 72 h. The cells located on the surface of the chamber were fixed with 4% formaldehyde, followed by staining with 0.1% crystal violet. Following the removal of cells from the upper surface of the chambers, images of the cells on the lower surface were captured using a microscope. A quantitative analysis was performed using ImageJ (version 1.54 f).

### Cell adhesion assay

The adhesion of cells to ECM-coated 96-well plates was investigated. ECM solutions containing type I collagen (Yeasen), fibronectin (Roche), and laminin (Sigma) were prepared. A volume of 30 µL of ECM solution was added to each well of the 96-well plate and incubated at 37 °C for 1 h. The ECM solution was then removed, and the plate was incubated at 37 °C with 1% bovine serum albumin for an hour. Following the incubation period, the plate was subjected to two gentle washes with FBS-free medium. A cell suspension containing 5 × 10^4^ cells was added to an ECM-coated 96-well plate in a volume of 100 μL. The plate was then incubated at 37 °C with 5% CO₂ for 1–2 h. After the incubation period, the plate was subjected to three washes with phosphate-buffered saline (Gibco) to remove unattached cells. A Cell Counting Kit-8 assay was then performed.

### Anoikis assay, cell viability assay, and caspase-3 activity assay

Anoikis was induced by culturing cells on ultralow attachment plates (Corning). Following the collection of suspended cells, cell viability was assessed using a Trypan Blue Staining Cell Viability Assay Kit (Beyotime), while the detection of apoptosis was facilitated by a Caspase-3 Activity Assay Kit (Beyotime).

### TUNEL assay

The assay was conducted using the TUNEL Cell Apoptosis Detection Kit (Aifang) according to the manufacturer’s protocol. Following three washes with phosphate-buffered saline, the sections were incubated in diaminobenzidine for three minutes. Subsequently, the sections were mounted in mounting medium and observed using a fluorescence microscope. For TUNEL staining, the number of TUNEL-positive nuclei and total nuclei in each field were counted separately, and the apoptotic index was calculated as the percentage of TUNEL-positive cells relative to the total number of cells.

### Immunofluorescence staining

Cells were fixed with 4% formaldehyde and washed three times with phosphate-buffered saline. Afterward, the cells were permeabilized with 0.3% Triton X-100 and blocked with 5% bovine serum albumin. The cells were then incubated with the primary antibody overnight at 4 °C. The cells were subsequently incubated with a fluorescent dye-conjugated secondary antibody and 4’,6-diamidino-2-phenylindole (DAPI). Immunofluorescence staining was analyzed using a confocal laser scanning microscope (Zeiss).

### RNA fluorescence in situ hybridization

FAM-labeled probes targeting circSDK1 were synthesized using a Fluorescence In Situ Hybridization Kit (RiboBio). The cells were subsequently fixed with 4% formaldehyde at 4 °C for 2 h. The cells were permeabilized with 0.3% Triton X-100 for 5 min. After 30 min of pre-hybridization at 37 °C, the cells were hybridized with 5 M probe overnight, and imaging was performed using a confocal laser scanning microscope (Zeiss).

### RNA pull-down assay

The CRTC1 and SDK1 pre-mRNA fragments were cloned from human genomic DNA. After the T7 promoter was inserted upstream of the target sequence, the CRTC1 and SDK1 mRNA fragments were transcribed using an RNAmax-T7 In Vitro Transcription Kit (RiboBio). Biotin labeling of the fragments was performed using the Pierce^TM^ RNA 3’ End Desthiobiotinylation Kit (Thermo Fisher Scientific), while RNA pull-down was performed using the BersinBio^TM^ RNA pull-down Kit (BersinBio) according to the manufacturer’s protocol. The presence of these proteins was detected using western blot analysis.

### ChIP

Chromatin immunoprecipitation experiments were performed using a ChIP kit (BersinBio) in accordance with the manufacturer’s instructions. Briefly, the cells were crosslinked at ambient temperature with 1% formaldehyde and 1.5 mmol/L ethylene glycol bis (succinimidyl succinate). The crosslinked chromatin was sonicated and immunoprecipitated using an antibody. Subsequently, the protein‒DNA complexes were eluted from the antibody, releasing the DNA fragments. The amplified DNA products were subjected to qPCR analysis.

### Dual-luciferase reporter assay

Fragments containing the truncated FAM50A promoter regions, the FAM50A promoter region (WT), or predicted TFAP2C binding site mutants (MUT) were inserted upstream of the firefly luciferase coding sequence in the pmirGLO plasmid. The binding of TFAP2C to the FAM50A promoter was assessed by co-transfecting cells with TFAP2C overexpression vectors or control vectors, along with truncated FAM50A, FAM50A-WT, or FAM50A-MUT vectors. The cells were with Renilla luciferase vectors to standardize the transfection efficiency. These vectors were commercially synthesized by OBiO Technology (Shanghai, China). At two days post-transfection, luciferase activity was detected using a Dual Luciferase Reporter Gene Assay Kit (Beyotime).

### IP‒MS

KYSE30 cells were subjected to lysis on ice with Pierce^TM^ IP lysis buffer (Thermo Fisher Scientific). The whole-cell extracts were then incubated with 5 μg of anti-FAM50A antibody (Zenbio) at 4 °C for one hour, followed by an incubation with magnetic Protein A/G beads (Millipore) at 4 °C for 2 h. The magnetic beads were subjected to three washes with IP lysis buffer. Immune complexes were eluted and analyzed using liquid chromatography‒tandem mass spectrometry (LC‒MS/MS) at PTM BIO (Hangzhou, China).

### RIP assay

Cells were collected for RIP experiments, which were performed using the BersinBio^TM^ RNA Immunoprecipitation Kit (BersinBio) according to the manufacturer’s instructions. Briefly, the cells were collected and lysed with RIP lysis buffer, after which the lysate was incubated overnight at 4 °C with magnetic beads coated with an antibody. The magnetic beads bound to the immunocomplex were washed three times with RIP wash buffer, followed by proteinase K digestion and RNA extraction using a phenol/chloroform/isopropanol (25:24:1) mixture. The input and RIP samples were prepared by Dache Biotechnology (Shanghai, China) for next-generation sequencing.

### EMSA

The GST-tagged recombinant DUSP22 protein was expressed in *E. coli* and purified by affinity chromatography. EMSAs were performed with biotin-labeled wild-type and mutant circSDK1 probes using a chemiluminescence detection kit. For the validation of competition, 10×, 50×, and 100× excess unlabeled cold probes were pre-incubated with DUSP22 prior to the addition of labeled probes. The reaction products were separated on a 6% nondenaturing PAGE gel, transferred to a nylon membrane (Beyotime), and visualized with a chemiluminescence EMSA kit (Beyotime) and a ChemiDoc XRS imaging system (Bio-Rad). Protein expression and purification were performed by CUSABIO (Wuhan, China). Probe synthesis and EMSAs were conducted by GeneCreate Biological Engineering Co., Ltd. (Wuhan, China). Probe sequences are provided in Supplementary Table S1.

### Phosphatase activity assay

The phosphatase activity of DUSP22 was detected in vitro with an EnzChek^®^ Phosphatase Assay Kit (Thermo Fisher). The recombinant GST-tagged DUSP22 protein (CUSABIO) was pre-incubated with serially diluted circSDK1 RNA probes and then mixed with 200 μM DiFMUP substrate in a 100 μL reaction system. After 45 min of incubation at room temperature in the dark, the fluorescence signals were read at 360 nm excitation/455 nm emission using a SpectraMax i3 device (Molecular Devices).

### RNA-seq and quantification

Total RNA was extracted from FAM50A knockdown and control KYSE30 cells using TRIzol (Invitrogen). The RNA quality was subsequently assessed using an Agilent Technologies 2100 Bioanalyzer to ensure that the RNA integrity number was > 7.0. Poly(A) sequencing libraries were prepared according to the Illumina TruSeq-stranded-mRNA protocol. Adenylated mRNA was enriched using oligo-d(T) magnetic beads. The RNA that was purified for circRNA-seq was treated with reagents from the RiboZero rRNA Removal Kit (Illumina) and RNase R (Epicenter) to remove rRNA and linear RNA. RNA-seq libraries were subsequently subjected to paired-end sequencing on the Illumina HiSeq 4000 platform at Dache Biotechnology (Shanghai). Following the acquisition of paired-end reads, the clean reads were aligned to the hg38 genome using HISAT2 (version 2.2.0) and subsequently sorted using SAMtools (version 1.9). The mapped reads were visualized using Integrated Genome Viewer (version 2.16.0). Transcripts were then reconstructed using StringTie (version 1.3.0). Canonical AS events were identified using rMATS (version 4.1.0).

### PDOs

ESCC PDOs were established from the tumor tissue of one patient at Changhai Hospital who provided written informed consent. The PDOs were cultured in medium containing 50% Matrigel (Corning) and complete organoid medium (SHRbio) at 37 °C in an incubator containing 5% CO₂. After drug treatment, the ATP assay reagent was added, and the luminescence intensity was measured using a SpectraMax i3 device (Molecular Devices) to assess cell viability.

### Animal experiments

CDX models were constructed using 4-week-old male nude mice (BALB/c-nu), which were purchased from SiPeiFu Biotechnology (Beijing). Stable lentivirus-transfected KYSE30 cells were injected subcutaneously into each nude mouse. After 3 weeks, the mice were euthanized and dissected. PDX models were established using 4-week-old male NTG mice. Tumor tissue obtained from one donor patient (F0 tumors) was subcutaneously transplanted into NTG mice to induce tumor expansion (F1 tumors). F1 tumors were excised and dissected for the establishment of subsequent generations (F2 tumors). Mice bearing F2 and F3 tumors were randomly assigned to the experimental groups in this study.

### Molecular docking

The molecular docking of nucleic acids and proteins was performed using the AlphaFold server (AlphaFold 3, https://alphafoldserver.com). The molecular docking of small molecules and proteins was performed using AutoDock Vina (version 1.2.5). The molecular docking results were then visualized using the Molecular Operating Environment (version 2022.02) and PyMOL (version 3.1.4.1).

### Molecular dynamics simulation

The Chemistry at Harvard Macromolecular Mechanics (CHARMM-GUI, https://www.charmm-gui.org) was used to construct a simulation system for the small molecule and the protein. A molecular dynamics simulation was performed using GROMACS (version 2022.5), with a simulation time of 100 ns. The RMSD, RMSF, RoG, and number of H-bonds were calculated using GROMACS and visualized using GraphPad Prism (version 10.1.2).

### Statistical analysis

Statistical analyses were performed using GraphPad Prism (version 10.1.2). Quantitative data are presented as the means ± standard deviation. The differences between the groups were analyzed using Student’s *t* test. Spearman’s correlation coefficients were calculated for correlation analyses. Kaplan‒Meier survival curves were generated using R software (version 4.4.1). The Kaplan‒Meier survival curves of patients in the TCGA-ESCC/EAC cohort were analyzed using Kaplan‒Meier Plotter (KM Plotter, https://kmplot.com). Statistical significance was set at *P* < 0.05.

## Supplementary information

Supplementary Information

## Data Availability

The gene expression profiles were obtained from TCGA (https://portal.gdc.cancer.gov/) database. An online Kaplan–Meier plotter database (http://kmplot.com/analysis/) was used in the prognostic analysis. GSE53624 is available for download from the Gene Expression Omnibus (GEO) repository. The protein expression profiles were obtained from the PRIDE database (PXD021701). The data, analytical methods, and study materials supporting the findings of this study will be provided by the corresponding author upon request. The mass spectrometry proteomic data have been deposited into the ProteomeXchange Consortium database via the PRIDE partner repository (http://www.ebi.ac.uk/pride) with the dataset identifier PXD063341. The sequencing data generated by this paper are available through the Genome Sequence Archive (GSA) in the BIG Data Center (https://ngdc.cncb.ac.cn/gsa/), Beijing Institute of Genomics (BIG), Chinese Academy of Sciences, under project number PRJCA044022 (RNA-seq: HRA012618; RIP-seq: HRA012619; circRNA-seq: HRA012617; IP-MS: OMIX011253).
