# Supplementary material for: FAM50A promotes esophageal squamous cell carcinoma progression via splicing-dependent oncogenic circSDK1- and DUSP22-mediated MAPK/FAK activation
Source: Cell Discov. 2026 Oct 20;12:71. doi: 10.1038/s41421-026-00927-z (PMC13680495; doi:10.1038/s41421-026-00927-z)
Supplement: Supplementary file 1 [file 41421_2026_927_MOESM1_ESM.pdf]

Supplementary Figures

Supplementary Fig. S1 The expression of FAM50A in esophageal cancer patient cohorts and esophageal cancer cell lines, related to Fig. 1.

Supplementary Fig. S2 FAM50A knockdown can inhibit proliferation and migration of esophageal squamous cell carcinoma cells, related to Fig. 2.

Supplementary Fig. S3 FAM50A overexpression can promote proliferation and migration of esophageal squamous cell carcinoma cells, related to Fig. 2.

Supplementary Fig. S4 Quantitative analysis of immunohistochemistry and TUNEL assay in ESCC CDX, related to Fig. 2.

Supplementary Fig. S5 FAM50A can regulate ECM adhesion and anoikis resistance of esophageal squamous cell carcinoma cells, related to Fig. 2.

Supplementary Fig. S6 The alternative splicing regulatory function and RNA-binding ability of FAM50A, related to Fig. 3.

Supplementary Fig. S7 Identification results and characteristics of circSDK1, related to Fig. 4.

Supplementary Fig. S8 circSDK1 knockdown can inhibit proliferation and migration of esophageal squamous cell carcinoma cells, related to Fig. 4.

Supplementary Fig. S9 circSDK1 knockdown can inhibit ECM adhesion and anoikis resistance of esophageal squamous cell carcinoma cells, related to Fig. 4.

Supplementary Fig. S10 circSDK1 overexpression can rescue the decreased proliferation and migration induced by FAM50A knockdown, related to Fig. 4.

Supplementary Fig. S11 circSDK1 overexpression can rescue the decreased ECM adhesion and anoikis resistance induced by FAM50A knockdown, related to Fig. 4.

Supplementary Fig. S12 Quantitative analysis of immunohistochemistry and TUNEL assay in ESCC CDX, related to Fig. 4.

Supplementary Fig. S13 circSDK1 knockdown reverses the enhanced proliferation, migration and invasion induced by FAM50A overexpression, related to Fig. 4.

Supplementary Fig. S14 Ectopic expression of circSDK1, but not linear SDK1, restores the

phosphorylation levels of MAPKs and FAK in circSDK1/SDK1 co-knockdown ESCC cells, related to Fig. 4.

Supplementary Fig. S15 CircSDK1 cannot encode a polypeptide or act as a miRNA sponge, related to Fig. 5.

Supplementary Fig. S16 Pharmacological inhibition of MAPKs and FAK can suppress proliferation, migration and invasion of esophageal squamous cell carcinoma cells, related to Fig. 5.

Supplementary Fig. S17 TFAP2C knockdown can reduce proliferation and migration of esophageal squamous cell carcinoma cells, related to Fig. 6.

Supplementary Fig. S18 TFAP2C overexpression can promote proliferation and migration of esophageal squamous cell carcinoma cells, related to Fig. 6.

Supplementary Fig. S19 FAM50A overexpression can rescue the decreased proliferation and migration induced by TFAP2C knockdown, related to Fig. 6.

Supplementary Fig. S20 Upregulated TFAP2C is associated with progression and poor prognosis in esophageal squamous cell carcinoma, related to Fig. 6.

Supplementary Fig. S21 Virtual screen of small-molecule inhibitors of FAM50A, related to Fig. 7.

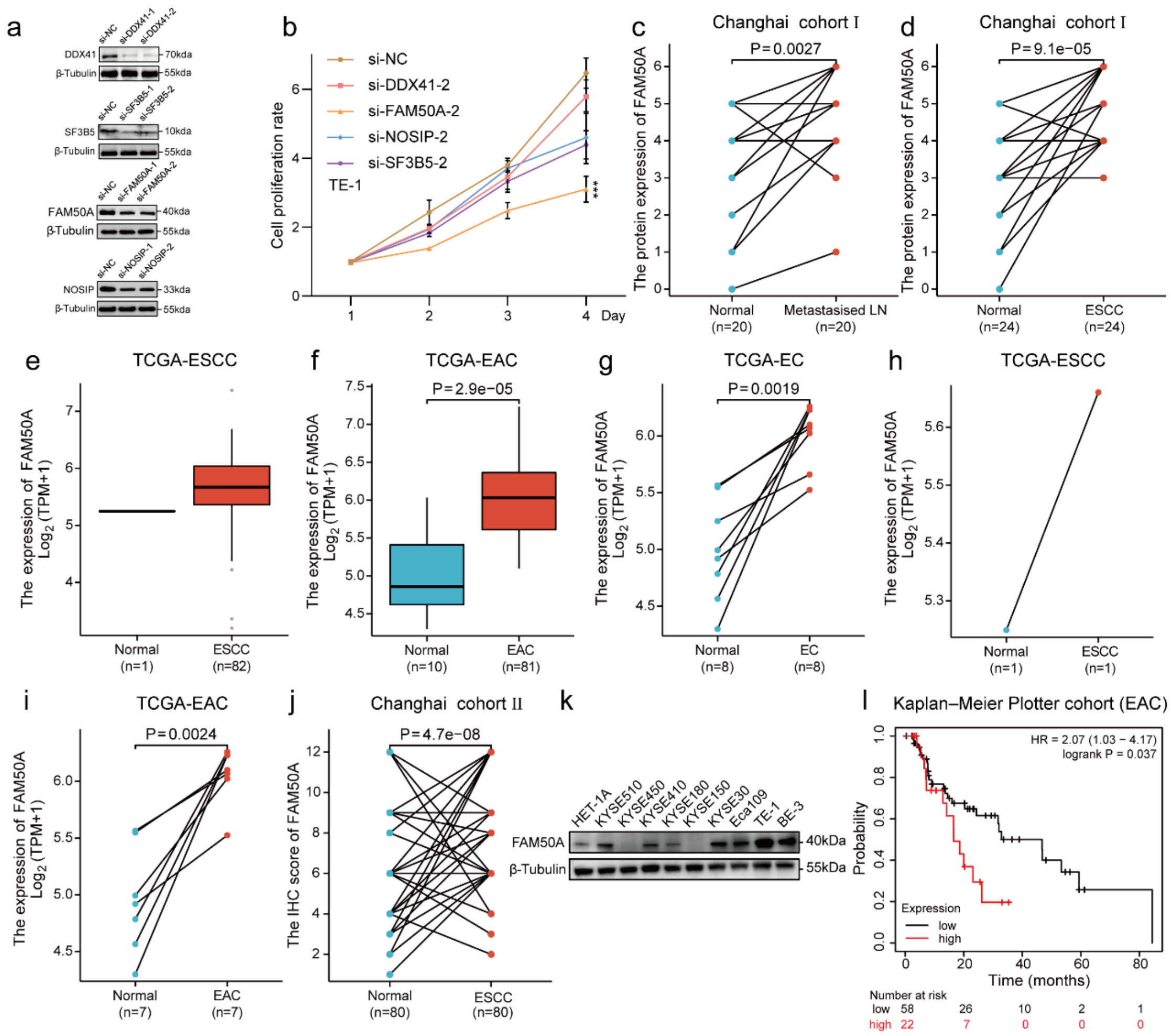

Supplementary Fig. S1 The expression of FAM50A in esophageal cancer patient cohorts and esophageal cancer cell lines, related to Fig. 1. a Protein levels of DDX41, SF3B5, FAM50A, and NOSIP detected by Western blot after corresponding siRNA transfection. **b** RNAi assay of the four candidate splicing factors. **c** FAM50A protein expression in paired metastasized lymph node tissues in Changhai cohort I. **d** FAM50A protein expression in paired ESCC tissues in Changhai cohort I. **e** FAM50A mRNA expression in TCGA-ESCC cohort. **f** FAM50A mRNA expression in TCGA-EAC cohort. **g** FAM50A mRNA expression in paired EC tissues in TCGA-EC cohort. **h** FAM50A mRNA expression in paired ESCC tissues in TCGA-ESCC cohort. **i** FAM50A mRNA expression in paired EAC tissues in TCGA-EAC cohort. **j** FAM50A protein expression in paired ESCC tissues in Changhai cohort II. **k** FAM50A protein expression in EC cell lines compared with HET-1A, a human immortalized normal esophagus epithelial cell line. **l** Kaplan-Meier analysis of the correlation between FAM50A expression and overall survival in TCGA-EAC cohort. The P value was obtained by two-tailed paired Student's t-test (c, d, g, i, j), two-tailed unpaired Student's t-test (b, f), and log-rank test (l). The optimal cut-off value in Kaplan-Meier analysis was determined using the minimum P-

value method. Data are presented as means ± SD unless otherwise stated. TCGA, The Cancer Genome Atlas Program; EC, esophageal cancer; ESCC, esophageal squamous cell carcinoma; EAC, esophageal adenocarcinoma.

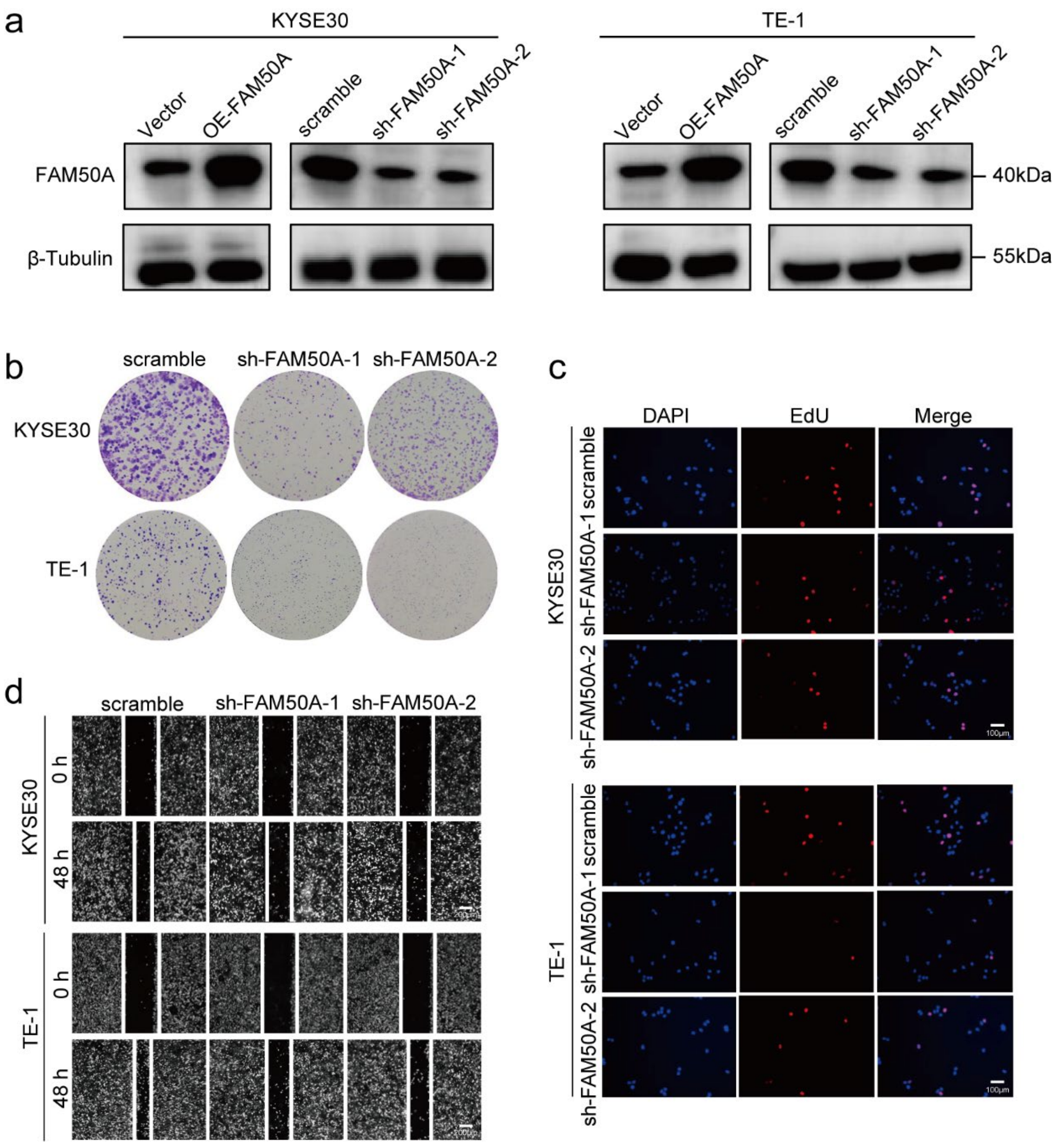

Supplementary Fig. S2 FAM50A knockdown can inhibit proliferation and migration of esophageal squamous cell carcinoma cells, related to Fig. 2. a FAM50A was detected by Western Blot upon FAM50A overexpression and knockdown. The clonogenic assay (**b**), EdU assay (**c**), and wound healing assay (**d**) were performed in ESCC cells with FAM50A knockdown compared to corresponding controls. ESCC, esophageal squamous cell carcinoma.

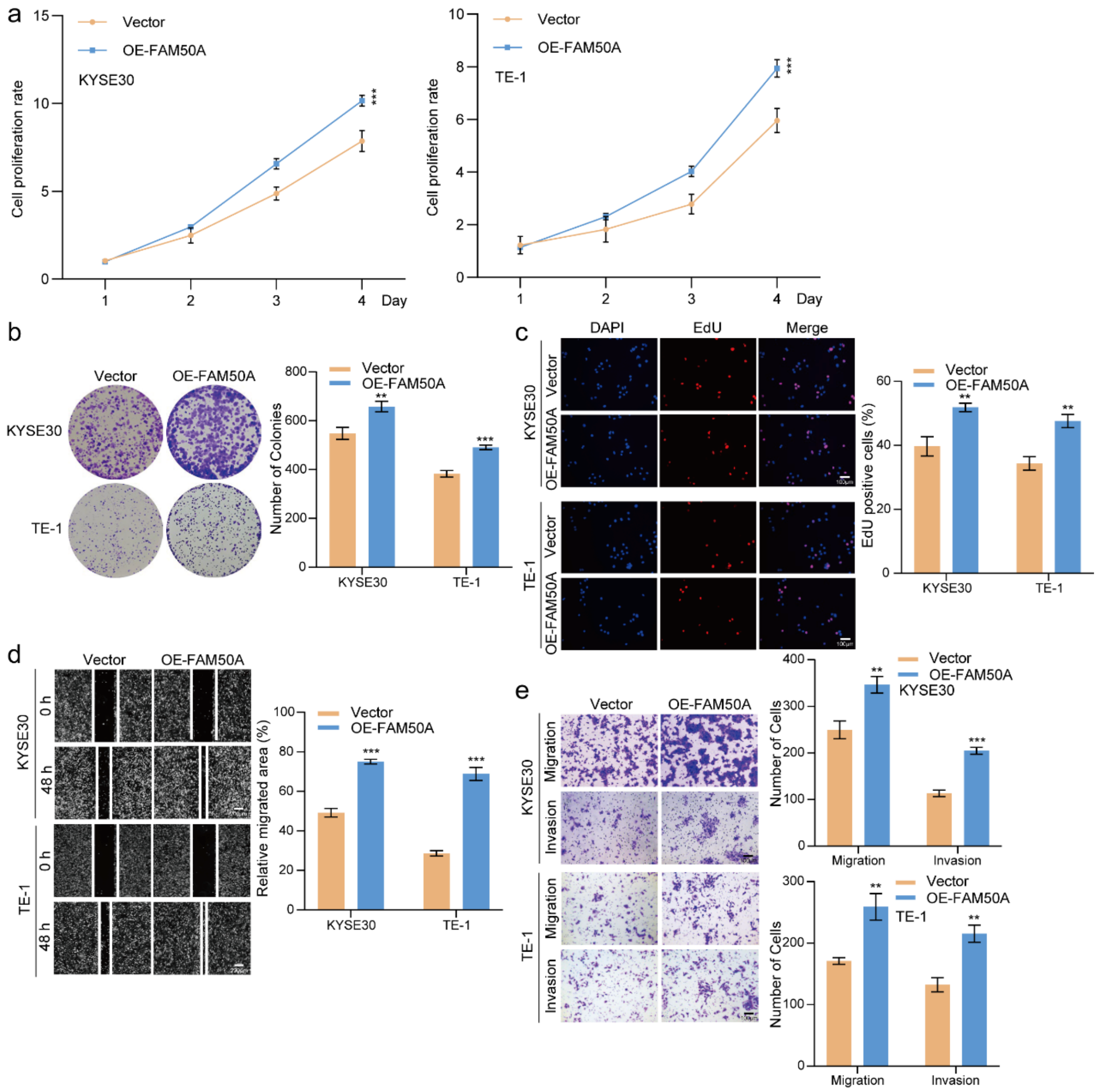

Supplementary Fig. S3 FAM50A overexpression can promote proliferation and migration of esophageal squamous cell carcinoma cells, related to Fig. 2. The cell proliferation assay (**a**), clonogenic assay (**b**), EdU assay (**c**), wound healing assay (**d**), and Transwell assay (**e**) were performed in ESCC cells with FAM50A overexpression compared to corresponding controls (n=3 for clonogenic assay, EdU assay, wound healing assay, and Transwell assay and n=6 for the cell proliferation assay). The P value was obtained by two-tailed unpaired Student's t-test (a-e). Data are presented as means ± SD unless otherwise stated. *p < 0.05, **p < 0.01, ***p < 0.001. ESCC, esophageal squamous cell carcinoma.

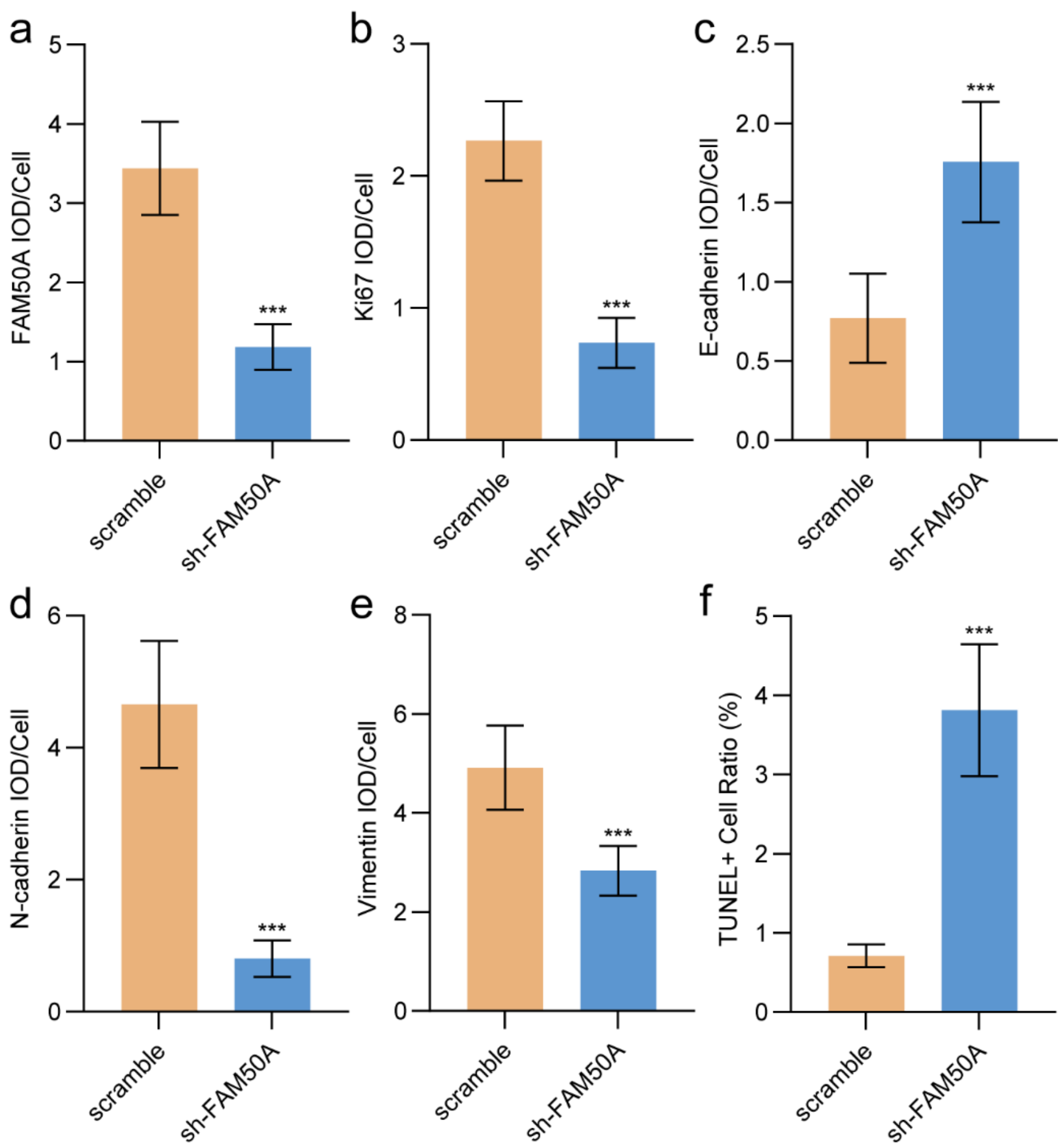

Supplementary Fig. S4 Quantitative analysis of immunohistochemistry and TUNEL assay in ESCC CDX, related to Fig. 2. Immunohistochemical quantification of FAM50A (**a**), Ki67 (**b**), E-cadherin (**c**), N-cadherin (**d**), and Vimentin (**e**), and TUNEL immunofluorescence assay (**f**) were performed in CDX with FAM50A knockdown compared to corresponding scramble controls (n=7 for all assays). The P value was obtained by two-tailed unpaired Student's t-test (a-f). Data are presented as means ± SD unless otherwise stated. *p < 0.05, **p < 0.01, ***p < 0.001. ESCC, esophageal squamous cell carcinoma; CDX, cell line-derived xenograft; IOD, integrated optical density.

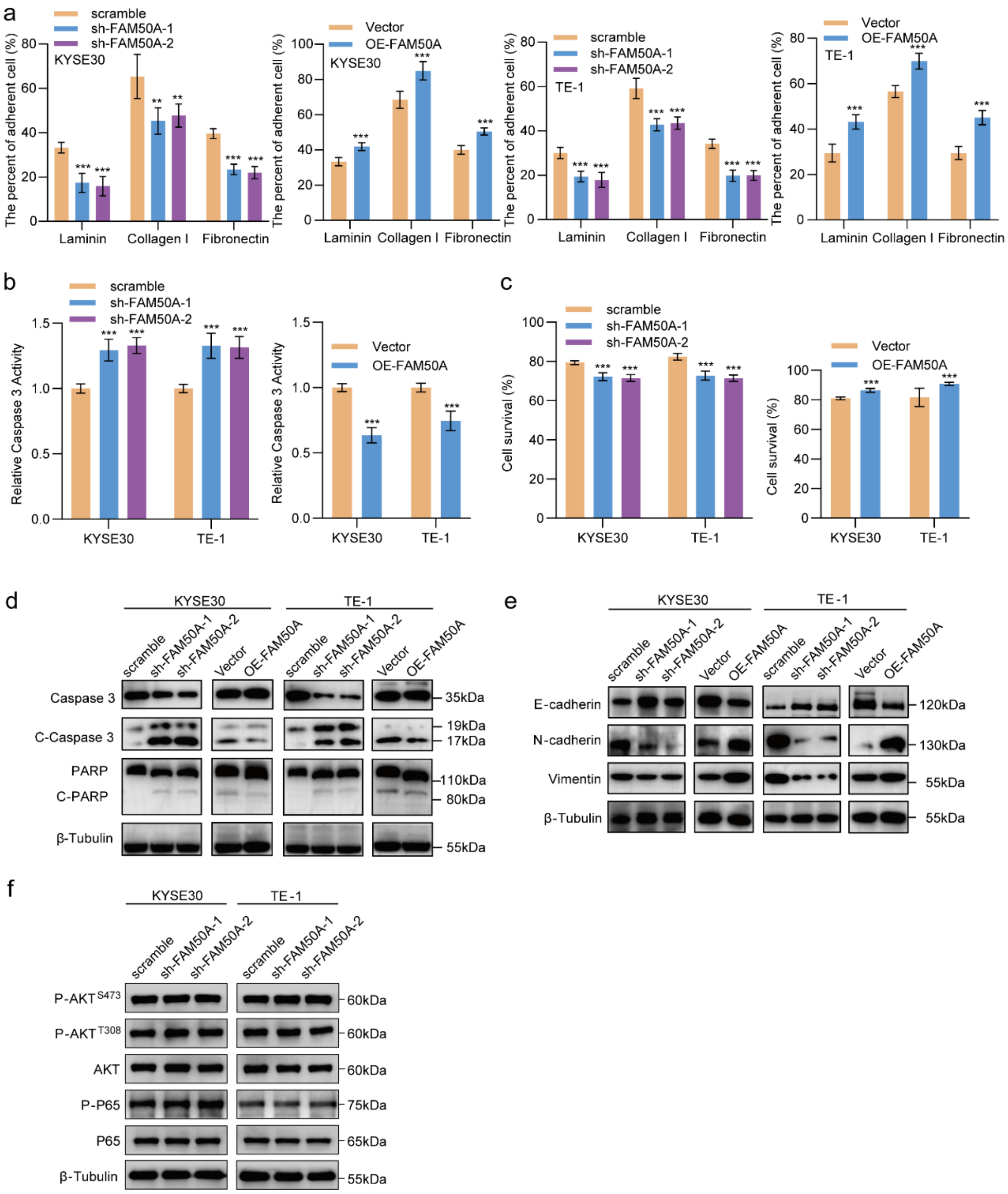

Supplementary Fig. S5 FAM50A can regulate ECM adhesion and anoikis resistance of esophageal squamous cell carcinoma cells, related to Fig. 2. a ECM adhesion assay was conducted to evaluate the adhesion ability of FAM50A knockdown or overexpression cells to laminin, collagen I, and fibronectin (n=6). **b** The anoikis activity was evaluated by caspase 3 activity assay when FAM50A knockdown or overexpression cells were cultured in suspension condition for 48 hours (n=6). **c** Cell viability was analyzed by Trypan blue exclusion assay (n=5). FAM50A knockdown or overexpression cells were cultured in suspension condition for 48 hours. **d** Apoptotic markers were measured by Western Blot. FAM50A knockdown or overexpression cells were cultured in suspension condition. **e** EMT markers were measured by

Western Blot. **f** AKT and NF-κB p65 signaling markers were measured by Western Blot. The P value was obtained by two-tailed unpaired Student's t-test (a-c), and the results are presented as the mean ± SD. *p < 0.05, **p < 0.01, ***p < 0.001. ECM, extracellular matrix; EMT, epithelial-mesenchymal transition.

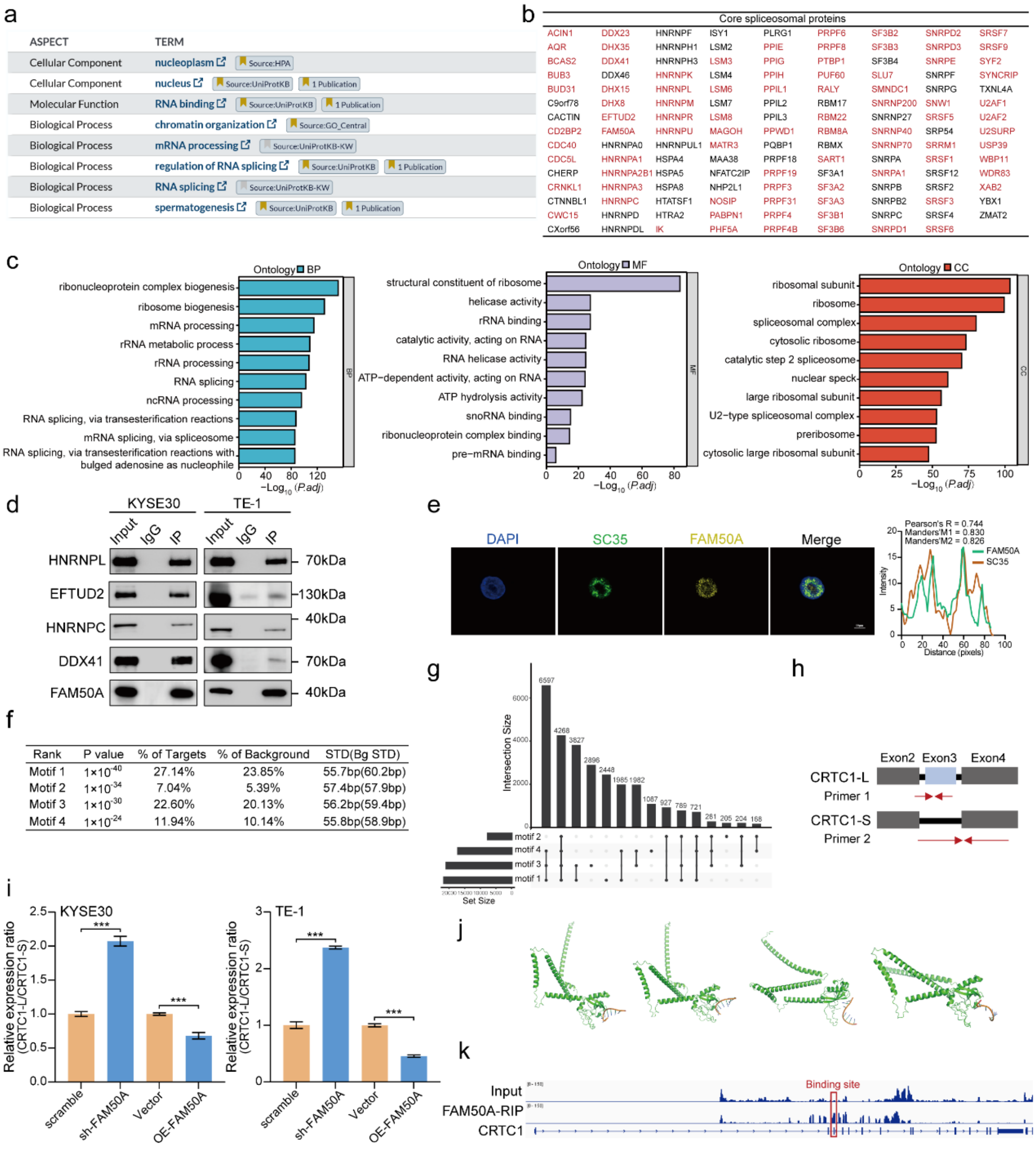

Rank	P value	% of Targets	% of Background	STD(Bg STD)
Motif 1	1×10^{-40}	27.14%	23.85%	55.7bp(60.2bp)
Motif 2	1×10^{-34}	7.04%	5.39%	57.4bp(57.9bp)
Motif 3	1×10^{-30}	22.60%	20.13%	56.2bp(59.4bp)
Motif 4	1×10^{-24}	11.94%	10.14%	55.8bp(58.9bp)

Supplementary Fig. S6 The alternative splicing regulatory function and RNA-binding ability of FAM50A, related to Fig. 3. a The function of FAM50A in the UniProt database. **b** Proteins interacting with FAM50A among the 134 core splicing factors. FAM50A-associated proteins from IP-MS were colored in red. **c** GO enrichment analysis was conducted on proteins interacting with FAM50A. **d** FAM50A-interacting proteins (HNRNPL, EFTUD2, HNRNPC,

and DDX41) were detected by Co-IP assay. **e** Immunofluorescence assay showed the co-localization of FAM50A (yellow) with splicing factor SC35 (green) in punctate nuclear speckles (KYSE30). The quantification and analysis of co-localization was performed with Coloc 2 and Plot Profile (Pearson's R = 0.744, Manders' M1 = 0.830, Manders' M2 = 0.826). **f** Statistical results of the top four FAM50A-binding motifs ranked by HOMER-calculated P values. **g** Upset plot of the distribution of motifs 1-4 in the FAM50A-binding regions. **h** Schematic showing the isoform-specific qPCR primers of CRTC1-L/S. **i** qPCR confirmation of FAM50A-dependent alternative splicing of CRTC1 in KYSE30 and TE-1 cells (n=3). Both alternatively spliced isoforms of CRTC1 were examined using corresponding qPCR primers by qPCR analysis upon FAM50A knockdown or overexpression. **j** Molecular docking of the four motifs and FAM50A was performed using AlphaFold 3. **k** Genome browser representation of FAM50A binding over CRTC1 loci as determined by RIP-seq.

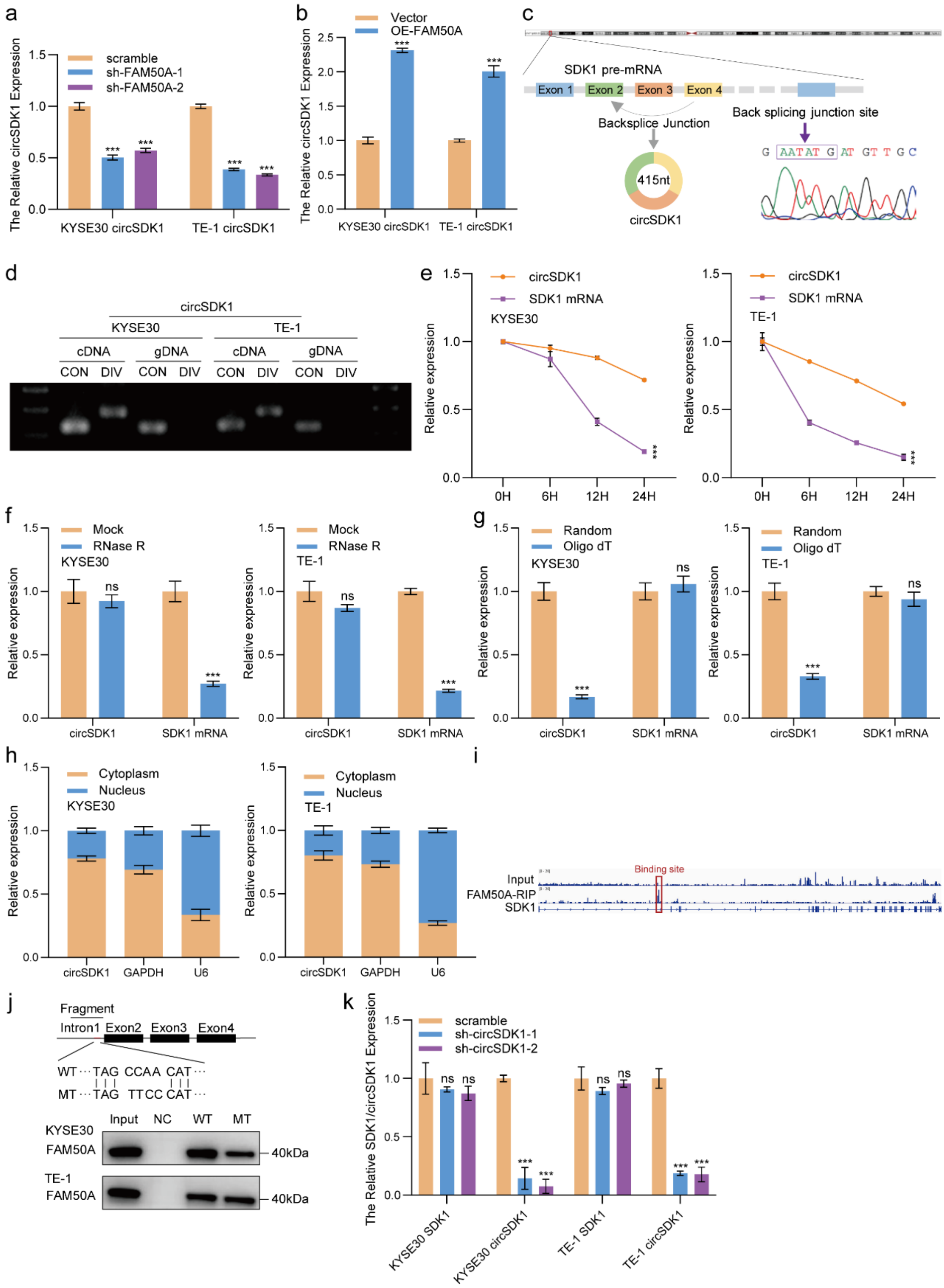

Supplementary Fig. S7 Identification results and characteristics of circSDK1, related to Fig. 4. a qPCR confirmation of FAM50A-dependent backsplicing of circSDK1 upon FAM50A knockdown. **b** qPCR confirmation of FAM50A-dependent backsplicing of circSDK1 upon FAM50A overexpression. **c** The analysis confirmed that circSDK1 is produced through the circularization of exons 2-4 of SDK1, with the “head-to-tail” splicing junction verified by qPCR

and Sanger sequencing, as indicated by a purple arrow. **d** qPCR assay and agarose gel electrophoresis indicating the detection of circSDK1 using divergent and convergent primers from cDNA or gDNA. The stability of circSDK1 and SDK1 was evaluated after Actinomycin D (**e**) or RNase R treatment (**f**). **g** Random or Oligo dT primers were used in reverse transcription experiments, and the analysis of the RNA levels by qPCR. **h** Nuclear-cytoplasmic fractionation assay identified the localization of circSDK1. **i** Genome browser representation of FAM50A binding over SDK1 loci as determined by RIP-seq. **j** RNA pull-down assay showed the interaction between SDK1 pre-mRNA and FAM50A protein. **k** qPCR analysis was conducted to measure the expression levels of circSDK1 and SDK1 upon circSDK1 knockdown. The P value was obtained by two-tailed unpaired Student's t-test (a, b, e-g, k). Data are presented as means ± SD unless otherwise stated. $*p < 0.05$, $**p < 0.01$, $***p < 0.001$; ns, no significance. cDNA, complementary DNA. gDNA, genomic DNA.

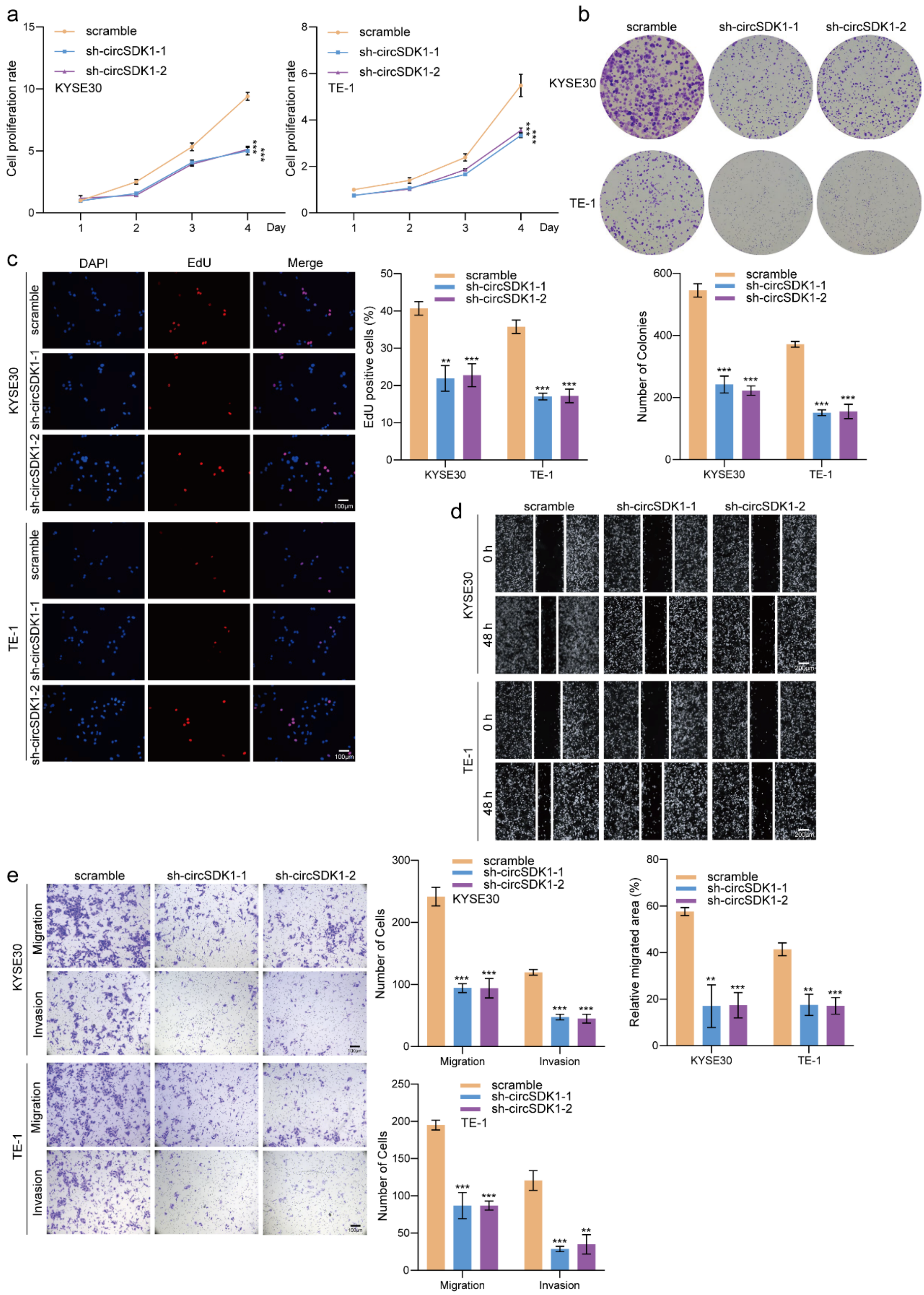

Supplementary Fig. S8 circSDK1 knockdown can inhibit proliferation and migration of esophageal squamous cell carcinoma cells, related to Fig. 4. The cell proliferation assay (**a**), clonogenic assay (**b**), EdU assay (**c**), wound healing assay (**d**), and Transwell assay (**e**) were performed in ESCC cells with circSDK1 knockdown compared to corresponding controls (n=3

for clonogenic assay, EdU assay, wound healing assay, and Transwell assay and n=8 for the cell proliferation assay). The P value was obtained by two-tailed unpaired Student's t-test (a-e). Data are presented as means ± SD unless otherwise stated. *p < 0.05, **p < 0.01, ***p < 0.001. ESCC, esophageal squamous cell carcinoma.

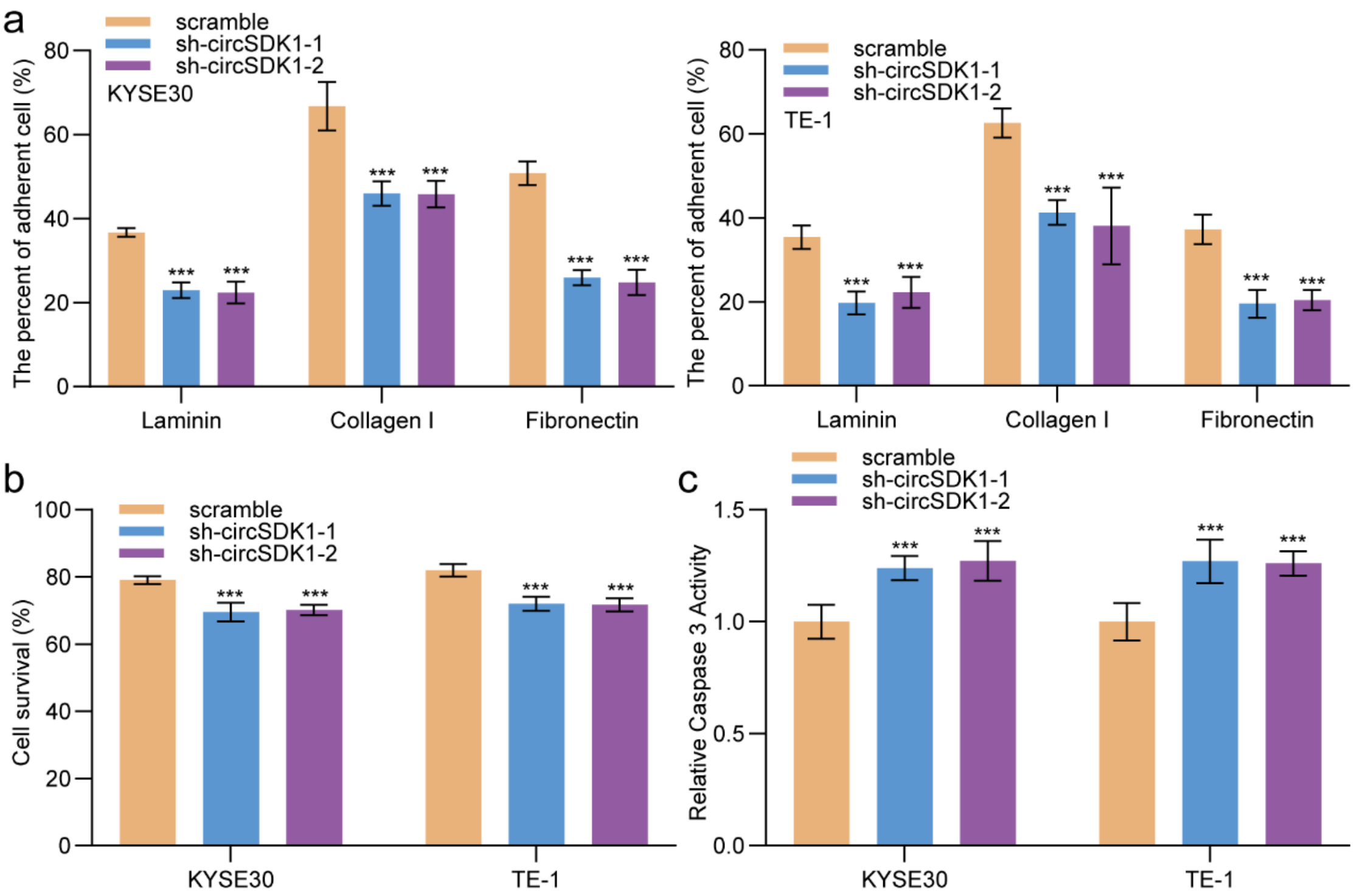

Supplementary Fig. S9 circSDK1 knockdown can inhibit ECM adhesion and anoikis resistance of esophageal squamous cell carcinoma cells, related to Fig. 4. a Cell adhesion assay was conducted to evaluate the adhesion ability of circSDK1 knockdown cells to laminin, collagen I, and fibronectin (n=6). Trypan blue exclusion assay (**b**) and caspase 3 activity assay (**c**) were performed in circSDK1 knockdown cells cultured in suspension condition (n=6). The P value was obtained by two-tailed unpaired Student's t-test (a-c), and the results are presented as the mean ± SD. *p < 0.05, **p < 0.01, ***p < 0.001. ECM, extracellular matrix.

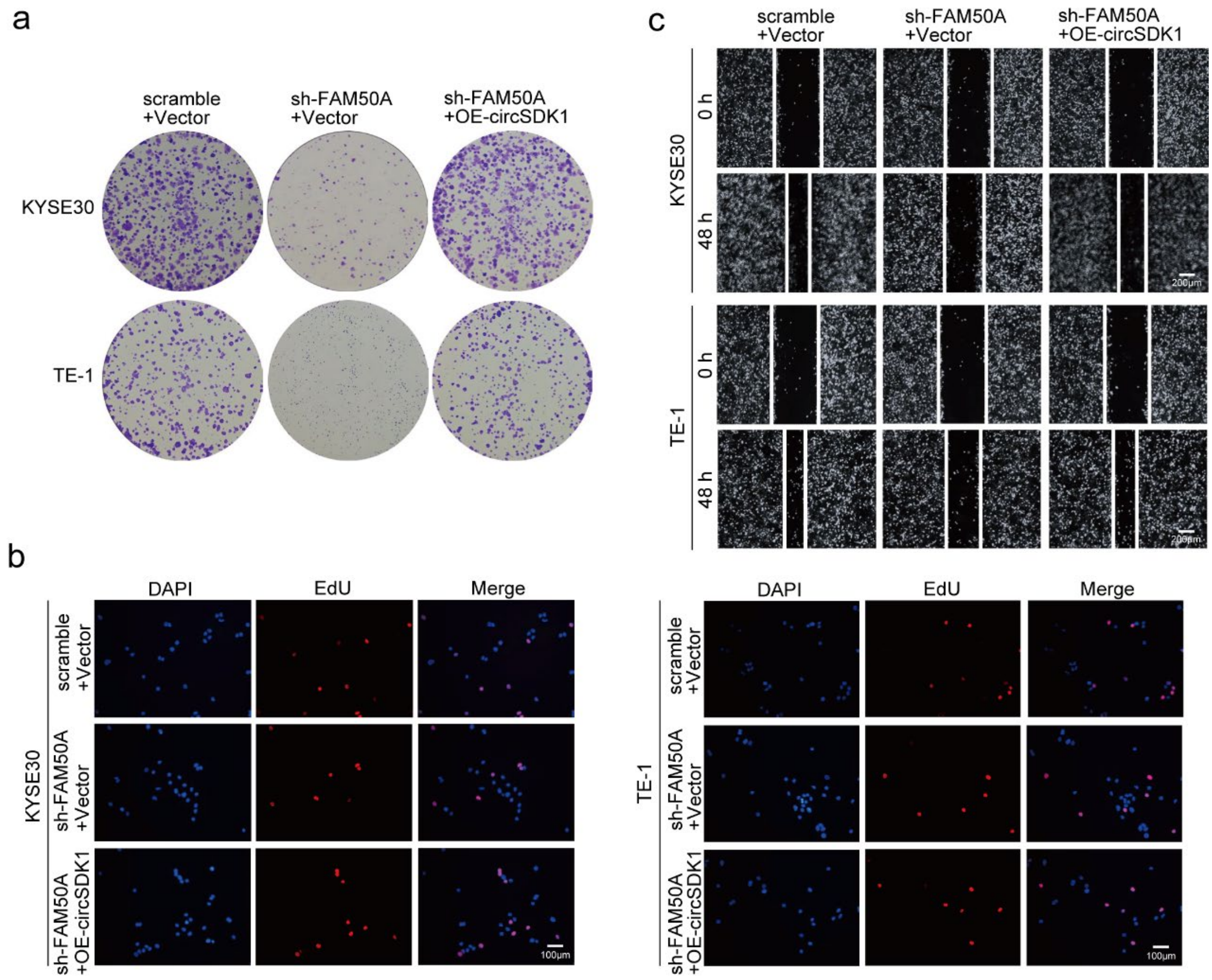

Supplementary Fig. S10 circSDK1 overexpression can rescue the decreased proliferation and migration induced by FAM50A knockdown, related to Fig. 4. The clonogenic assay (**a**), EdU assay (**b**), and wound healing assay (**c**) were performed in ESCC cells to investigate the role of circSDK1 in rescuing the loss of FAM50A. ESCC, esophageal squamous cell carcinoma.

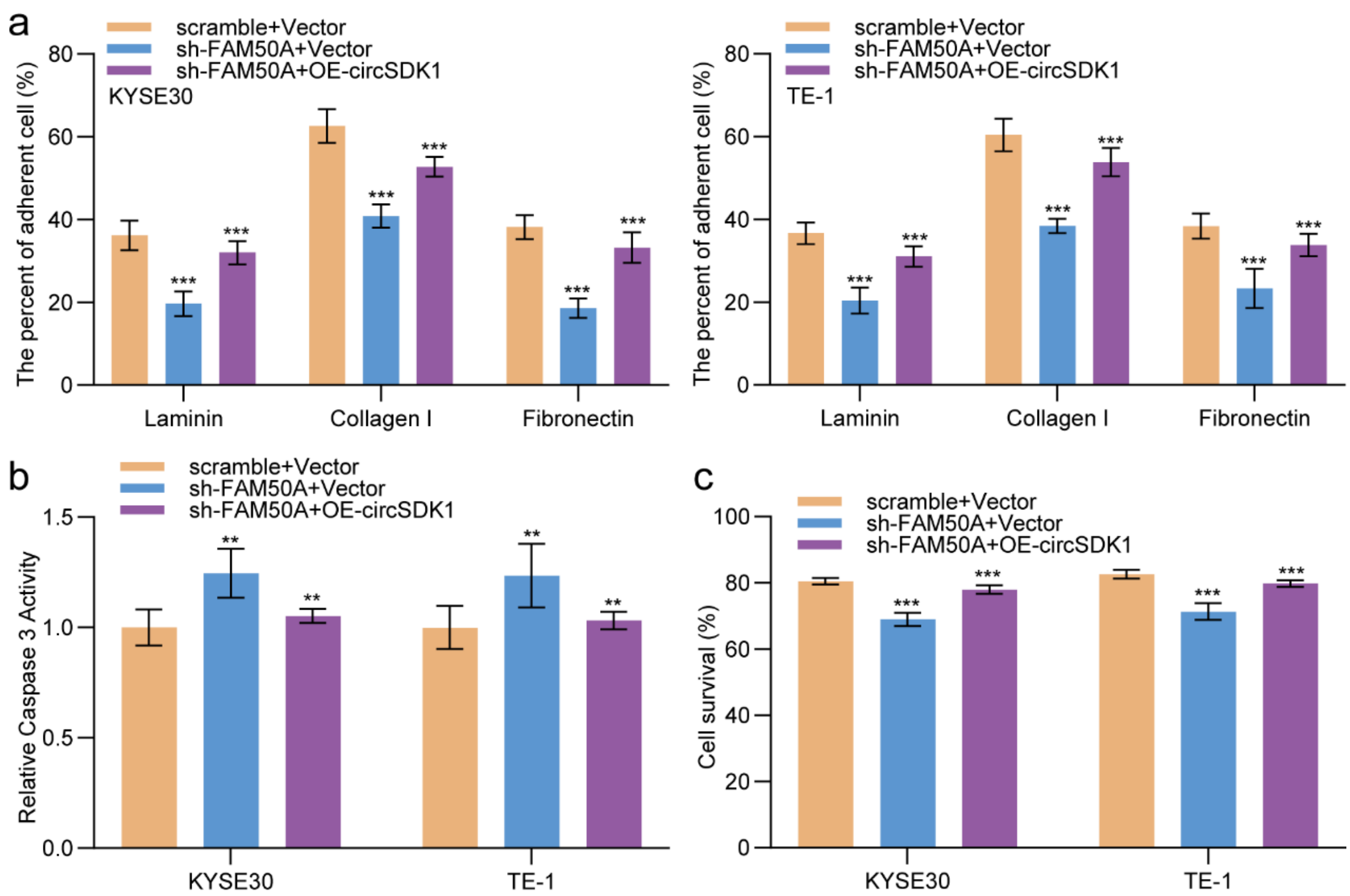

Supplementary Fig. S11 circSDK1 overexpression can rescue the decreased ECM adhesion and anoikis resistance induced by FAM50A knockdown, related to Fig. 4. a Cell adhesion assay was conducted to evaluate the adhesion ability of circSDK1 in rescuing the loss of FAM50A (n=6). Caspase 3 activity assay (**b**) and Trypan blue exclusion assay (**c**) were performed to evaluate the anoikis activity of circSDK1 in rescuing the loss of FAM50A in suspension condition (n=6). The P value was obtained by two-tailed unpaired Student's t-test (a-c), and the results are presented as the mean ± SD. *p < 0.05, **p < 0.01, ***p < 0.001. ECM, extracellular matrix.

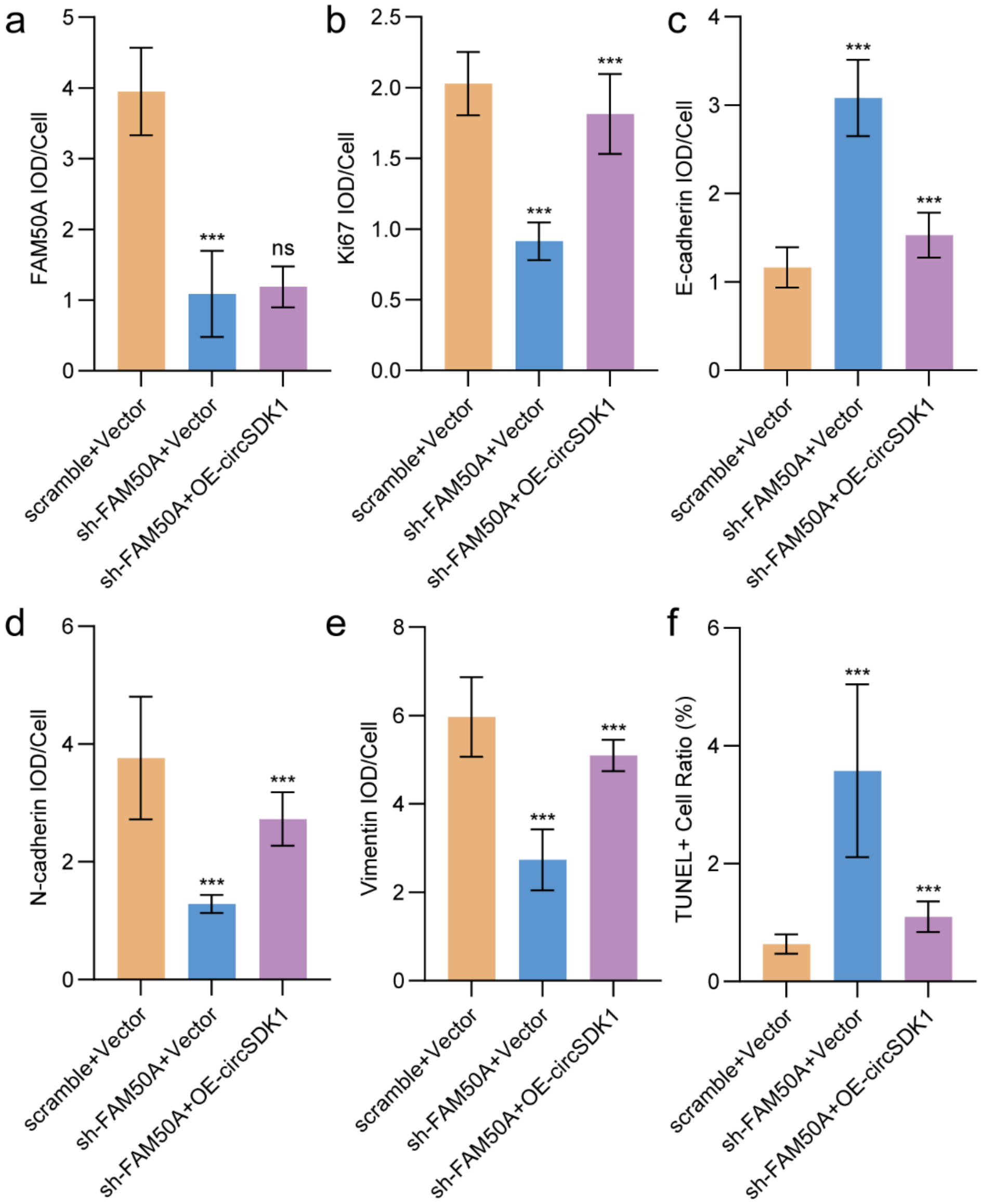

Supplementary Fig. S12 Quantitative analysis of immunohistochemistry and TUNEL assay in ESCC CDX, related to Fig. 4. Immunohistochemical quantification of FAM50A (**a**), Ki67 (**b**), E-cadherin (**c**), N-cadherin (**d**), and Vimentin (**e**), and TUNEL immunofluorescence assay (**f**) were performed in ESCC CDX models with FAM50A knockdown alone or combined with circSDK1 overexpression, compared to scramble control (n=7 for all assays). The P values were obtained by two-tailed unpaired Student's t-test (a-f). Data are presented as means ± SD unless otherwise stated. *p < 0.05, **p < 0.01, ***p < 0.001; ns, no significance. ESCC, esophageal squamous cell carcinoma; CDX, cell line-derived xenograft; IOD, integrated optical density.

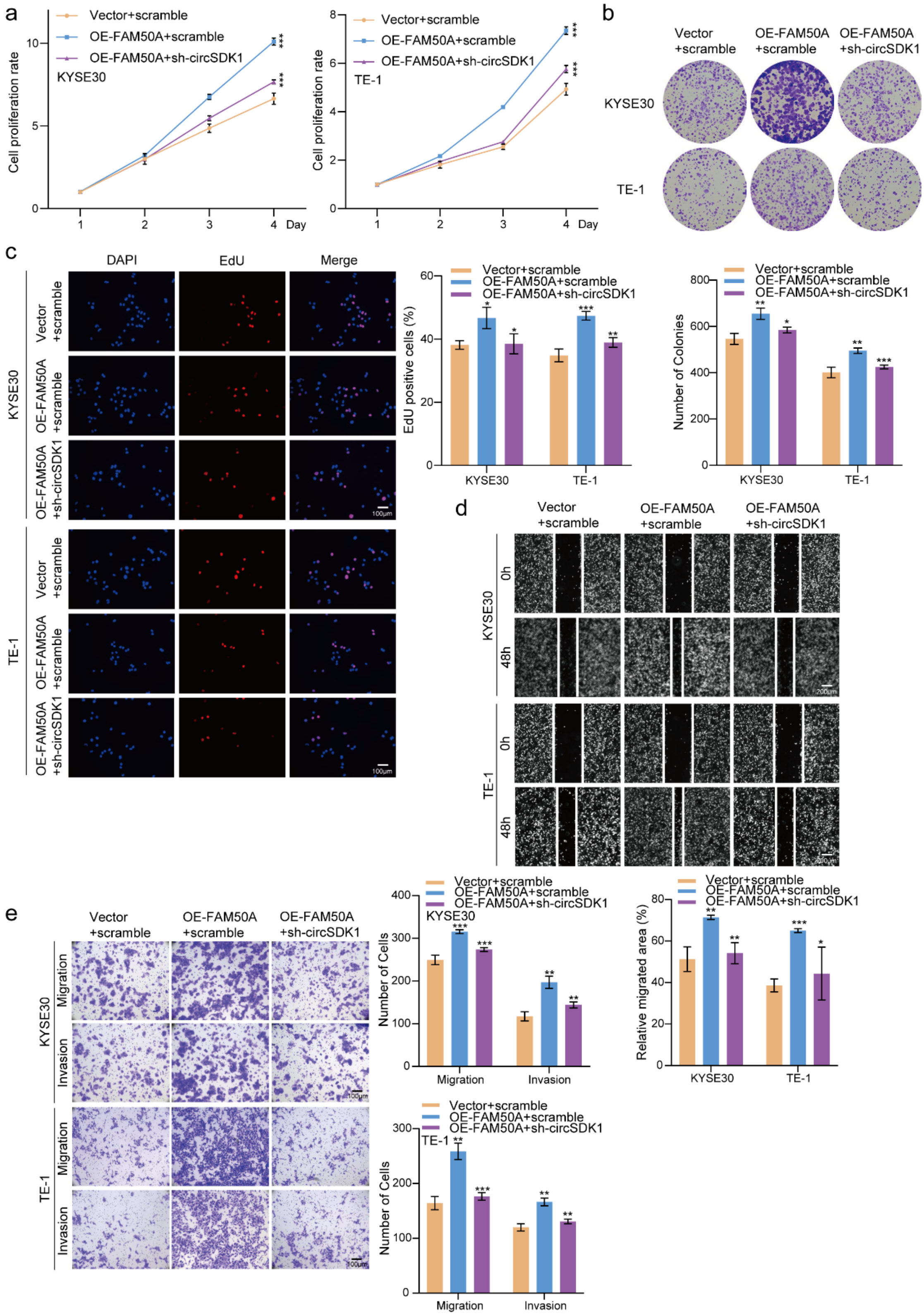

Supplementary Fig. S13 circSDK1 knockdown reverses the enhanced proliferation, migration and invasion induced by FAM50A overexpression, related to Fig. 4. The cell proliferation assay (**a**), clonogenic assay (**b**), EdU assay (**c**), wound healing assay (**d**), and Transwell migration and invasion assays (**e**) were performed in ESCC cells to investigate the

role of circSDK1 in reversing the malignant phenotypes induced by FAM50A overexpression (n=3 for clonogenic assay, EdU assay, wound healing assay, and Transwell assays and n=6 for the cell proliferation assay). The P values were obtained by two-tailed unpaired Student's t-test (a-e). Data are presented as means ± SD unless otherwise stated. *p < 0.05, **p < 0.01, ***p < 0.001. ESCC, esophageal squamous cell carcinoma.

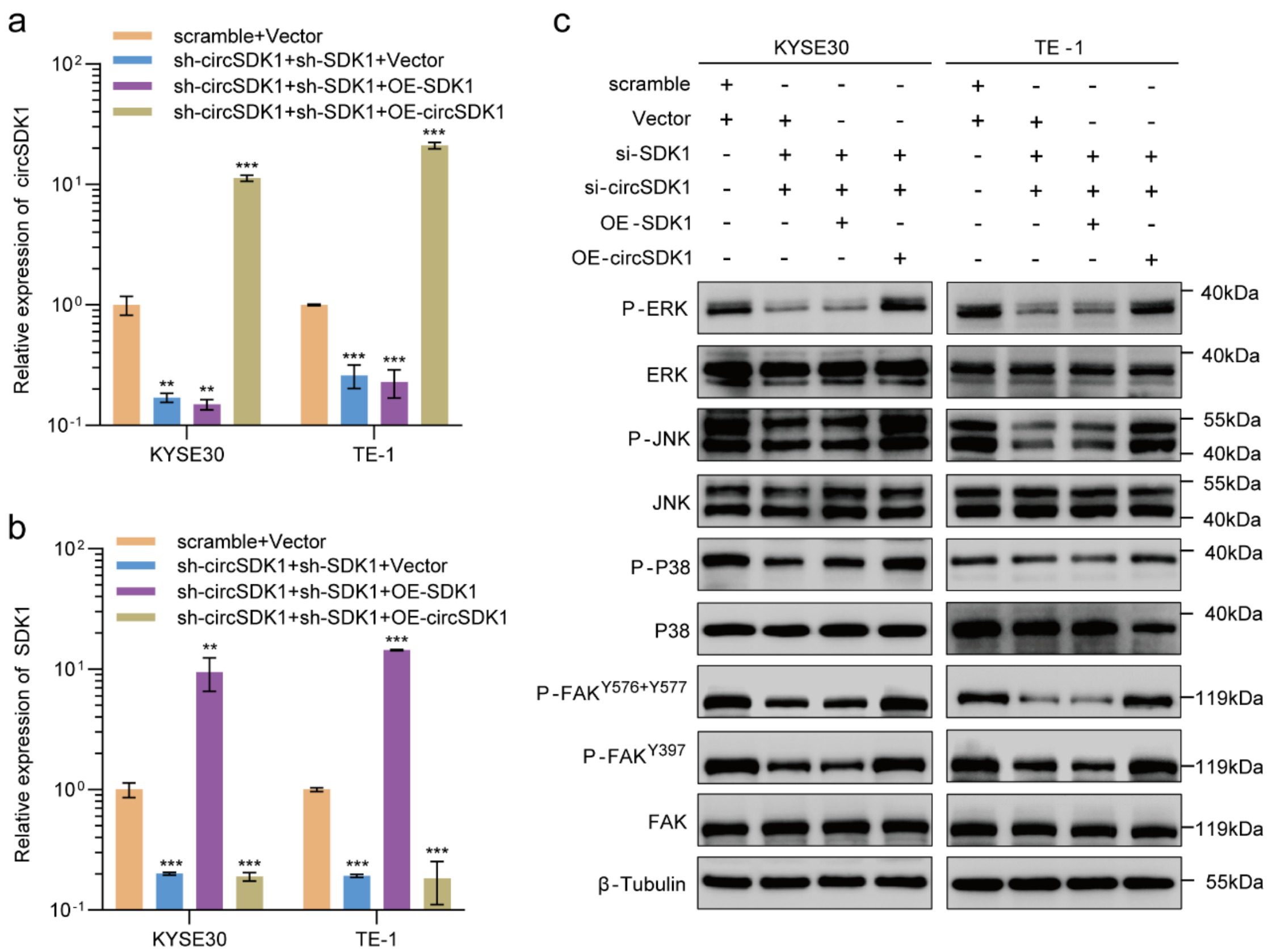

Supplementary Fig. S14 Ectopic expression of circSDK1, but not linear SDK1, restores the phosphorylation levels of MAPKs and FAK in circSDK1/SDK1 co-knockdown ESCC cells, related to Fig. 4. The qPCR assays (**a**, **b**) were performed in KYSE30 and TE-1 cells to detect the relative RNA levels of circSDK1 and linear SDK1 (n=3). Western blot analysis (**c**) was performed to detect the phosphorylated and total protein levels of MAPKs and FAK in the indicated groups. The P values were obtained by two-tailed unpaired Student's t-test (a, b). Data are presented as means ± SD unless otherwise stated. *p < 0.05, **p < 0.01, ***p < 0.001. ESCC, esophageal squamous cell carcinoma.

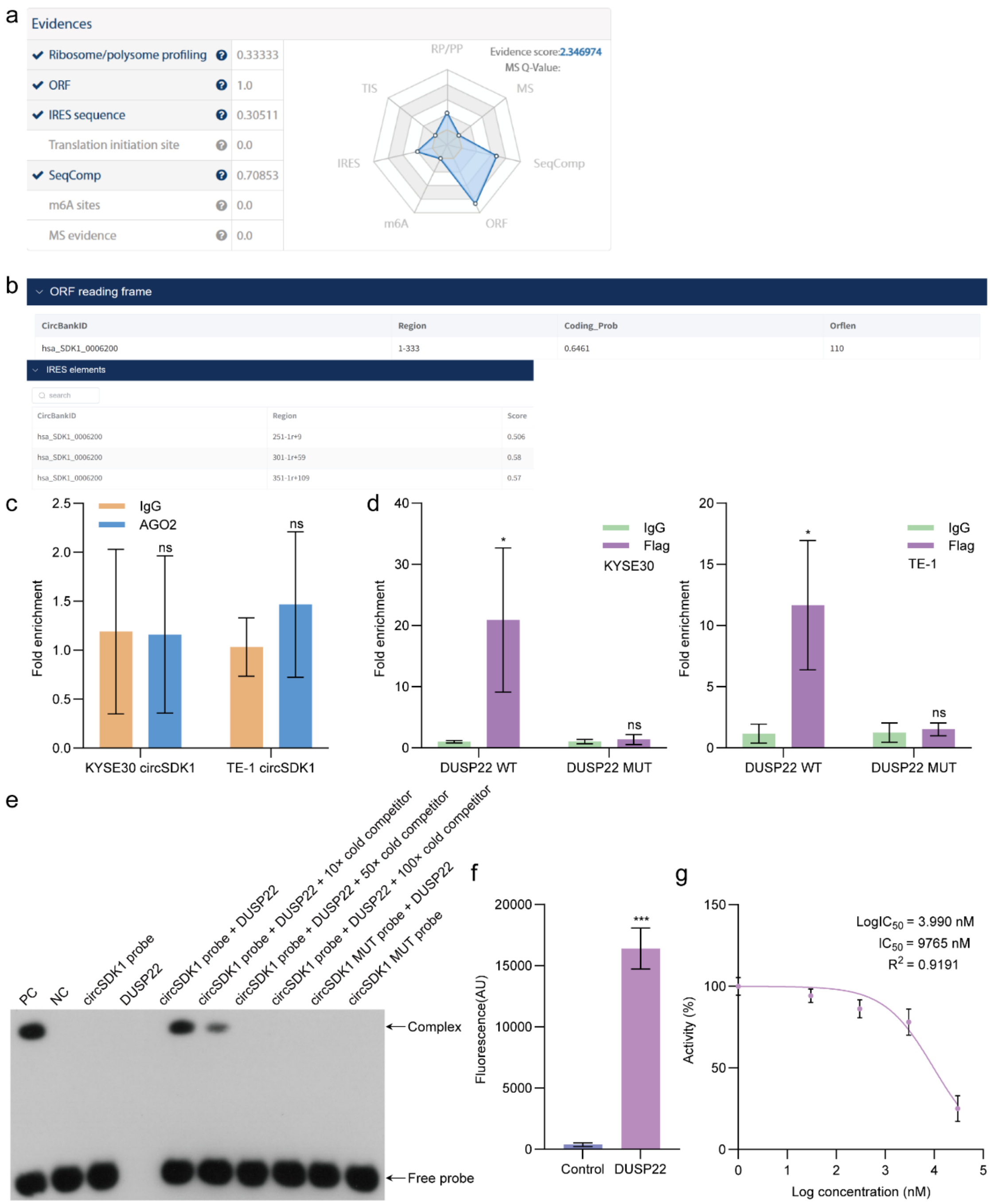

Supplementary Fig. S15 circSDK1 cannot encode a polypeptide or act as a miRNA sponge, related to Fig. 5. a The message about the ORF or IRES of circSDK1 in the TRANSCIRC database. **b** The message about the ORF or IRES of circSDK1 in the circBank database. **c** RIP-qPCR was performed to investigate the interaction between AGO2 and circSDK1 with the anti-AGO2 antibody. **d** RIP-qPCR assay was performed with an anti-Flag antibody to examine the enrichment of circSDK1 pulled down by wild-type (WT) and mutant (MUT) DUSP22 protein. **e** EMSA was performed to verify the direct binding between DUSP22 protein and circSDK1 using biotin-labeled probes. Phosphatase activity assay for DUSP22 (**f**) and inhibition by

circSDK1 (**g**). The P values were obtained by two-tailed unpaired Student's t-test (c, d, f). Data are presented as means ± SD unless otherwise stated. *$p < 0.05$, **$p < 0.01$, ***$p < 0.001$; ns, no significance. ORF, open reading frame; IRES, internal ribosome entry site.

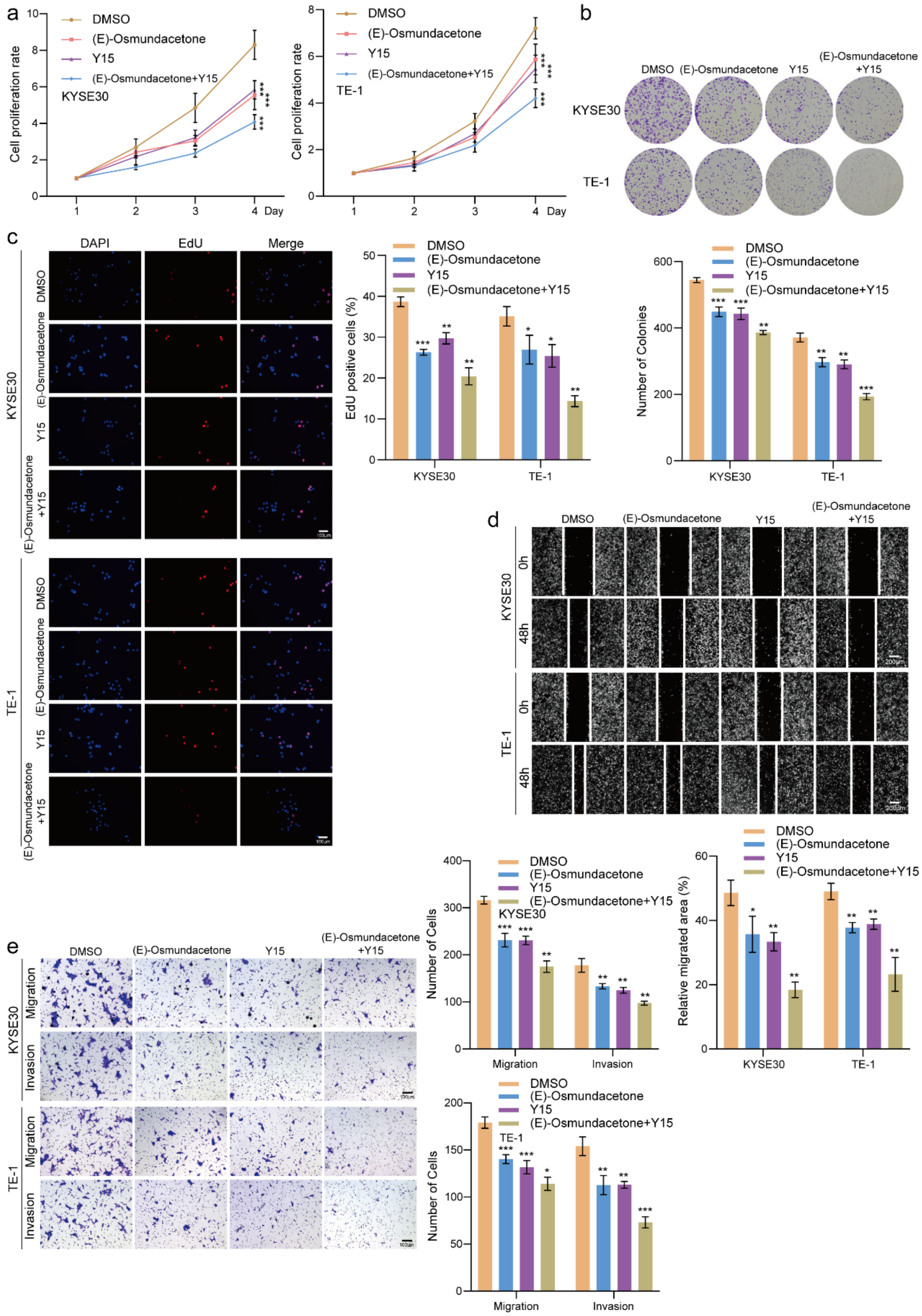

Supplementary Fig. S16 Pharmacological inhibition of MAPKs and FAK can suppress proliferation, migration and invasion of esophageal squamous cell carcinoma cells, related to Fig. 5. The cell proliferation assay (**a**), clonogenic assay (**b**), EdU assay (**c**), wound healing assay (**d**), and Transwell migration and invasion assays (**e**) were performed in KYSE30 and

TE-1 cells treated with (E)-Osmundacetone, Y15, or their combination, compared to corresponding DMSO controls (n=3 for clonogenic assay, EdU assay, wound healing assay, and Transwell assays and n=6 for cell proliferation assay). The P value was obtained by two-tailed unpaired Student's t-test (a-e). Data are presented as means ± SD unless otherwise stated. *p < 0.05, **p < 0.01, ***p < 0.001.

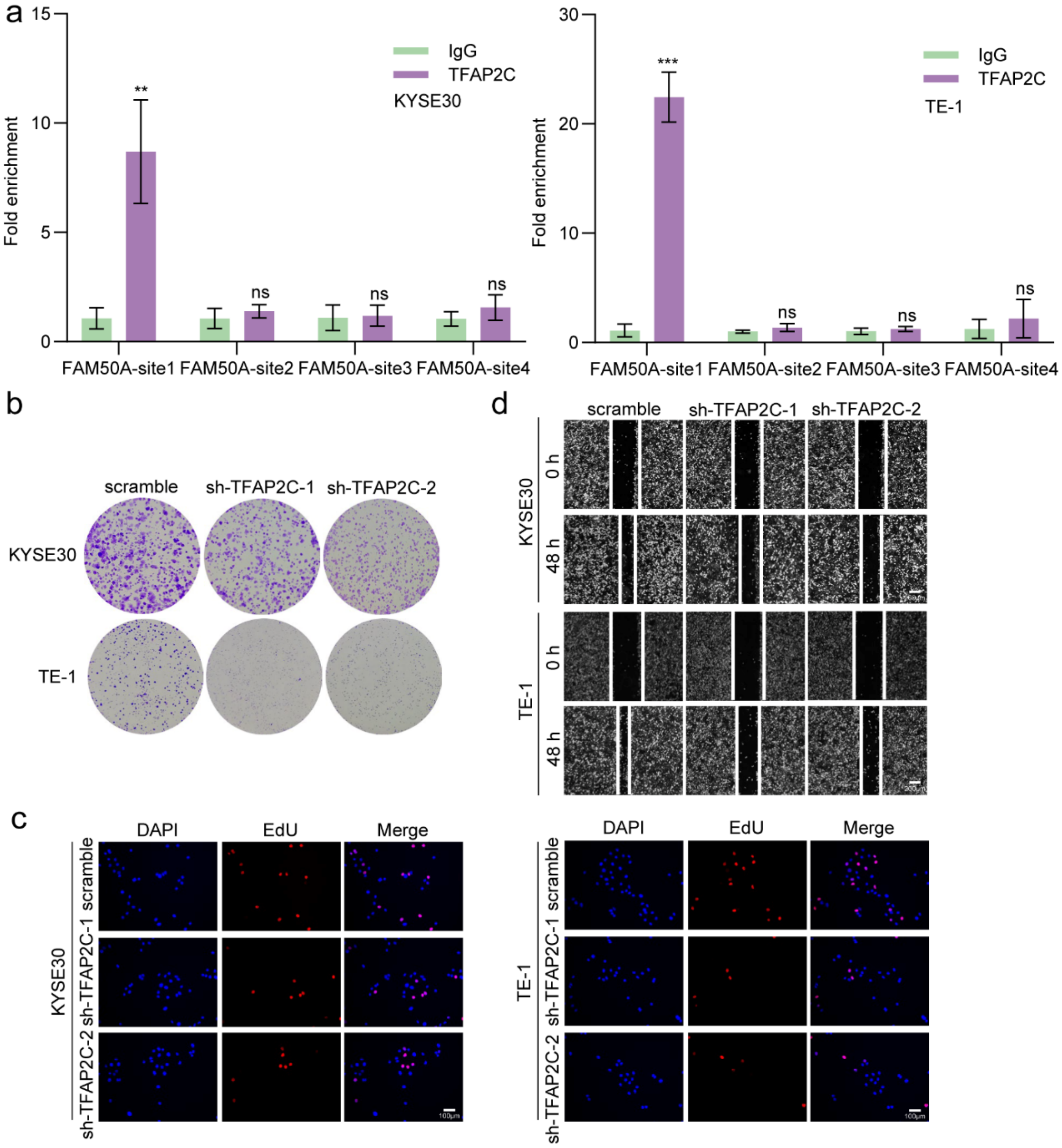

Supplementary Fig. S17 TFAP2C knockdown can reduce proliferation and migration of esophageal squamous cell carcinoma cells, related to Fig. 6. a ChIP analysis of TFAP2C binding to the FAM50A promoter at four predicted binding sites. The clonogenic assay (**b**), EdU assay (**c**), and wound healing assay (**d**) were performed in ESCC cells with TFAP2C knockdown compared to corresponding controls. The P values were obtained by two-tailed

unpaired Student's t-test (a). Data are presented as means ± SD unless otherwise stated. *p < 0.05, **p < 0.01, ***p < 0.001; ns, no significance. ESCC, esophageal squamous cell carcinoma.

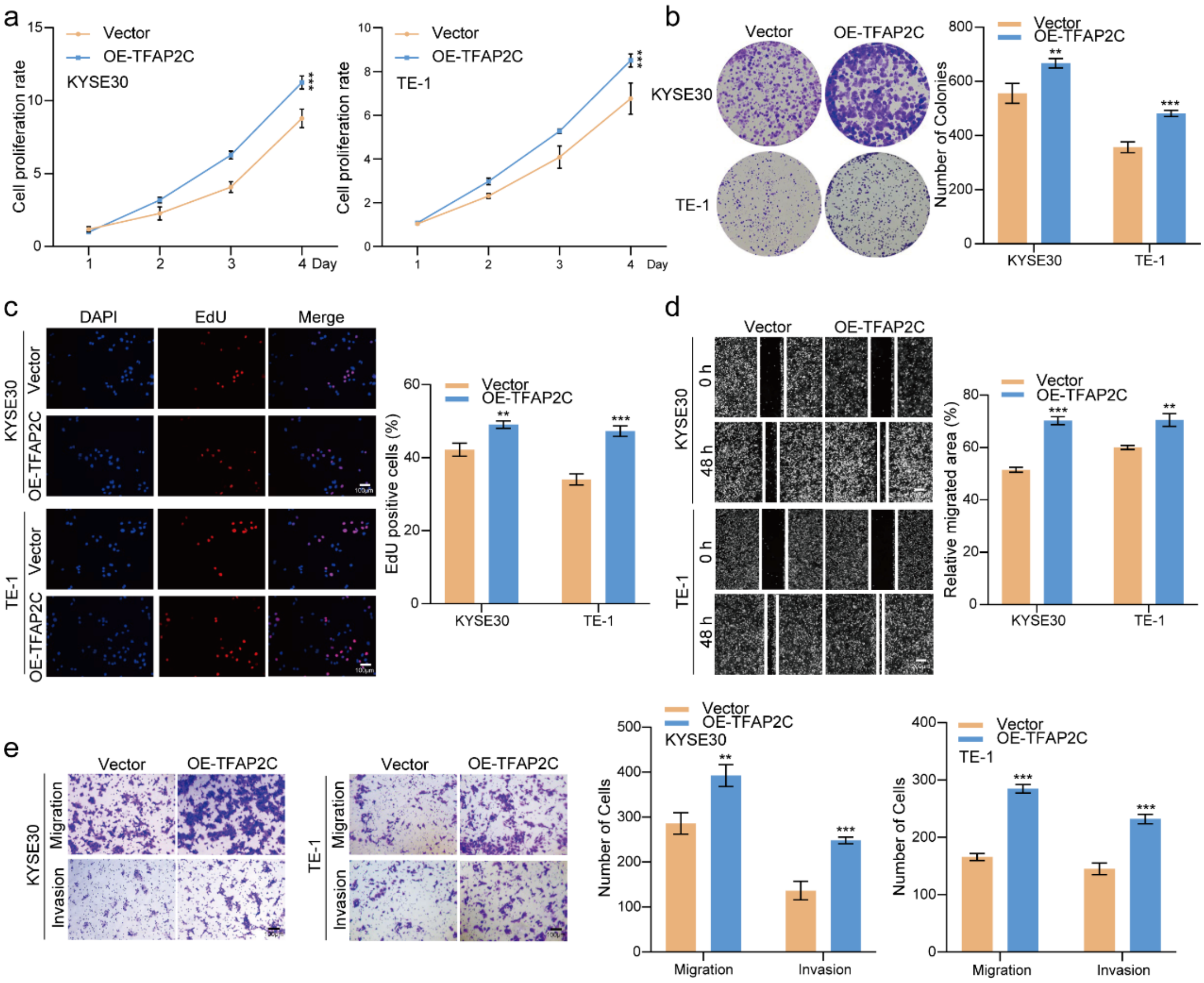

Supplementary Fig. S18 TFAP2C overexpression can promote proliferation and migration of esophageal squamous cell carcinoma cells, related to Fig. 6. The cell proliferation assay (**a**), clonogenic assay (**b**), EdU assay (**c**), wound healing assay (**d**), and Transwell assay (**e**) were performed in ESCC cells with TFAP2C overexpression compared to corresponding controls (n=3 for clonogenic assay, EdU assay, wound healing assay, and Transwell assay and n=6 for the cell proliferation assay). The P value was obtained by two-tailed unpaired Student's t-test (a-e). Data are presented as means ± SD unless otherwise stated. *p < 0.05, **p < 0.01, ***p < 0.001. ESCC, esophageal squamous cell carcinoma.

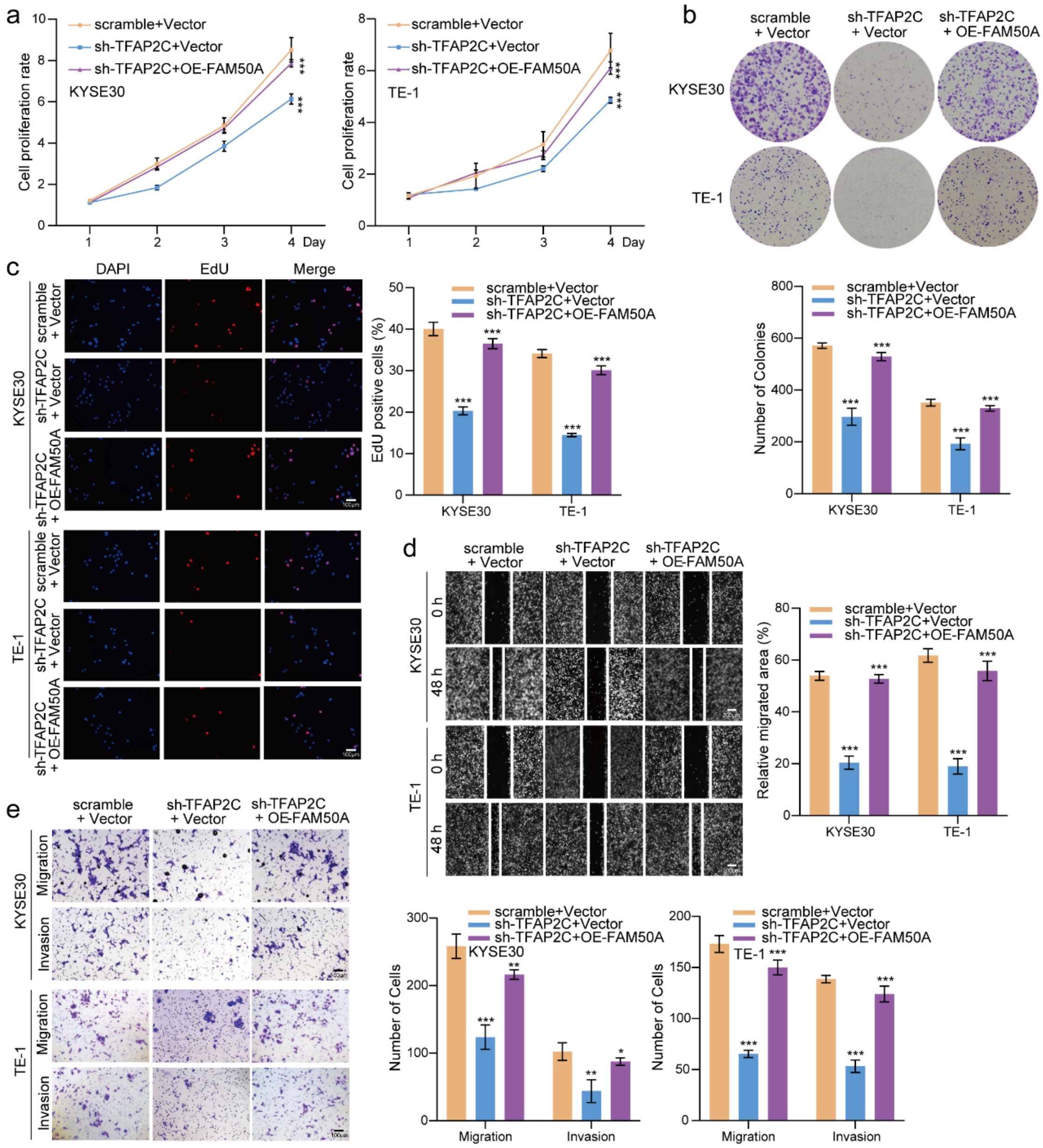

Supplementary Fig. S19 FAM50A overexpression can rescue the decreased proliferation and migration induced by TFAP2C knockdown, related to Fig. 6. The cell proliferation assay (**a**), clonogenic assay (**b**), EdU assay (**c**), wound healing assay (**d**), and Transwell assay (**e**) were performed in ESCC cells to investigate the role of FAM50A in rescuing the loss of TFAP2C (n=3 for clonogenic assay, EdU assay, wound healing assay, and Transwell assay and n=6 for cell proliferation assay). The P value was obtained by two-tailed unpaired Student's t-test (a-e). Data are presented as means ± SD unless otherwise stated. *p < 0.05, **p < 0.01, ***p < 0.001. ESCC, esophageal squamous cell carcinoma.

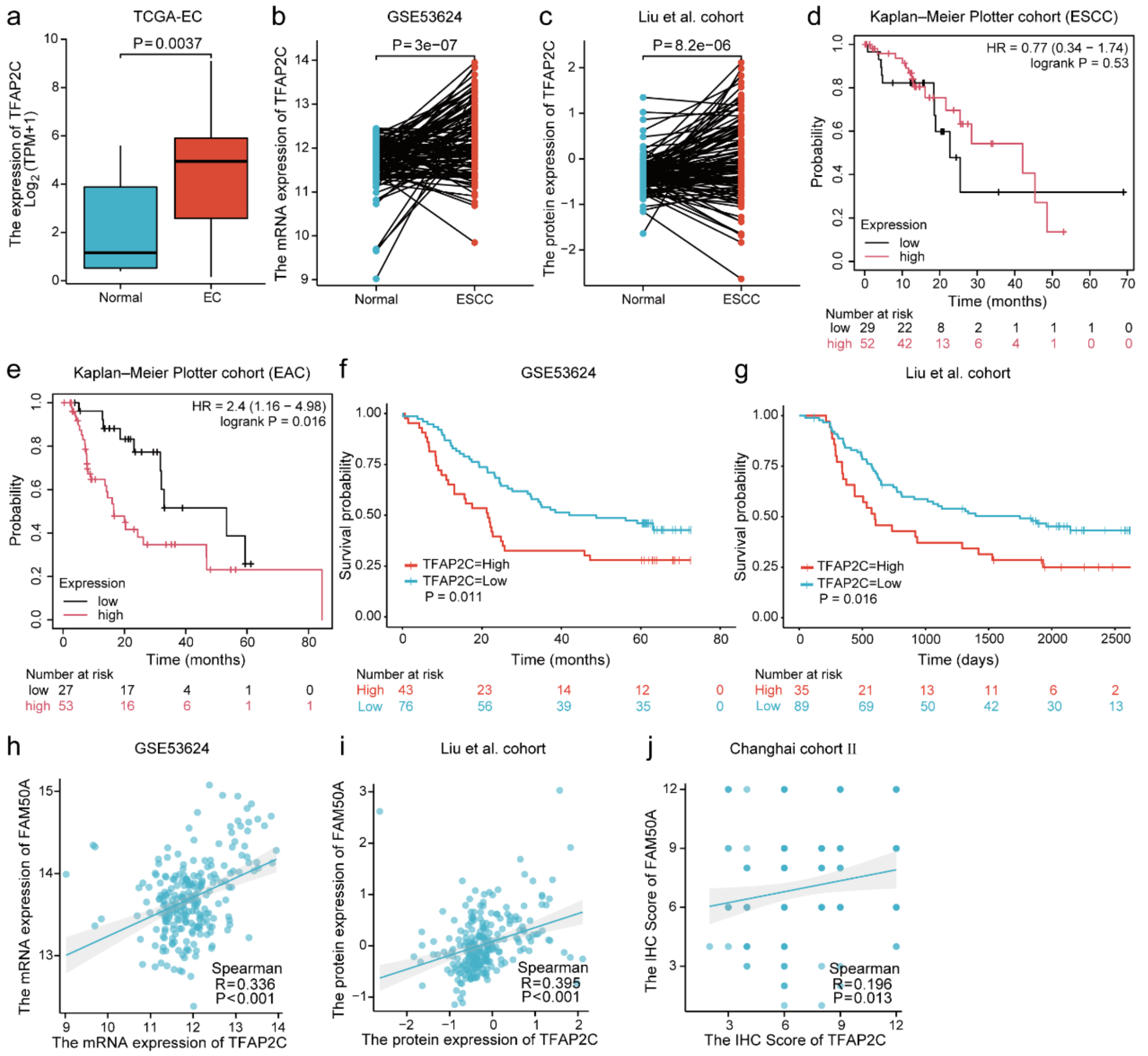

Supplementary Fig. S20 Upregulated TFAP2C is associated with progression and poor prognosis in esophageal squamous cell carcinoma, related to Fig. 6. a TFAP2C mRNA expression in TCGA-EC cohort. **b** TFAP2C mRNA expression in paired ESCC tissues in GSE53624 cohort. **c** TFAP2C protein expression in paired ESCC tissues in Liu et al. cohort. **d** Kaplan-Meier analysis of the correlation between TFAP2C expression and overall survival in TCGA-ESCC cohort. **e** Kaplan-Meier analysis of the correlation between TFAP2C expression and overall survival in TCGA-EAC cohort. **f** Kaplan-Meier analysis of the correlation between TFAP2C expression and overall survival in GSE53624 cohort. **g** Kaplan-Meier analysis of the correlation between TFAP2C expression and overall survival in Liu et al. cohort. **h** Correlation analysis of FAM50A and TFAP2C expression in GSE53624 cohort. **i** Correlation analysis of FAM50A and TFAP2C expression in Liu et al. cohort. **j** Correlation analysis of FAM50A and TFAP2C expression in Changhai cohort II. The P value was obtained by two-tailed unpaired Student’s t-test (a), two-tailed paired Student’s t-test (b, c), log-rank test (d-g), and Spearman correlation test (h-j). The optimal cut-off value in Kaplan-Meier analysis was determined using

the minimum P-value method. Data are presented as means ± SD unless otherwise stated. TCGA, The Cancer Genome Atlas Program; EC, esophageal cancer; ESCC, esophageal squamous cell carcinoma; EAC, esophageal adenocarcinoma.

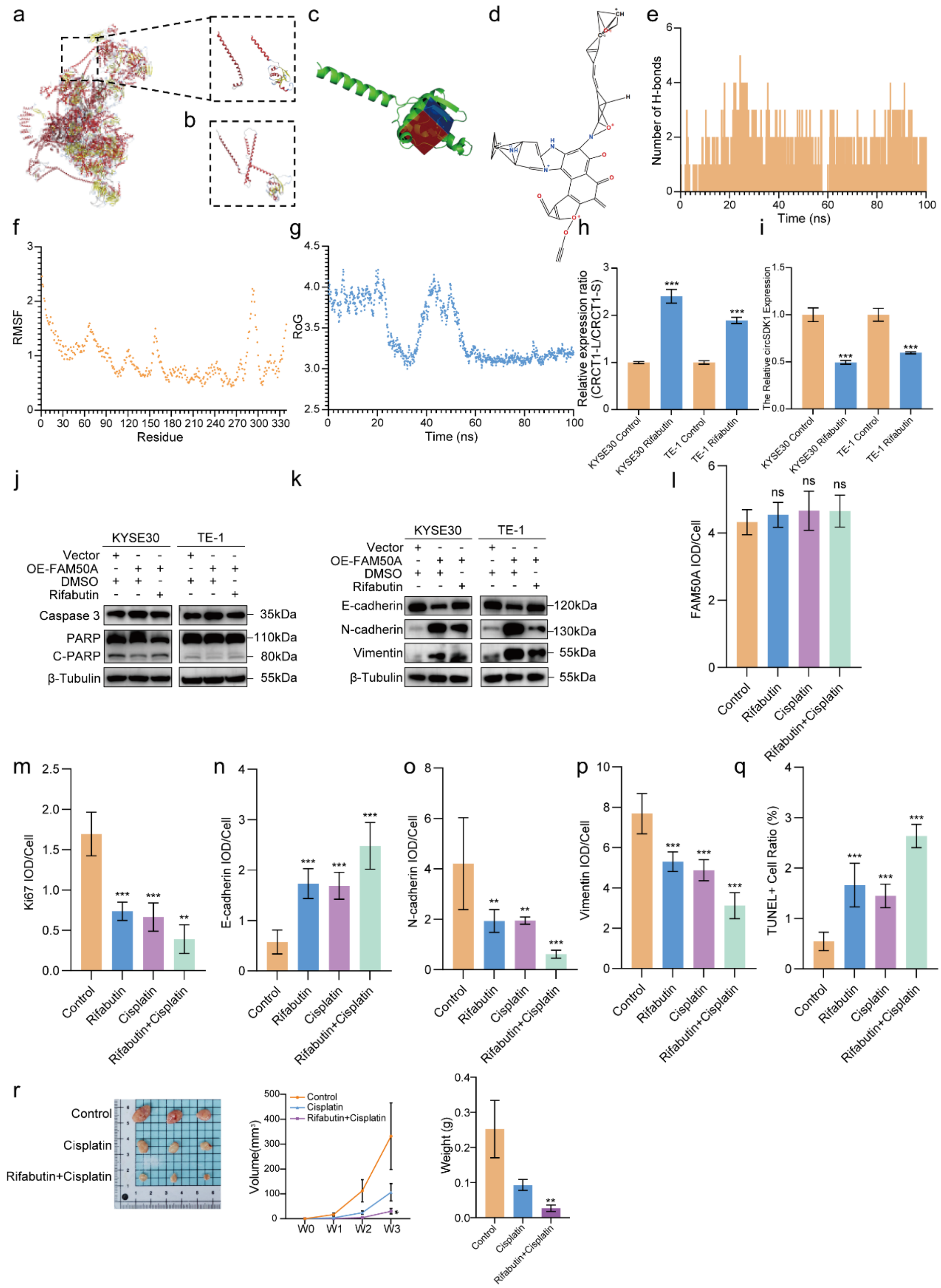

Supplementary Fig. S21 Virtual screen of small-molecule inhibitors of FAM50A, related to Fig. 7. a The crystal structure of FAM50A. **b** The AlphaFold predicted structure of FAM50A. **c** The docking box of FAM50A based on the predicted binding pocket. **d** Chemical structure formula of Rifabutin. **e** The number of hydrogen bonds between FAM50A and Rifabutin during molecular dynamics simulation. **f** The root-mean-square fluctuation of each residue of FAM50A during molecular dynamics simulation. **g** The radius of gyration of the FAM50A-Rifabutin complex during molecular dynamics simulation. **h** qPCR confirmation of the role of Rifabutin in FAM50A-dependent alternative splicing of CRTC1 (n=3). **i** qPCR confirmation of the role of Rifabutin in FAM50A-dependent backsplicing of circSDK1 (n=3). **j** Apoptotic markers were detected by Western Blot to investigate the role of Rifabutin. The cells were cultured in suspension condition. **k** EMT markers were detected by Western Blot to investigate the role of Rifabutin. Immunohistochemical quantification of FAM50A (**l**), Ki67 (**m**), E-cadherin (**n**), N-cadherin (**o**), and Vimentin (**p**), and TUNEL immunofluorescence assay (**q**) were performed in ESCC CDX models treated with Rifabutin, Cisplatin, or their combination, compared to corresponding DMSO controls (n=7 for all assays). **r** ESCC PDX dissected from NTG mice (n=3). Growth curves of tumor volumes were calculated in the PDX models (n=3). The weight of PDX dissected from NTG mice (n=3). The P value was obtained by two-tailed unpaired Student's t-test (h, i, l-r). Data are presented as means ± SD unless otherwise stated. $*p < 0.05$, $**p < 0.01$, $***p < 0.001$. EMT, epithelial-mesenchymal transition; ESCC, esophageal squamous cell carcinoma; PDX, patient-derived xenograft.

Supplementary Tables

Supplementary Table S1 Sequence Information.

Supplementary Table S2 Key resources table.

Supplementary Table S1. Sequence Information.

Method	Name	Sequence (5’-3’)
qPCR	FAM50A-F	GATGAGTCCCATGCAGGCAA
qPCR	FAM50A-R	GATGCTCAGCGGATCGTGTA
qPCR	DDX41-F	ATCCCACCACCCATCAAGAG
qPCR	DDX41-R	TGTCACGGCCAGATAGAATGG
qPCR	NOSIP-F	CCCAGAACATTCGACTGAGCC
qPCR	NOSIP-R	TGACAACAGGATCGTGGCAAG
qPCR	SF3B5-F	GCGACTCGTACTGCTCCTAC
qPCR	SF3B5-R	CTCGCGCTTTGCTCTCATTC
qPCR	CRTC1-L-F	GAACCAGATCGGGAGTGGCA
qPCR	CRTC1-L-R	AAGGGGGTCAGAGAGACAGGAG
qPCR	CRTC1-S-F	CAGCCGAGGCCAGTACTATG
qPCR	CRTC1-S-R	GATTGGAAGGGGGTCTGGAA
qPCR	TFAP2C-F	TACGAAGAGGACTGCGAGGA
qPCR	TFAP2C-R	AAGTAGGGTGGCGGCTGATA
qPCR	GAPDH-F	GGCTGTTGTCATACTTCTCATGG
qPCR	GAPDH-R	GGAGCGAGATCCCTCCAAAAT
qPCR	hsa-circ-0134225-div-F	ACTTGGTTTAGAGAAGGGCACAA
qPCR	hsa-circ-0134225-div-R	CGCATCCACTTGAACTCCAAAG
qPCR	hsa-circ-0134225-con-F	GATACGGACCAGAGGAAAACAGT
qPCR	hsa-circ-0134225-con-R	TTGTGCCCTTCTCTAAACCAAGT
qPCR	hsa-circ-0007482-div-F	GGCATCATCGTGTTTGGCAC
qPCR	hsa-circ-0007482-div-R	CGTGGCGCAAAAGCCTGT
qPCR	hsa-circ-0007482-con-F	GACCTACTCCTCCACAAACTC
qPCR	hsa-circ-0007482-con-R	GAGTCGGTCGTCTAGAGGAC
qPCR	U6-F	CTCGCTTCGGCAGCACA
qPCR	U6-R	AACGCTTCACGAATTTGCG
RIP-qPCR	CRTC1-exon3-F	GTGAACCAGATCGGGAGTGG
RIP-qPCR	CRTC1-exon3-R	CGTCCGTGTTTGTCCACTGA
RIP-qPCR	SDK1-intron1-F	GGGATGCAAGGTTGGTTCAAG
RIP-qPCR	SDK1-intron1-R	AGCTTTTGCCCATTCAATATGATGT
RIP-qPCR	circSDK1-F	ACTTGGTTTAGAGAAGGGCACAA

RIP-qPCR	circSDK1-R	CGCATCCACTTGAACTCCAAAG
ChIP-qPCR	FAM50A-promoter-site1-F	CCAGCCTTGGCTCGGGTTA
ChIP-qPCR	FAM50A-promoter-site1-R	GGTCCTGCGTGGACTCCC
ChIP-qPCR	FAM50A-promoter-site2-F	ACAAAGTTGCATTCCTCCCC
ChIP-qPCR	FAM50A-promoter-site2-R	GCAAATCAACCTCCAACGGG
ChIP-qPCR	FAM50A-promoter-site3-F	AGCCCATTGACCAGAAGTGA
ChIP-qPCR	FAM50A-promoter-site3-R	ATGCCCTTCCTGGTCCCTAA
ChIP-qPCR	FAM50A-promoter-site4-F	TGTGGCCAGACATCTACGTG
ChIP-qPCR	FAM50A-promoter-site4-R	CTTTTCAGGGTCCGTGGTCT
siRNA target	si-DDX41-1	CUAAGAGUGCCCUUGUAAATT
siRNA target	si-DDX41-2	GGAGAUUGAGAACUAUGUACA
siRNA target	si-NOSIP-1	CCUACCACGAGAAGAAGAATT
siRNA target	si-NOSIP-2	CAAGUAAGGACAAGGACAAAG
siRNA target	si-SF3B5-1	ACUUCGACCUUCUCAACUACU
siRNA target	si-SF3B5-2	ACUUCGACCUUCUCAACUACU
siRNA target	si-FAM50A-1	GCCAUGCACCUGAUGAAGATT
siRNA target	si-FAM50A-2	GAAGCCAAGCGGAAGAUCUTT
siRNA target	si-circSDK1-1	AUAAUGUACUUAUAUUCGCUG
siRNA target	si-circSDK1-2	UGAAACUUCCCAUAUAUGCGA
siRNA target	si-TFAP2C-1	TATTTAGACGTAGAGCTGAGG
siRNA target	si-TFAP2C-2	GCUCUACGUCUAAAUACAAAG
siRNA target	si-SDK1	GGAUUUCAGCUGAAGUAGAAG
siRNA target	si-NC	UUCUCCGAACGUGUCACGUTT
RNA probe	circSDK1 WT probe	AGGGCACAAGAUUAUUCCAAGCAACAGAAUAUGAU
RNA probe	circSDK1 MUT probe	AGGGCUGUUCUAAUAAGGUUCGUUGUCUUAAUGAU

Supplementary Table S2. Key resources table.

Reagent or Resource	Source	Identifier
**Antibodies**		
FAM50A Rabbit Monoclonal Antibody	Zenbio	R24279
β-tubulin Mouse Monoclonal Antibody	Epizyme	LF203
Ki-67 Rabbit Polyclonal Antibody	Proteintech	27309-1-AP
E-cadherin Rabbit Polyclonal Antibody	Proteintech	20874-1-AP
N-cadherin Rabbit Polyclonal Antibody	Proteintech	22018-1-AP
Vimentin Rabbit Polyclonal Antibody	Epizyme	P900008
ERK1/2 Rabbit Polyclonal Antibody	Beyotime	AB2014
Phospho-ERK1/2 Rabbit Monoclonal Antibody	Beyotime	AF1891
P38 Rabbit Monoclonal Antibody	Beyotime	AF7668
Phospho-P38 Rabbit Polyclonal Antibody	Beyotime	AF5884
JNK1/2/3 Rabbit Monoclonal Antibody	Beyotime	AF1048
Phospho-JNK1/2/3 Rabbit Monoclonal Antibody	Beyotime	AF1762
FAK Rabbit Monoclonal Antibody	Zenbio	R24276
Phospho-FAK (Tyr397) Rabbit Monoclonal Antibody	Zenbio	R381143
Phospho-FAK (Tyr576+Tyr577) Rabbit Polyclonal Antibody	Zenbio	680581
PARP1 Rabbit Polyclonal Antibody	Proteintech	13371-1-AP
Caspase 3/P17/P19 Rabbit Polyclonal Antibody	Proteintech	19677-1-AP
HNRNPL Rabbit Polyclonal Antibody	Proteintech	18354-1-AP
EFTUD2 Rabbit Polyclonal Antibody	Proteintech	10208-1-AP
HNRNPC Rabbit Polyclonal Antibody	Proteintech	11760-1-AP
DDX41 Rabbit Polyclonal Antibody	Proteintech	27500-1-AP
HIF1 alpha Rabbit Polyclonal Antibody	Zenbio	340462
SC35 Mouse Monoclonal Antibody	Abcam	ab11826
DYKDDDDK tag Mouse Polyclonal Antibody	Proteintech	66008-4-Ig

TFAP2C Rabbit Polyclonal Antibody	Proteintech	14572-1-AP
DUSP22 Mouse Monoclonal Antibody	Proteintech	68032-1-Ig
PCNA Rabbit Polyclonal Antibody	Proteintech	10205-2-AP
E-cadherin Rat Monoclonal Antibody	Proteintech	65241-1-Ig
N-cadherin Mouse Monoclonal Antibody	Proteintech	66219-1-Ig
DDX41 Polyclonal antibody	Proteintech	27500-1-AP
NOSIP Polyclonal antibody	Proteintech	27979-1-AP
SF3B5 Polyclonal antibody	Proteintech	15525-1-AP
AKT Polyclonal antibody	Proteintech	10176-2-AP
Phospho-AKT (Ser473) Monoclonal antibody	Proteintech	66444-1-Ig
Phospho-AKT (Thr308) Recombinant monoclonal antibody	Proteintech	81232-10-RR
NF-κB p65 Polyclonal antibody	Proteintech	10745-1-AP
Phospho-NF-κB p65 (Ser536) Recombinant monoclonal antibod	Proteintech	80379-2-RR
**Bacterial and virus strains**		
pSLenti-U6-shRNA (NC2)-CMV-EGFP-F2A-Puro-WPRE	OBiO	N/A
pSLenti-U6-shRNA (FAM50A#1)-CMV-EGFP-F2A-Puro-WPRE	OBiO	N/A
pSLenti-U6-shRNA (FAM50A#2)-CMV-EGFP-F2A-Puro-WPRE	OBiO	N/A
pcSLenti-EF1-EGFP-F2A-Puro-CMV-MCS-WPRE	OBiO	N/A
pcSLenti-EF1-EGFP-F2A-Puro-CMV-FAM50A-WPRE	OBiO	N/A
pSLenti-U6-shRNA (circSDK1#1)-CMV-EGFP-F2A-Puro-WPRE	OBiO	N/A
pSLenti-U6-shRNA (circSDK1#2)-CMV-EGFP-F2A-Puro-WPRE	OBiO	N/A
pLC5-ciR-CMV-EF1a-copGFP-BSD-WPRE	Geneseed	N/A
pLC5-ciR-CMV-circSDK1-EF1a-copGFP-	Geneseed	N/A

BSD-WPRE		
pcSLenti-EF1-EGFP-F2A-Puro-CMV-DUSP22-WPRE	OBiO	N/A
pSLenti-U6-shRNA (TFAP2C#1)-CMV-EGFP-F2A-Puro-WPRE	OBiO	N/A
pSLenti-U6-shRNA (TFAP2C#2)-CMV-EGFP-F2A-BSD-WPRE	OBiO	N/A
pSLenti-U6-shRNA (TFAP2C#2)-CMV-EGFP-F2A-BSD-WPRE	OBiO	N/A
pcSLenti-EF1-EGFP-F2A-Puro-CMV-TFAP2C-WPRE	OBiO	N/A
pcSLenti-EF1-EGFP-F2A-Puro-CMV-SDK1-Res-WPRE	OBiO	N/A
pLC5-ciR-CMV-circSDK1-silMut-EF1a-copGFP-BSD-WPRE	Geneseed	N/A
**Biological samples**		
ESCC tumor specimens	Changhai Hospital	N/A
**Chemicals, peptides, and recombinant proteins**		
RPMI 1640	Gibco	C11875500BT
Fetal Bovine Serum	Gibco	A5256701
Penicillin-streptomycin, 100×	Gibco	10378016
PBS	Gibco	14190144
Albumin Bovine	BioFroxx	4240GR100
Lipofectamine™ RNAiMAX	Invitrogen	13778150
Lipofectamine™ 3000	Invitrogen	L3000015
TRIzol	Invitrogen	15596018CN
Opti-MEM	Gibco	51985034
Immobilon®-P PVDF Membrane	Millipore	IPVH00010
RIPA Lysis Buffer	TargetMol	C0045
Skim Milk (Blotting Grade)	Beyotime	P0216
Cell Counting Kit-8	TargetMol	C0005
Transwell chamber	Falcon	353097
Matrigel® Matrix	Corning	356231

Collagen, Type I	Yeasen	40125ES
Fibronectin	Roche	10838039001
Laminin	Sigma	L4544
96-well Clear Round Bottom Ultra-Low Attachment Microplate	Corning	7007
Pierce™ IP lysis buffer	Thermo Fisher Scientific	87788
PureProteome™ Protein A/G Mix Magnetic Beads	Millipore	LSKMAGAG02
Esophageal Squamous Cell Carcinoma Organoid Complete Medium	SHRbio	ESOM-100
Crystal violet	Solarbio	C8470
Trypsin without ethylenediaminetetraacetic acid (EDTA)	Macgene	CC035
Tumor Tissue Digestion Solution	SHRbio	TTD-50
Organoid Passage & Digestion Solution	SHRbio	OPD-100
Cisplatin	TargetMol	15663-27-1
Palovarotene	TargetMol	410528-02-8
Rifabutin	TargetMol	72559-06-9
Hypericin	TargetMol	548-04-9
Temoporfin	TargetMol	122341-38-2
Triton X-100	Solarbio	T8200
Puromycin Dihydrochloride	Beyotime	ST551
Blasticidin S HCl	Beyotime	ST018
RNase R	Epicenter	28992
Actinomycin D	MedChemExpress	HY-17559
(E)-Osmundacetone	MedChemExpress	HY-N1966
Y15	MedChemExpress	HY-12444
Recombinant GST-tagged DUSP22 protein	CUSABIO	CSB-EP865123HU
**Critical commercial assays**		
Evo M-MLV RT Kit	Accurate Biotechnology	AG11705
SYBR Green Premix Pro Taq HS qPCR Kit	Accurate Biotechnology	AG11701

BeyoClick™ EdU Cell Proliferation Kit with AF555	Beyotime	C0075L
Trypan Blue Staining Cell Viability Assay Kit	Beyotime	C0011M
Caspase 3 Activity Assay Kit	Beyotime	C1116
TUNEL Cell Apoptosis Detection Kit	Aifang	AFIHC030
Fluorescence In Situ Hybridization Kit	RiboBio	C10910
RNAmax-T7 In Vitro Transcription Kit	RiboBio	C11001-2
Pierce™ RNA 3' End Desthiobiotinylation Kit	Thermo Fisher Scientific	20163
BersinBio™ RNA pull-down Kit	BersinBio	Bes5102
BersinBio™ ChIP kit	BersinBio	Bes5001
Dual Luciferase Reporter Gene Assay Kit	Beyotime	RG027
BersinBio™ RNA Immunoprecipitation Kit	BersinBio	Bes5101
Ribo-Zero rRNA Removal Kit	Illumina	20040526
Cytoplasmic & Nuclear RNA Purification Kit	Norgen Biotek	21000
Chemiluminescent EMSA Kit	Beyotime	GS009
**Deposited data**		
TCGA-EC cohort	N/A	TCGA
GSE53624 cohort	N/A	GEO: GSE53624
Liu et al. cohort	Liu et al. (PMID: 34400640)	PRIDE: PXD021701
Changhai cohort I	This paper	PRIDE: PXD063341
RNA-seq of FAM50A knockdown by shRNA in ESCC cells	This paper	GSA-Human: HRA012618
circRNA-seq of FAM50A knockdown by shRNA in ESCC cells	This paper	GSA-Human: HRA012618
RIP-seq of FAM50A in ESCC cells	This paper	GSA-Human: HRA012619
circRNA-seq of four paired ESCC tissues	This paper	GSA-Human: HRA012617
IP-MS of FAM50A in ESCC cells	This paper	OMIX: OMIX011253

**Experimental models: Cell lines**		
KYSE510	Procell	CL-0737
KYSE450	FuHeng	FH0597
KYSE410	Procell	CL-0586
KYSE180	Procell	CL-0760
KYSE150	Procell	CL-0638
KYSE30	Procell	CL-0577
Eca109	Procell	CL-0077
TE-1	Chinese Academy of Sciences	TCHu89
BE-3	JsCall	JSY-CC3841
HET-1A	American Type Culture Collection	CRL-2692
**Experimental models: Organisms/strains**		
Nude Mouse (BALB/c-nu)	SiPeiFu	N/A
NTG Mouse (NOD-SCID-IL2rgKO)	SiPeiFu	N/A
**Oligonucleotides**		
siRNA, see Supplementary Table 1	Accurate Biotechnology	N/A
qPCR primers, see Supplementary Table 1	Accurate Biotechnology	N/A
circRNA probe, see Supplementary Table 1	GeneCreate	N/A
**Recombinant DNA**		
pCDNA3.1-CMV-MCS-3xFLAG-hGHpolya-EF1a-EGFP	OBiO	N/A
pCDNA3.1-CMV-FAM50A(Δ169-339)-3xFLAG-hGHpolya-EF1a-EGFP	OBiO	N/A
pCDNA3.1-CMV-FAM50A(Δ68-168)-3xFLAG-hGHpolya-EF1a-EGFP	OBiO	N/A
pCDNA3.1-CMV-FAM50A(Δ1-67)-3xFLAG-hGHpolya-EF1a-EGFP	OBiO	N/A
pCDNA3.1-CMV-FAM50A(WT)-3xFLAG-hGHpolya-EF1a-EGFP	OBiO	N/A
pCDNA3.1-CMV-DUSP22(Δ145-184)-	OBiO	N/A

3xFLAG-hGHpolya-EF1a-EGFP		
pCDNA3.1-CMV-DUSP22(Δ1-144)-3xFLAG-hGHpolya-EF1a-EGFP	OBiO	N/A
pCDNA3.1-CMV-DUSP22(WT)-3xFLAG-hGHpolya-EF1a-EGFP	OBiO	N/A
pCDNA3.1-CMV-HIF1α-3xFLAG-hGHpolya-EF1a-EGFP	OBiO	N/A
pCDNA3.1-CMV-DUSP22(M1A,N3A,D22A,Q25A,R133A)-3xFLAG-hGHpolya-EF1a-EGFP	OBiO	N/A
**Software and algorithms**		
FastQC v0.11.9	N/A	https://www.bioinformatics.babraham.ac.uk/projects/fastqc/
Trimgalore v0.6.1	N/A	https://www.bioinformatics.babraham.ac.uk/projects/trim_galore/
bowtie v1.2.3	Langmead et al. (PMID: 19261174)	http://bowtiebio.sourceforge.net/index.shtml
STAR v2.7.1a	Dobin et al. (PMID: 23104886)	https://github.com/alexdobin/STAR
HISAT2 v2.2.0	Zhang et al. (PMID: 34103331)	http://daehwankimlab.github.io/hisat2/
HISAT2 v2.2.0	Zhang et al. (PMID: 34103331)	http://daehwankimlab.github.io/hisat2/
Salmon v0.6.0	Patro et al. (PMID: 28263959)	https://combinelab.github.io/salm on/
Picard v2.25.5	N/A	https://gatk.broadinstitute.org/hc/enus/

		articles/360057439771MarkDuplicatesPicard
StringTie v1.3.0	Kovaka S et al. (PMID: 31842956)	http://ccb.jhu.edu/software/stringtie/
featureCounts v2.0.0	Liao Y et al.	http://subread.sourceforge.net/featureCounts.html
rMATS v4.1.0	Shen et al. (PMID: 25480548)	http://rnaseqmats.sourceforge. net/
edgeR v3.34.0	Robinson et al. (PMID: 19910308)	https://bioconductor.org/packages/release/bioc/html/edgeR.html
CIRI2	Zhang et al. (PMID: 31900416)	https://ciri-cookbook.readthedocs.io/en/latest/index.html
Survival v3.2-11	N/A	https://cran.rproject.org/web/packages/survival/index.html
Survminer v0.4.9	N/A	https:// github.com/kassambara/survminer/
ggplot2 v3.3.5	N/A	https://ggplot2.tidyverse.org
HOMER v4.11	Heinz et al. (PMID: 20513432)	http://homer.ucsd.edu/homer/
deeptools v3.1.3	N/A	https://github.com/jernejule/clipdata-science
PANTHER	N/A	http://pantherdb.org/

Gene Ontology	The Gene Ontology (PMID: 30395331)	http://geneontology.org/
Kaplan-Meier plotter	N/A	http://kmplot.com/analysis/
R/Bioconductor v4.4.1	N/A	http://www.rproject.org/
GraphPad Prism v10.1.2	N/A	https://www.graphpad.com/scientific-software/prism/
ImageJ v1.54f	Schneider et al., 2012 (PMID: 22930834)	https://imagej.nih.gov/ij/
Integrative Genomics Viewer (IGV) v2.16.0	Robinson et al., 2017 (PMID: 29092934)	https://igv.org/
